# Safety and biological outcomes following a phase 1 trial of GD2-specific CAR-T cells in patients with GD2-positive metastatic melanoma and other solid cancers

**DOI:** 10.1136/jitc-2023-008659

**Published:** 2024-05-15

**Authors:** Tessa Gargett, Nga T H Truong, Bryan Gardam, Wenbo Yu, Lisa M Ebert, Amy Johnson, Erica C F Yeo, Nicole L Wittwer, Gonzalo Tapia Rico, Jesikah Logan, Purany Sivaloganathan, Maria Collis, Andrew Ruszkiewicz, Michael P Brown

**Affiliations:** 1 University of South Australia, Translational Oncology Laboratory, Centre for Cancer Biology, SA Pathology, Rundle Mall, South Australia, Australia; 2 Adelaide Medical School, The University of Adelaide, Adelaide, South Australia, Australia; 3 Cancer Clinical Trials Unit, Royal Adelaide Hospital, Adelaide, South Australia, Australia; 4 Flinders University, Adelaide, South Australia, Australia; 5 Surgical Pathology, SA Pathology, Adelaide, South Australia, Australia; 6 School of Pharmacy and Medical Science, University of South Australia, Adelaide, South Australia, Australia

**Keywords:** Chimeric antigen receptor - CAR, Combination therapy, Solid tumor

## Abstract

**Background:**

Chimeric antigen receptor (CAR) T cell therapies specific for the CD19 and B-cell maturation antigen have become an approved standard of care worldwide for relapsed and refractory B-cell malignancies. If CAR-T cell therapy for non-hematological malignancies is to achieve the same stage of clinical development, then iterative early-phase clinical testing can add value to the clinical development process for evaluating CAR-T cell products containing different CAR designs and manufactured under differing conditions.

**Methods:**

We conducted a phase 1 trial of third-generation GD2-specific CAR-T cell therapy, which has previously been tested in neuroblastoma patients. In this study, the GD2-CAR-T therapy was evaluated for the first time in metastatic melanoma patients in combination with BRAF/MEK inhibitor therapy, and as a monotherapy in patients with colorectal cancer and a patient with fibromyxoid sarcoma. Feasibility and safety were determined and persistence studies, multiplex cytokine arrays on sera and detailed immune phenotyping of the original CAR-T products, the circulating CAR-T cells, and, in select patients, the tumor-infiltrating CAR-T cells were performed.

**Results:**

We demonstrate the feasibility of manufacturing CAR-T products at point of care for patients with solid cancer and show that a single intravenous infusion was well tolerated with no dose-limiting toxicities or severe adverse events. In addition, we note significant improvements in CAR-T cell immune phenotype, and expansion when a modified manufacturing procedure was adopted for the latter 6 patients recruited to this 12-patient trial. We also show evidence of CAR-T cell-mediated immune activity and in some patients expanded subsets of circulating myeloid cells after CAR-T cell therapy.

**Conclusions:**

This is the first report of third-generation GD2-targeting CAR-T cells in patients with metastatic melanoma and other solid cancers such as colorectal cancer, showing feasibility, safety and immune activity, but limited clinical effect.

**Trial registration number:**

ACTRN12613000198729.

**WHAT IS ALREADY KNOWN ON THIS TOPIC**

- GD2 is a clinically validated cancer immunotherapy target antigen and chimeric antigen receptor (CAR) T cell-targeting of GD2 has shown clinically significant activity in neuroblastoma and diffuse midline glioma patients. GD2 is also widely expressed in other neuroectodermal tumors, sarcomas, and lung tumors but associated clinical data and knowledge of factors constraining GD2-CAR-T cell expansion and persistence in patients with solid cancer are limited.

**WHAT THIS STUDY ADDS**

- This is the first report of GD2-CAR-T cell therapy in patients with metastatic colorectal cancer and advanced melanoma patients treated concurrently with BRAF and MEK kinase inhibitors. The detailed immunobiological analyses add to a growing understanding of factors influencing CAR-T cell expansion and persistence in patients with solid cancer. An ex vivo manufacturing method favoring generation of naïve-like CAR-T cells results in CAR-T cell expansion in vivo.

**HOW THIS STUDY MIGHT AFFECT RESEARCH, PRACTICE OR POLICY**

- This study suggests that robust CAR-T cell activity engenders protumorigenic myeloid cells, thus constraining effective CAR-T cell therapy, and further identifies the need to improve GD2-CAR-T cell therapy by both refining patient selection and CAR vector design.

## Background

Clinical and preclinical research in the field of chimeric antigen receptor (CAR) T cell therapies has exploded in the last decade. Alongside clinical development of CD19-targeted CAR-T cell therapy of renowned success was CAR-T cell therapy targeting the glycolipid antigen GD2.^1^ GD2 is a disialoganglioside expressed in tumors of neuroectodermal origin, and a tumor antigen of significant interest with elevated expression reported in neuroblastoma, melanoma, retinoblastoma, gliomas, small cell lung cancer, and sarcoma including Ewing sarcoma, osteosarcoma and soft tissue sarcoma.^2–6^ The GD2-targeting monoclonal antibody dinutuximab is US Food and Drug Administration approved as part of postconsolidation therapy for neuroblastoma patients.^7^

The first generation of GD2-CAR-T cell therapy was developed at Baylor College of Medicine (Houston, Texas, USA), and the CAR incorporated the 14g2a-derived single-chain variable fragment (scFv) with the CD3ζ signaling domain. This CAR construct was introduced into Epstein-Barr virus-specific or activated T cells using a retroviral vector. The resulting GD2-CAR-T cells were tested in 19 neuroblastoma patients.^8^ Five patients with active disease showed tumor responses or necrosis, with three patients demonstrating complete responses. Of note, progression-free survival was strongly correlated with persistence of GD2-CAR T cells beyond 6 weeks, even though these cells were only present long term at low levels. Prolonged GD2-CAR T cell persistence in peripheral blood was associated with the presence of both CD4+T cells and central memory T cells in the infused product. Although the aim of employing virus-specific T cells in this study was to provide physiological T-cell stimulation, costimulation required for T-cell survival, expansion and cytotoxic activity may also be provided by adding appropriate signaling domains to the CAR construct.^9 10^

In a comparative preclinical study of CD3zeta, CD28-CD3zeta and CD28-OX40-CD3zeta endodomains of GD2-specific CAR, the CD28-OX40-CD3zeta construct optimized the proliferation, survival and cytolytic capacity of transgenic T cells.^10^ The sustained cytotoxic functions of the CD28-OX40-CD3zeta containing GD2-CAR manifested as significant antitumor effects of GD2-CAR gene-modified human T cells in a murine model of GD2-expressing human melanoma metastases.^9^ Importantly, melanoma cell killing by these GD2-CAR gene-modified T cells occurred even when GD2 expression was relatively low.^9^

These third-generation GD2-CAR-T cells (referred to in this study as GD2-iCAR-PBT; in recognition of the (i)nducible caspase 9 suicide gene encoded upstream of the CAR domain) have been tested in the GRAIN trial for neuroblastoma patients (NCT01822652).^11^ In the GRAIN trial, 11 patients were treated in three non-randomized cohorts: Cohort 1—GD2-iCAR-PBT alone, cohort 2—GD2-iCAR-PBT with prior cyclophosphamide and fludarabine (Cy/Flu) lymphodepletion chemotherapy, and cohort 3—Cy/Flu conditioning, GD2-iCAR-PBT, and the programmed death-1 (PD-1) inhibitor, pembrolizumab. GD2-iCAR-PBT persistence was limited and responses of progressive disease or disease stabilization were observed. Infusions were well tolerated and neurological adverse events (AEs) in these patients were minimal, despite the known low-level expression of GD2 by neurons and the documented peripheral neurotoxicity of the GD2-targeting monoclonal antibody.^12^ Other findings were that lymphodepletion chemotherapy contributed significantly to CAR-T cell expansion, but not the addition of pembrolizumab, and that a blood subpopulation of presumably immunosuppressive CD45/CD33/CD11b/CD163+myeloid cells also expanded in all patients after the CAR-T cell infusion. Recently, a trial of 13 osteosarcoma and neuroblastoma patients using an identical third-generation CAR was reported. This GD2-CAR-T cell therapy showed no dose-limiting toxicity (DLT) and disease stabilization in 10 of 12 patients^13^ and a myeloid signature associated with poor CAR-T cell expansion was also identified.

GD2-specific CAR constructs derived from a humanized 3F8 scFv have since been tested in glioblastoma patients^14^ with partial responses reported in four of eight patients. Recently, in trials of GD2-CAR-T cell therapy also using the 14g2a scFv, an impressive 17 of 27 relapsed/refractory neuroblastoma patients showed a response^15^ and 4 patients with diffuse midline glioma (DMG) had evidence of a radiological response and clinical benefit in a preliminary report.^16^ Interestingly, also in this study,^16^ a myeloid-derived suppressor cell (MDSC) phenotype was more apparent in Cerebrospinal fluid (CSF) after intravenous rather than intracerebroventricular administration of GD2-CAR-T cells.

Despite its clinical promise, open questions about the in vivo persistence and lasting effectiveness of GD2-CAR-T cell therapy remain as they do generally for CAR-T cell therapy for patients with solid cancer. Examples include questions about optimal vector design,^17 17^ arming with immune-active cytokines,^3 18^ and defences against potential immune-inhibitory effects of myeloid-derived suppressor cells.^11 16 19^ Interest has also arisen in combining immunotherapy with small molecule kinase inhibitors that are the mainstay of many therapeutic anti-cancer regimens.

Now, in the CARPETS (Phase 1 study of GD2 Chimeric Antigen Receptor-Expressing PEripheral Blood T CellS) trial, we have tested GD2-iCAR-PBT therapy in patients with melanoma and other solid cancers. The treatment of metastatic melanoma has been transformed by immune checkpoint inhibitor therapy, although at least 40% of patients will not obtain durable benefit from these approved immunotherapies.^20 21^ Therefore, an unmet clinical need for other immunotherapies exists. To address concerns about CAR-T cell persistence in vivo, we investigated methods for generating more long-lived CAR-T cells by changing the activation and culture parameters used for ex vivo manufacture.^22 23^ Predicated on clinical observations of T-cell infiltration of V600-BRAF mutant melanoma after oncogene suppression using BRAF kinase with or without MEK kinase inhibition,^24 25^ and potentially favorable interactions with antitumor T cells observed in preclinical studies,^26 27^ we also investigated combining GD2-CAR-T cell therapy with targeted therapy using BRAF and MEK kinase inhibitors, dabrafenib and trametinib, respectively.^28 29^ The primary objectives were to determine the safety and feasibility of treating this patient population with CAR-T cells manufactured at point of care. Secondary objectives included documentation of anticancer responses, and the evaluation of the in vivo persistence of CAR-T cells together with biomarkers of their activity.

## Results

### Patient recruitment, demographics and baseline characteristics

**Figure 1:**
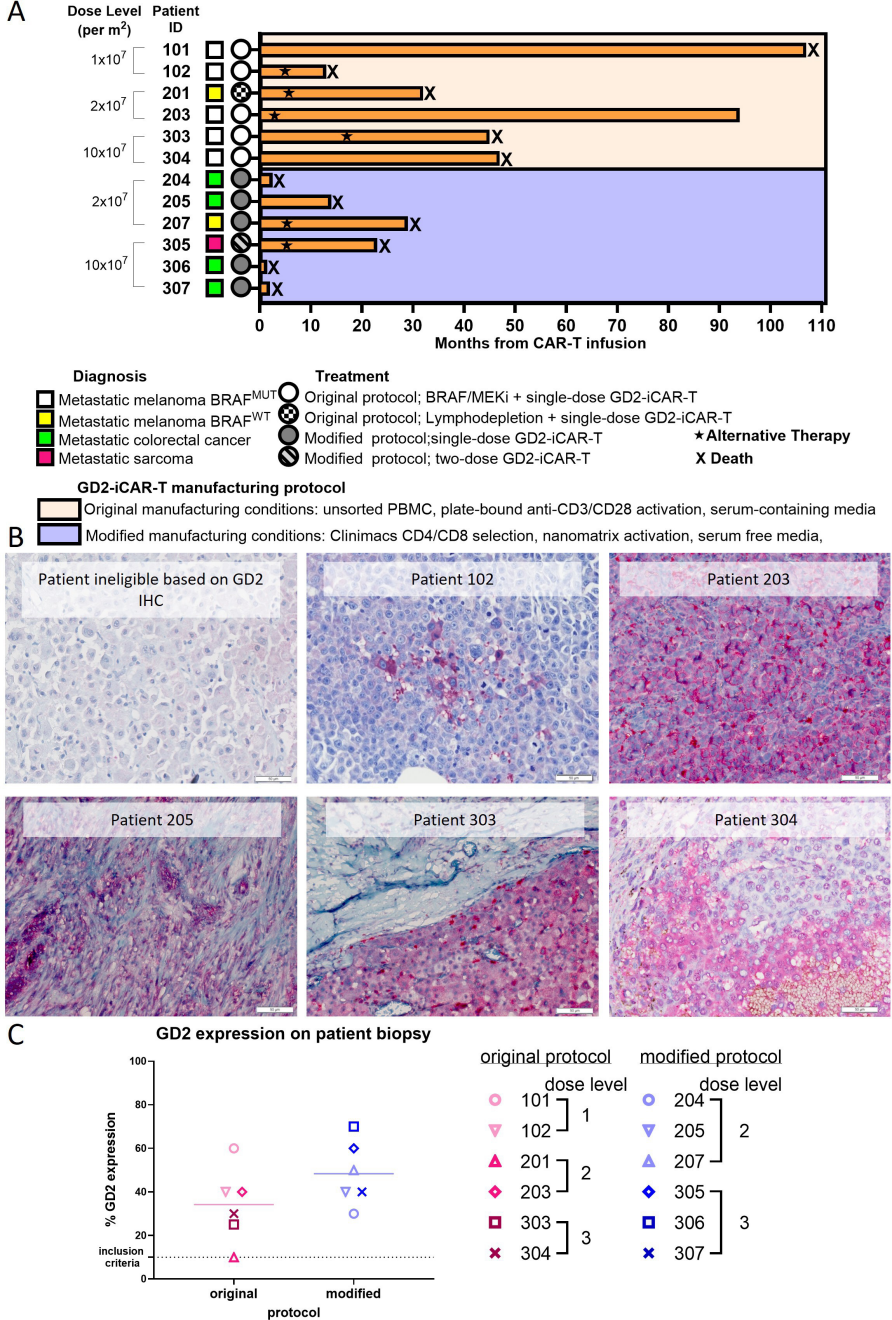
Tumor expression of GD2 in CARPETS study patients. Archived patient tumor tissues (formalin-fixed, paraffin embedded; FFPE) collected for diagnostic pathology were screened for GD2 expression, with an inclusion criterion of ≥10% tumor-cell GD2 expression. (A) Swimmer plot showing outcomes after GD2-CAR-T cell infusions in patients with metastatic melanoma (with or without BRAF/MEK inhibitor therapy), metastatic colorectal cancer and metastatic sarcoma. (B) Examples of GD2 expression detected by immunohistochemistry on FFPE archival tumor tissues at diagnosis. (C) Summary of tumor GD2 expression levels in patients as reported by an independent pathologist. Metastatic melanoma patients treated using the original manufacturing protocol (pink symbols), metastatic melanoma and other solid cancer patients treated using the modified protocol (blue symbols). Significance was assessed by unpaired t test, GraphPad Prism V.10.1.1. CAR, chimeric antigen receptor; CARPETS, Phase 1 study of GD2 Chimeric Antigen Receptor-Expressing PEripheral Blood T CellS.

This was a phase 1 clinical study in which V600 BRAF mutant metastatic melanoma patients, who were receiving concurrent treatment using the standard BRAF/MEK inhibitor combination of dabrafenib and trametinib, received intravenous infusions of GD2-iCAR-PBT products. A later clinical protocol amendment allowed enrolment of patients with other GD2-positive solid cancers. 24 patients were prescreened, and their archival tumor biopsy samples were evaluated for GD2 expression by immunohistochemistry (IHC). GD2 expression values ranged from 10% to 70% (figure 1). 14 of these patients were eligible based on GD2 expression and consented to participate in the main study (online supplemental figure S1). Of these 14 patients, 12 patients received the allocated intervention between December 2014 and December 2021. The patient’s age ranged from 35 to 74 years. 13 participants were white Caucasian, and 1 was Maori. 11 males and 3 females were enrolled. Six patients had an ECOG performance status (PS) of 0 at screening and eight had an Eastern Cooperative Oncology Group (ECOG) PS of 1 (table 1). Other subsequently treated patients with GD2-positive solid cancers, who included four metastatic colorectal cancer (mCRC) patients and a patient with fibromyxoid sarcoma, had a lower average performance status at the time of CAR-T cell product administration. All four mCRC patients had evidence of cancer inflammation at screening and preinfusion, which was represented by the pan-immune inflammation value^30^ (online supplemental figure S2A). Interestingly, three of these four patients survived fewer than 3 months from the CAR-T cell infusion.

10.1136/jitc-2023-008659.supp1 Supplementary data

**Table 1:** Patient demographics, study cohorts by cell dose level, and tumor responses

Cell dose level	Patient ID	Ethnicity	ECOG	Stage	Cancer type	BRAF/MEKi	CAR-T cell manufacturing protocol	RECIST1.1 @ 42 days	Survival time (months)	Survival status (January 2024)
1 1×10^7^/m^2^	101	White	0	IV M1d	mMel-MT	Initially dabrafenib	Riginal	PR	108	DoD
	102	White	0	IV M1b	mMel-MT	Yes	Riginal	PR	13	DoD
2 2×10^7^/m^2^	201*	Maori	0	IV M1a	mMel-WT	No	Riginal	SD	32	DoD
	202†	White	1	IV M1d	mMel-MT	N/A	Riginal	N/A	N/A	N/A
	203	White	0	IV M1c	mMel-MT	Yes	Riginal	PR	94	NED‡
3 1×10^8^/m^2^	303	White	1	IIIC unresectable	mMel-MT	Yes	Riginal	PR	45	DoD
	304	White	1	IV M1d	mMel-MT	Yes	Riginal	PR	47	DoD
2§ 2×10^7^/m^2^	204	White	1	IV M1c	mCRC	No	Modified	PD	2.5	DoD
	205	White	1	IV M1c	mCRC	No	Modified	PD	14	DoD
	206¶	White	1	IV M1c	mMel-MT	N/A	Modified	N/A	N/A	N/A
	207	White	0	IV M1a	mMel-WT	No	Modified	PD	29	DoD
3§ 1×10^8^/m^2^	305	White	0	IV M1	sarcoma	No	Modified	SD	23	DoD
	306	White	1	IV M1c	mCRC	No	Modified	PD	1.5	DoD
	307	White	1	IV M1c	mCRC	No	Modified	PD	2	DoD

Patients who were screened, eligible, enrolled and had CAR-T cell manufacturing performed. Staging for each cancer type was performed using the American Joint Committee on Cancer (AJCC) eighth edition.

*Had lymphodepletion chemotherapy using fludarabine and cyclophosphamide.

†Manufactured GD2-iCAR-PBT not administered because of commensal bacteria contamination, and later re-enrolled as patient 304.

‡post-GD2-CAR-T cell therapies.

§TGA-mandated cell dose re-escalation after change to modified manufacturing protocol.

¶COVID-19 travel restrictions prevented GD2-CAR-PBT administration at study site.

BRAF/MEKi, dabrafenib and trametinib combination kinase inhibitor therapy; CAR, chimeric antigen receptor; DoD, dead of disease; ECOG, Eastern Cooperative Oncology Group; mCRC, metastatic colorectal cancer; mMel-MT, metastatic melanoma with V600 BRAF mutation; mMel-WT, metastatic melanoma without BRAF mutation; NED, no evident disease; PD, progressive disease; PR, partial response; RECIST, Response Evaluation Criteria in Solid Tumors; SD, stable disease.

### Feasibility

A coprimary objective of this study was to determine the feasibility of preparing GD2-iCAR-PBT products for administration to patients with GD2-positive solid cancers. Of the 14 consented patients, 14 had CAR-T cells manufactured; 13 achieved the predefined batch release criteria (online supplemental table S1); and 12 products were administered (online supplemental figure S1). An acceptable feasibility (defined as administration of ≥80% of prepared T-cell products) was achieved with 93% of prepared products administered.

**Figure 2:**
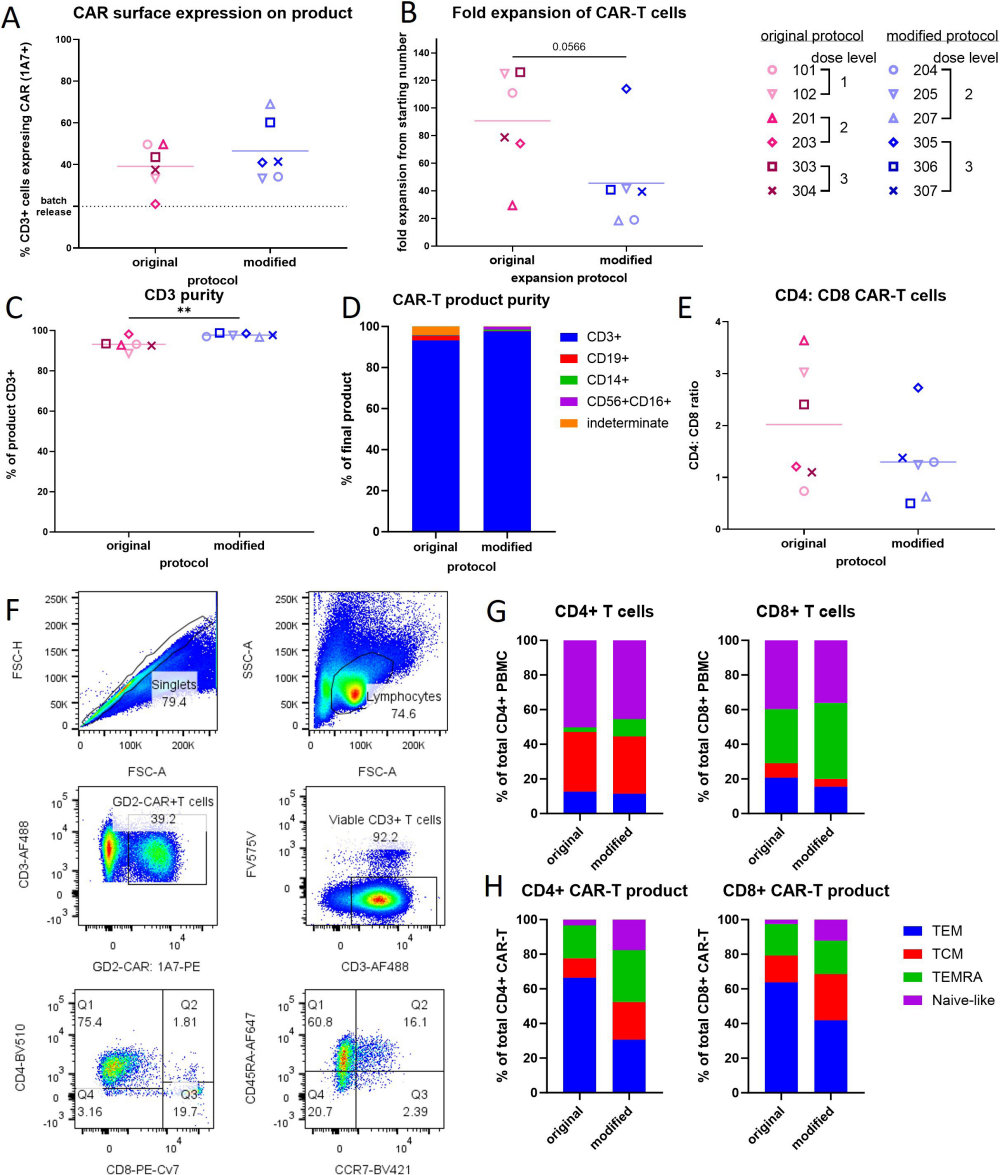
Autologous GD2-specific chimeric antigen receptor (CAR) T cell products manufactured using the original protocol (pink symbols) or modified protocol (blue symbols). Quality control assessments for CAR-T cell products: (A) transduction efficiency as determined by surface CAR expression at day 7 of culture, detected using the 1A7 anti-idiotypic antibody. (B) Fold-expansion during product culture period of 8–12 days. (C) CD3 purity at the end of the expansion period. (D) Purity of the CAR-T cell product at end of the expansion period. (E) Ratio of CD4+to CD8+ CAR T cells at end of the expansion period. (F) Flow cytometry gating strategy and representative plots for patient 304 (original manufacturing protocol) showing effector memory (TEM); central memory (TCM); TEMRA; T-naïve-like phenotype. (G) Phenotype of CD4+ and CD8+T cells from patient peripheral blood used for manufacturing and (H) the final GD2-CAR-T cell product. CAR-T cell product from the original manufacturing protocol had significantly more TEM T cells (see online supplemental figure S2). Significance was assessed by unpaired t-test, *p<0.05, **p<0.01, ***p<0.001, GraphPad Prism V.10.1.1.

CAR expression on T cell products ranged from 21% to 69% with no difference between the two manufacturing protocols (figure 2A). CAR expression level per cell, as measured by Mean Fluorescence Intensity, was also equivalent (online supplemental figure S2B). The original manufacturing protocol produced a higher fold-expansion over 9–12 days of culture compared with the modified protocol (mean of 90-fold for original vs 45-fold for modified) likely because of differences in activation reagents (plate bound anti-CD3 and CD-28 vs TransACT nanomatrix activation reagent) and culture media (fetal-calf serum supplemented media vs TEXMACS minimal serum-free media) used between manufacturing protocols (figure 2B). CD3 purity was higher in the modified protocol (mean of 93% for original vs 98% for modified) and was expected because of preculture magnetic bead selection of CD4 and CD8 T cells, and the CD4:CD8 ratio in individual products ranged from 0.7:1 to 3.6:1 (figure 2C–E). Although the memory phenotype of the starting T-cell population did not significantly differ with either protocol, the modified manufacturing protocol resulted in decreased proportions among CD8+and CD4+ CAR T cells of an effector memory phenotype (CD45RA- CCR7-) in favor of central (CD45RA- CCR7+), naïve-like (CD45RA+CCR7+) and TEMRA (CD45RA+CCR7−) phenotypes (figure 2F–H and online supplemental figure S2C,D). We have previously reported that the modified manufacturing protocol promoted a naïve-like phenotype that included expression of T stem-central memory markers: CD45RA+CCR7+ CD95+ CD45RO±when compared with the original protocol for matched donors.^23^

### Safety

The study’s other coprimary objective was to determine the safety profile and DLTs of administration of the GD2-iCAR-PBT product. No DLTs were observed, and the maximum tolerated dose was not determined. Of the 12 patients enrolled in the trial, 10 (83%) had at least one treatment-emergent AEs. Of these AEs, none was of a severity higher than grade 2. Most common were rash (50%), fever (33%), diarrhea (33%) and anorexia (33%) (online supplemental table S2). Patient 201 experienced grade 1–2 anorexia, nausea, vomiting, and diarrhea related to lymphodepletion chemotherapy. Patient 205 had grade 2 nausea related to commencement of cytotoxic chemotherapy for his metastatic colorectal cancer during the 6-week postinfusion evaluation period. In patient 305, a grade 1 fever, which occurred <48 hours post-CAR-T cell infusion, was attributed to CAR-T cell therapy as grade 1 cytokine release syndrome in the absence of alternative explanations. Five of the 12 patients, who received concurrent dabrafenib and trametinib as standard treatment for their V600 BRAF-mutant metastatic melanoma, experienced 59% of the low-grade AEs. These AEs were consistent with the known mild dabrafenib-associated and trametinib-associated constitutional, skin, gastrointestinal and arthritic symptoms. There was no evidence of neurotoxicity after the GD2-iCAR-PBT infusion, even in patients who had high disease burden or Central Nervous System (CNS) involvement. In the latter case, 2 of 12 patients, patients 101 and 304, had melanoma brain metastases (M1d disease).

Acknowledged longer-term risks of CAR-T cell therapy include the generation of replication competent retrovirus (RCR) with the potential to induce oncogenic transformation. Accordingly, during long-term follow-up, we tested patient blood samples by qPCR for retroviral envelope protein (GALV env) to indicate recombination event(s) that may enable RCR, and by flow cytometry for TCR diversity to detect emerging oligoclonal expansion that may indicate oncogenic transformation. RCR was never detected in the CAR-T cell product or in any peripheral blood sample of any patient collected after the GD2-iCAR-PBT infusion (online supplemental table S3). Although distinct TCR Vβ usage was observed between CAR-T cells in the infused product and endogenous circulating T cells just before the CAR-T cell infusion, no significant deviations or outgrowths in specific clones were identified postinfusion (online supplemental figure S3). This result indicated that clonal expansion of a transformed CAR-T cell had not occurred.

### Tumor response

Documenting an assessment of tumor response was a secondary trial objective and was determined by CT scans at or about day 42 using Response Evaluation Criteria in Solid Tumors (RECIST) V.1.1. Patients receiving BRAF and MEK inhibition concurrent with GD2-CAR-PBT infusion (5 of 12 patients) uniformly achieved a partial response, which may be expected from the combination BRAF/MEK inhibitor treatment alone.^31^ Of patients receiving GD2-iCAR-PBT alone (7 of 12 patients), 2 had transient stabilization of their previously progressing disease, and 5 had progressive disease (table 1). As patient 305 had evidence of stable disease and had not experienced DLTs, she was eligible to receive a second infusion of GD2-iCAR PBT, which she received on day 49 after the first infusion.

### Peripheral expansion and persistence

**Figure 3:**
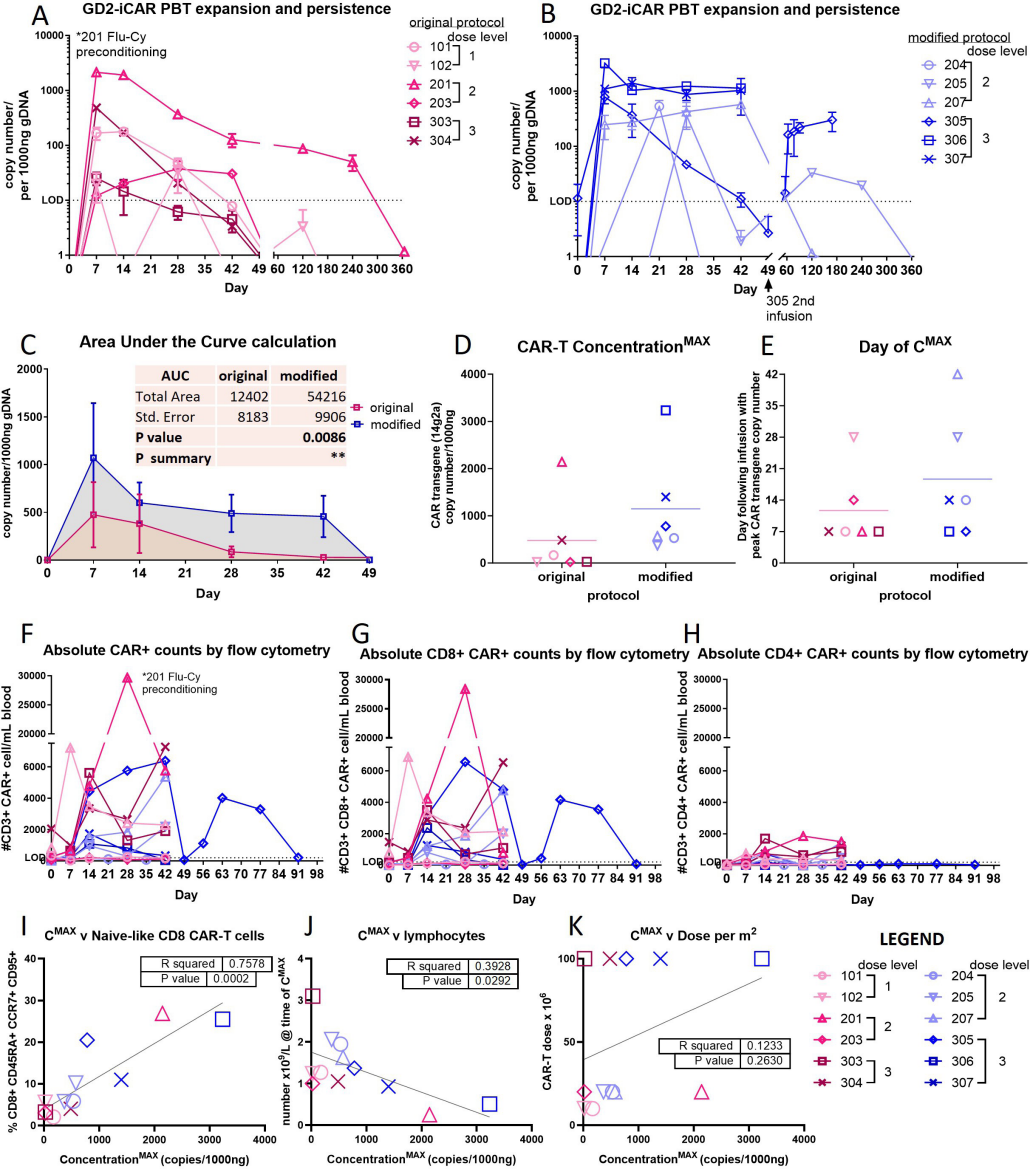
Biokinetics of GD2-iCAR-PBT according to CAR-T cell manufacturing method. Peripheral expansion and persistence were determined by quantitative PCR for CAR transgene with a Taqman probe designed for the 14g2a scFv DNA sequence, using genomic DNA isolated from whole blood: (A) Original manufacturing protocol; (B) Modified manufacturing protocol. (C) Area under the curve (AUC) calculation. (D) Peak expansion (C^MAX^) as determined by copy number per 1000 ng genomic DNA. (E) Day of peak expansion following infusion of CAR-T cell product. Significance was assessed by unpaired t-test, or Pearson correlation analysis on GraphPad Prism V.10.1.1. Flow cytometry for CAR transgene was performed using the 1A7 anti-idiotypic antibody staining of isolated peripheral blood mononuclear cells in TruCount tubes. Absolute counts of (F) CD3+CAR+ cells (G) CD3+CD4+ CAR+ cells, and (H) CD3+CD8+ CAR+ cells per mL blood. Correlation analysis between maximum expansion of CAR-T cells and (I) Naïve-like T cells in the product; (J) Absolute lymphocyte number at the time of maximum expansion; (K) Dose level. Original manufacturing protocol (pink symbols); Modified manufacturing protocol (blue symbols). CAR, chimeric antigen receptor. AUC calculation *p <0.05, **p<0.01, ***p<0.001, GraphPad Prism V.10.1.1.

A determination of CAR-T cell engraftment, expansion and persistence was made using quantitative real-time DNA PCR (qPCR) to detect in peripheral blood samples the transgene encoding the 14g2a scFv (figure 3). By this measure, all patients had detectable CAR-T cells at one or more time points postinfusion, and for half of patients, peak expansion was observed at day 7 (6 of 12 patients) (figure 3A,B). Evidence of CAR-T cell persistence waned rapidly during the 42-day DLT evaluation window, with the CAR-T transgene detectable in 5 of 12 patients after day 42. Patients treated with CAR-T cells manufactured using the modified protocol showed altered biokinetics, with an increase in the levels of circulating CAR-T cells as shown by the significantly higher area under the curve (AUC mean 12 402 vs 54,216; p=0.0086) (figure 3C). Mean peak expansion (C^MAX^: 482 vs 1177 transgene copies/ug, figure 3D) and day of peak expansion (day 11 vs day 19, figure 3E) were not significantly different among the patients who received CAR-T cells made using original or modified protocols.

In addition, flow cytometry was performed using the anti-idiotypic 1A7 antibody to detect circulating T cells with surface expression of the CAR. Although this assay had lower specificity and sensitivity, and thus a higher limit of detection than qPCR (figure 3F), it did allow immune phenotyping of circulating CAR-T cells, including quantification of CD4+ and CD8+ subsets (figure 3G,H). This analysis revealed that most circulating CAR+cells were CD8+. Although the kinetics of CAR-T cell expansion detected by flow cytometry was like that evident by qPCR, the day of peak expansion differed in some cases. In patient 201, for example, the CAR transgene peaked between days 7 and 14 but CAR surface expression peaked on day 28. This result may reflect the variable surface expression of the CAR molecule, which is influenced both by the location of retroviral insertion site(s) and T-cell activation status. In T cells transduced with gamma retrovirus, highly activated T cells are expected to be more transcriptionally active with subsequently higher expression of the retrovirally introduced transgene.^32^ Interestingly, the proportion of naïve-like CD8 T cells in the infused CAR-T cell product showed a significant positive correlation with post-infusion CAR-T cell expansion (figure 3I). Blood lymphocyte counts at the time of peak expansion showed a negative correlation with CAR-T cell expansion (figure 3J). The CAR-T cell dose level did not show an evident relationship (figure 3K, full analysis of correlations online supplemental figure S2G).

To evaluate whether CAR-T cell persistence was affected by the patient mounting an anti-CAR immune response we, determined serum human-anti-mouse antibody (HAMA) levels pre-CAR-T and post-CAR-T cell infusion (online supplemental figure S4). These data revealed that only 3 of 12 patients (patients 201, 203, and 305) mounted a HAMA response above the day 0 baseline level after the CAR-T cell infusion. Of these patients, only one (patient 201) developed high level HAMA (>10 ng/mL) and this patient had elevated HAMA at baseline. Although HAMA may indicate anti-CAR immunity, it does not directly assess cellular responses to the CAR transgene, which may still contribute to the limited persistence observed. We have previously assessed ex vivo cellular immune responses of patient peripheral blood mononuclear cell (PBMC) to irradiated CAR-T product for a subset of trial patients and did not observe significant reactivity.^29^

### Blood biomarkers of immune response

**Figure 4:**
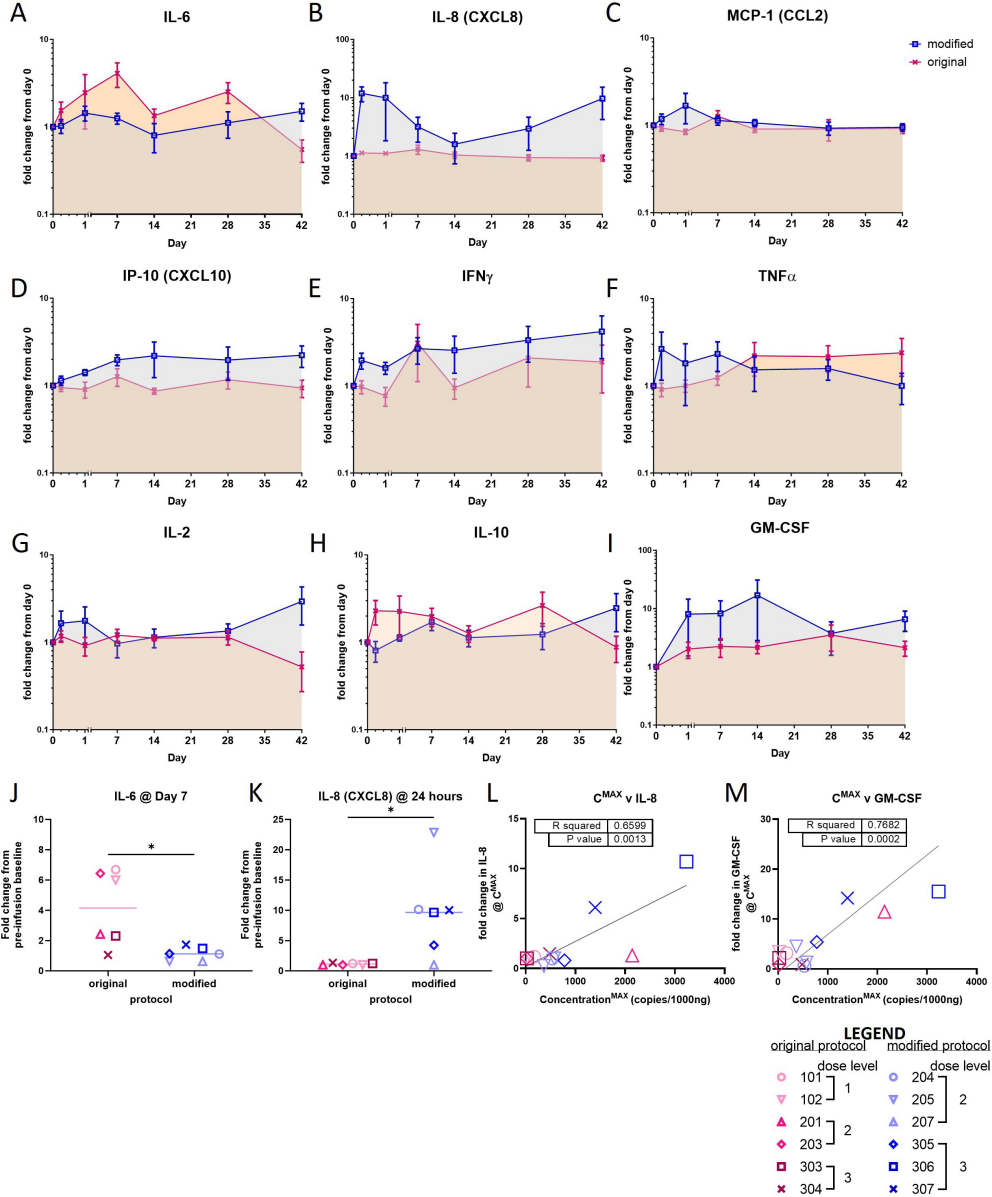
Summary of fold-change from day 0 baseline serum samples in serum cytokines after individual GD2-iCAR-PBT infusions according to CAR-T cell manufacturing method. (A–I) Fold-change in serum cytokines IL-6, IL-8, MCP-1, IP-10, IFNγ, TNFα, IL-2, IL-10, GM-CSF by cytometric bead array from the. (J) IL-6 on day 7 postinfusion and (K) IL-8 at 24 hours postinfusion; unpaired t-test, p=0.019 and p=0.015, respectively. Pearson correlation analysis between peak expansion (C^MAX^) and maximum fold-change in (L) IL-8 (M) GM-CSF. Original manufacturing protocol (pink symbols); modified manufacturing protocol (blue symbols). The following cytokines were also assessed TGFβ, IL-4, IL-12p70, IL-17A but did not show consistent changes from baseline. Patient’s individual cytokine profiles are provided in online supplemental figure S5 and absolute quantification (pg/mL) with statistical analysis is provided in online supplemental figure S6. Significance was assessed by unpaired t-test p*<0.05, **p<0.01, ***p<0.001, GraphPad Prism V.10.1.1.CAR, chimeric antigen receptor;

We also assayed other peripheral biomarkers of response via serum cytokine analysis. In patient sera, significant increases from baseline (day 0, preinfusion) were observed for IL-6 (7 of 12 patients), IL-8 (6 of 12 patients), IP-10 (CXCL10) (8 of 12 patients), MCP-1 (CCL2) (4 of 12 patients), IFN-y (5 of 12 patients), TNFα (4 of 12 patients), and GM-CSF (4 of 12 patients). See figure 4A–I for a summary of fold-change by manufacturing protocol and online supplemental figure S4 for individual cytokine profiles and online supplemental figure S5 for raw data (pg/mL) with statistical analysis. When considered by manufacturing protocol, there was a significant increase in IL-6 at day 7 for patients treated with cells manufactured under the original protocol (figure 4J), and a significant increase in IL-8 at 24 hours for patients treated with cells manufactured under the modified protocol (figure 4K). We observed postinfusion increases or decreases in serum levels of IL-2, IL-1β, IL-10 and IL-4 for some individual patients but not in most patients. Free-active TGFβ, IL-12p70, IL-17A did not change significantly from baseline (data not shown). The proinflammatory cytokines, IL-8 and GM-CSF, showed positive correlations with CAR-T cell expansion (figure 4L,M). Furthermore, the proinflammatory chemokine, MCP-1 (CCL2), together with the pan-immune-inflammation value (PIV) also showed modest positive correlations with CAR-T cell expansion (online supplemental figure S2E,F).

To specifically investigate effects on the myeloid compartment, which have been documented in numerous other trials, particularly those employing a GD2-specific CAR-T product,^11 16 33^ we employed a high-parameter flow cytometry panel designed to evaluate different circulating myeloid populations including MDSC. This revealed that most patients (six of the seven evaluated patients) had increases in either non-classical monocytes or CD14+MDSC (M-MDSC) or CD15+MDSC (PMN-MDSC) between days 7 and 14 after the CAR-T cell infusion (online supplemental figure S7). Although the correlation analysis uncovered a trend toward an inverse relationship between MDSC and CAR-T cell expansion, the low sample number limits this analysis (online supplemental figure S2G)

### Circulating CAR-T cell phenotype postinfusion

**Figure 5:**
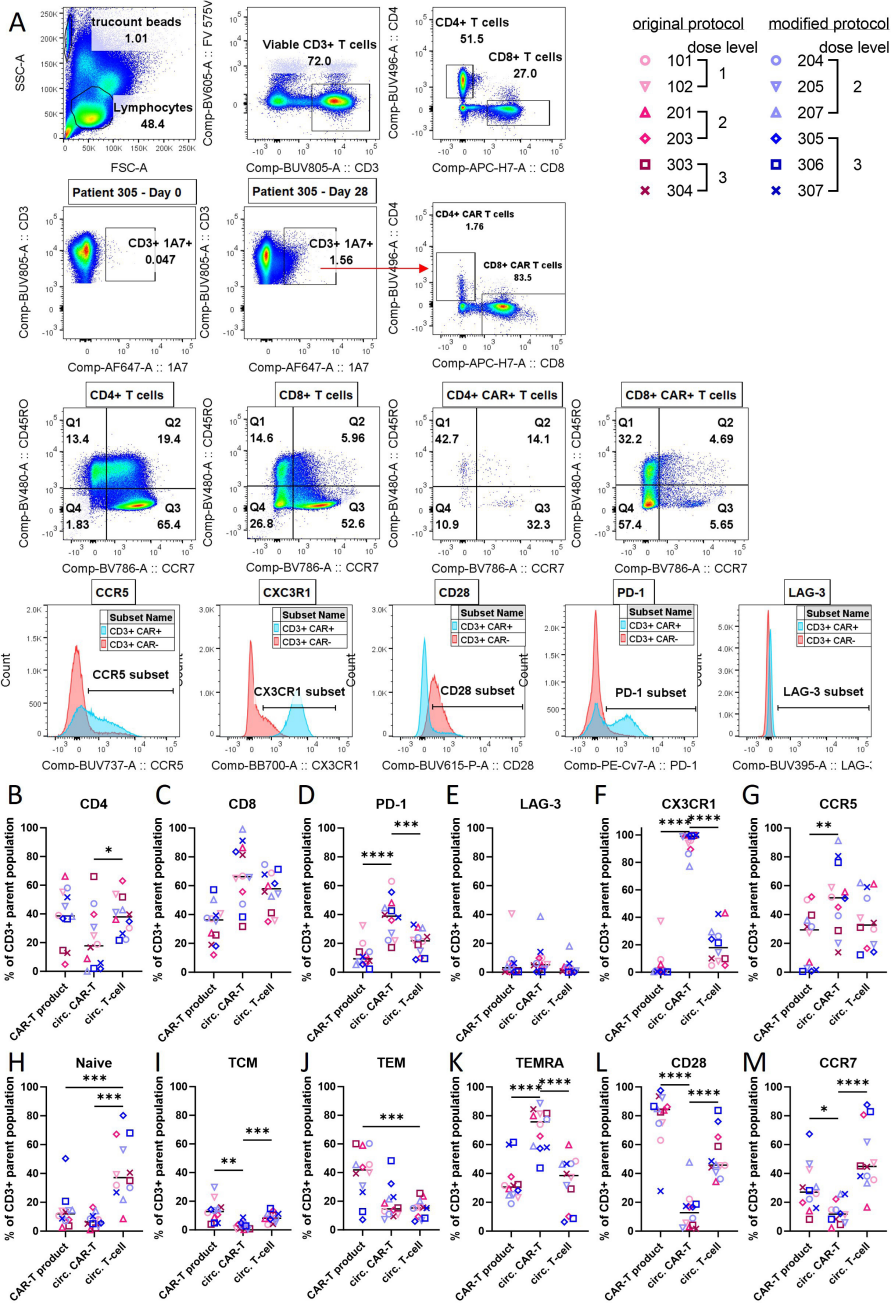
Phenotype of circulating CAR-positive T cells compared with the preinfusion GD2-iCAR-PBT product, and endogenous circulating T cells (CAR-negative) (A) Gating strategy and representative plots for patient 305 at day 28 postinfusion. A representative plot of CD3 vs CAR (1A7 anti-idiotypic antibody) from day 0 is provided to show background staining of the 1A7 antibody. Percentage of viable CD3+CAR+ T cells (for the infusion product and circulating CAR-T cell populations) or CD3+CAR T cells (for the circulating endogenous T cell population) that are (B) CD4+, (C) CD8+, (D) PD-1+, (E) LAG-3+, (F) CX3CR1+, (G) CCR5+,H) Naïve (CCR7+CD45RO−), (I) TCM (CCR7+, CD45RO+), (J) TEM (CCR7−, CD45RO+), (K) TEMRA (CCR7−, CD45RO−),L) CD28+, (M) CCR7+. Significance was determined by one-way ANOVA with Dunnett’s multiple comparisons test, *p<0.05, **p<0.01, ***p<0.001, ****p<0.0001, GraphPad Prism V.10.1.1. Further representative plots for patient 201 are also provided in online supplemental figure S8. ANOVA, analysis of variance; CAR, chimeric antigen receptor.

A number of CAR-T cells circulating at the peak of expansion (days 7–28) were sufficient to perform further high-parameter flow cytometric analysis on banked PBMC, which enabled us to interrogate the GD2-iCAR-PBT phenotype compared with both circulating endogenous T cells and the GD2-iCAR-PBT product before infusion (figure 5A and online supplemental figure S8).

We observed a distinct phenotype for circulating CAR+T cells including a lower proportion of CD4+ compared with to CD8+ cells as noted above (figure 3G,H and figure 5B,C). Compared with endogenous T cells, circulating CAR+T cells also had significantly elevated markers associated with antigen experience and effector differentiation: PD-1 (mean 20.73% vs 37.99%, p=0.0007), CCR5 (mean 23.42% vs 51.07%, p=0.0052), CX3CR1 (mean 19.91% vs 94.92%, p<0.0001)) although they did not have high levels of LAG-3 (figure 5D–G). Circulating CAR-T cells had a predominant TEMRA phenotype compared with circulating endogenous T cells, which have a predominantly naïve phenotype (figure 5H–K). Circulating CAR+T cells also had significantly lower expression of markers associated with naïve phenotype or early effector function: CCR7 (mean 51% of endogenous T cells vs 13% of CAR-T cells, p<0.0001, CD28 (mean 79.03% of endogenous T cells vs 13.75% of CAR-T cells, p<0.0001) (figure 5L,M). Higher expression of PD-1, CCR5 and CX3CR1 suggests circulating CAR-T cells have a highly activated effector and effector memory phenotype.^34^ The striking loss of CD28 on the circulating CAR-T cells compared with the preinfusion GD2-iCAR-PBT product further supports antigen engagement following infusion, rather than the activation occurring during culture and expansion.

### CAR-T cell tumor infiltration

**Figure 6:**
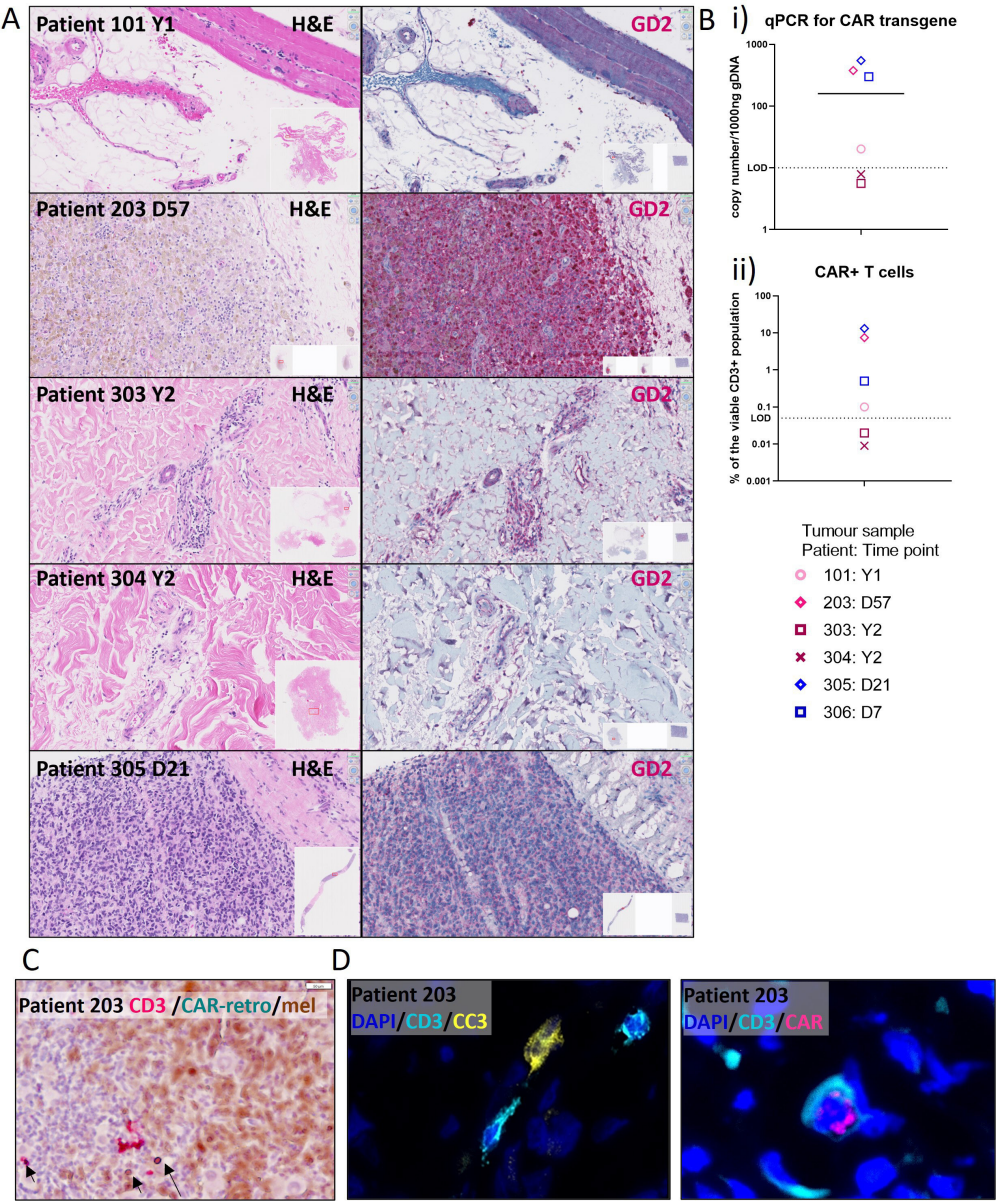
Evidence of tumor CAR-T cell infiltration. Biopsies were collected at various time points after each CAR-T cell infusion depending on patient consent and clinical circumstances (range day 21 to year 3; for full details see online supplemental table S3). (A) Tissue pieces from post-treatment biopsies were preserved in formalin with paraffin embedding (FFPE) and stained with H&E (left column) and using GD2 IHC (right column, dark red GD2 with hematoxylin counter stain). Insets show the whole scanned tissue section and region of interest in red. (B) CAR-T cells infiltrating tumor as assessed by (i) quantitative PCR for CAR transgene with a Taqman probe designed for the 14g2a scFv DNA sequence on genomic DNA derived from tumor biopsies from five patients and from an ascites sample from one patient (306); (ii) Flow cytometry using the 1A7 anti-idiotypic antibody for three patients with sufficient fresh single cell suspensions. (C) In situ hybridisation (ISC; RNAScope) was used to detect CD3 (magenta) and retroviral mRNA (CARretro; blue/green) in FFPE tissue, with melanoma cells showing brown melanin pigment. (D) Immunofluorescence analysis was performed on available fresh-frozen tissue to detect CD3, CAR (magenta), and cleaved caspase 3 (CC3; yellow) expression (representative images from the patient 203; for full tissue sections and patient 101 images see online supplemental figure S10). LOD, limit of detection; CAR, chimeric antigen receptor.

To assess CAR-T cell infiltration, an optional postinfusion tumor biopsy was done in some patients. Where sufficient and appropriate tumor material was available, fresh-frozen tissue, formalin-fixed paraffin-embedded (FFPE) tissue and tissue dissociated to a single cell suspension were all banked for future analysis. Online supplemental table S4 summarizes the post-treatment tumor biopsy material available to this study and its processing and downstream analysis. As reported by an independent pathologist, two biopsies taken during the 6-week evaluation period (from patients 203 and 305 at days 57 and 21 postinfusion, respectively) were found to have substantial tumor deposits, whereas three biopsies were found to have minimal viable tumor with areas of necrotic and fibrotic tissue and immune infiltrates (from patients 101, 303, and 304, and taken in years 1–2 post-treatment). H&E and GD2 IHC were performed on post-treatment biopsies at SA pathology (figure 6A; left column H&E, right column GD2 staining in dark red), and an automated analysis of GD2 staining intensity and tumor-infiltrating lymphocytes (TILs), which was performed by an independent pathologist, showed a trend toward decreased GD2 staining post-treatment (p=0.053) and no difference in the level of TIL (p=0.7) (online supplemental figure S9A,B).

From dissociated tissue, qPCR and flow cytometry were used to quantify the level of CAR-T cell infiltration (figure 6Bi,ii) and demonstrated CAR-T cell trafficking and infiltration above the limit of detection in three of six available patient biopsy samples. To assess CAR-T cell infiltration in situ, RNAScope was performed with probes designed to detect the mRNA originating from the GD2-CAR retrovector. Using this technique, we were likewise able to identify tumor-infiltrating GD2-CAR-T cells in three of six FFPE biopsies (figure 6C). Finally, to directly identify CAR-expressing T cells we performed multicolour immunofluorescence (IF) on the available fresh-frozen tissue samples, with costaining for cleaved caspase 3 (CC3) as an indicator of CAR-T cell-mediated cytotoxicity. Patient 203 had the most abundant staining for both CD3+T cells and CAR+T cells with CC3 staining in tumor cells adjacent to T cells (figure 6D and online supplemental figure S10).

High-parameter flow cytometry using the immune phenotyping panel described for circulating T cells was also performed for samples with sufficient material (at least 200,000 cryopreserved cells). The samples analyzed by high-parameter flow cytometry were from patient 203 and patient 305 tumor biopsies and from patient 306’s ascitic fluid (online supplemental figure S9C–L). Proportions of CD4+ and CD8+ T cells among CAR+TIL and endogenous TIL were equivalent and were mostly TEM/TEMRA (online supplemental figure S9C–F). Expression of other phenotypic markers, PD1 and LAG-3, was likewise equivalent on CAR+ and endogenous TIL (online supplemental figure S9H,I) but elevated compared with the circulating CAR+T cells described above (figure 5D,E), suggesting a more functionally suppressed phenotype. Expression of CX3CR1 was significantly elevated among CAR+T cells versus endogenous T cells in tumor tissues (mean 83% vs 10.8%, p=0.0268, online supplemental figure S9L). Evaluation of ascitic fluid during the postinfusion period revealed predominant cell types of the monocyte-macrophage lineage and T cells of which a very small proportion, were CAR+T cells, peaking at day 7 (online supplemental figure S9M–P).

## Discussion

GD2-specific CAR-T cells have been administered to over 57 patients with neuroblastoma^8 11 13 15^ and recently four patients with DMG^16^ and eight patients with glioblastoma^14^ with no evidence of on-target, off-tumor toxicity from CAR-T cells targeting GD2 on normal tissue, and with evidence of tumor responses and clinical benefit in some patients. Here, we report the safe administration of third-generation, GD2-specific autologous CAR-T cell therapy to 12 patients with metastatic GD2-positive solid cancers. Although treatment-emergent AEs were recorded in most patients, all were low grade, and no patient experienced a DLT. Our results are like those recently reported for a 13-patient trial using an identical GD2-CAR vector in patients with relapsed neuroblastoma and osteosarcoma. In that study a different manufacturing protocol with cell expansion in IL-2 was used and all patients received preconditioning chemotherapy, however, similar outcomes of variable expansion, limited persistence and no long-term tumor responses^13^ were observed as in our study.

Peripheral expansion and persistence is an important biomarker of CAR-T cell function and correlates with efficacy in CD19-CAR-T cell therapy. For example, the median value for peak expansion in the ELIANA and ENSIGN trials with Tisagenlecleucal was 44 800 copies/μg DNA for responders compared with 22,000 copies/μg DNA in non-responders.^35^ 1000 copies/100 ng has recently been used as a benchmark for “good” expansion in a GD2-CAR-therapy trial.^13^ However, there have not been enough studies of effective CAR-T cell therapy in solid cancer patients to determine biological correlates of therapeutic success. Here, we report the peripheral expansion and persistence of CAR-T cells, acknowledging that this measure does not capture CAR-T cells residing in the tumor tissue and secondary lymphoid organs. Irrespective of dose level, we found limited CAR-T cell persistence beyond 6 weeks, with a maximum expansion of 3233 copies/µg DNA for patient 305 whose CAR-T cells were manufactured using the modified protocol. Only patient 305 received a second infusion and the AUC of this second CAR-T cell expansion, as detected by qPCR for the GD2-iCAR transgene, was not significantly different from the AUC after the first infusion. As expected, patient 201, the only patient who had received prior lymphodepletion, also exhibited improved expansion.

Previously, we observed that the modified manufacturing protocol changed CAR-T cell phenotype to increase the proportion of central-memory-like, CCR7 and CD62L-expressing cells.^21^ Here, we found that the patients treated with these optimized CAR-T cell products had improved CAR-T expansion and persistence, as represented by a significantly higher AUC, despite these patients having at the time of enrolment such indications of poorer response to immunotherapy as a reduced performance status and higher PIV.^30 36^ CAR-T cell persistence in patients has been linked to the availability of antigen and antigen-presenting cell (APC) support in providing the third signal for costimulation.^37^ Although CD19-CAR-T cells have access to antigen and APCs in circulation, CAR-T cell therapies directed toward solid cancer targets do not. Therefore, our GD2-CAR-T cells may not persist because of inaccessible or unavailable GD2 target antigen or do not establish in secondary lymphoid organs where they might receive costimulatory support from APCs, or both. Activation-induced cell death is another persistence-limiting mechanism, which we identified when GD2-CAR-T cells were manufactured using the original protocol,^29^ and which is partially prevented using the modified manufacturing protocol.^21^ Finally, humoral, and cellular immune responses to the chimeric proteins induced by exposure to the CAR molecule have also been identified as a mechanism of CAR-T cell deletion.^38^ However, most of our patients did not display elevated HAMA, which serves as a marker of the immunogenic potential of the CAR.^39^

Changes in serum cytokine levels may represent systemic effects of cancer-induced inflammation, which is reflected in an elevated PIV. An elevated PIV often accompanies poor prognosis malignancies^36^ as evident in some patients enrolled to this trial. Changes in serum cytokine levels may also provide evidence of CAR-T cell-related immune reactions, which might manifest as cytokine release syndrome within 72 hours of the infusion, or later be related to systemic immune reactions following antigen engagement by CAR-T cells. Many patients had postinfusion increases in serum concentrations of the T cell-related cytokines IFNγ and TNFα, whereas IL-6 and IL-8, as markers of more general systemic inflammation, showed peaks at earlier postinfusion time points. Interestingly, the different patterns of IL-6 and IL-8 secretion depended on the type of manufacturing protocol with the original protocol resulting in higher serum IL-6 levels and, conversely, the modified protocol resulting in higher serum IL-8 levels. The pronounced effect that the original protocol has on generating effector memory T cells may help to explain the observation of relatively increased serum levels of IL-6. Several patients had postinfusion elevations of CXCL10 (IP-10), particularly patient 205 who restarted cytotoxic chemotherapy during the 6-week evaluation period because of the patient’s concern over a rising serum CEA tumor marker. IP-10 signals T cells via CXCR3 to induce T-cell infiltration of tumors and its production has indicated, for example, an antimelanoma T-cell response after PD-1 inhibitor therapy.^40^

We also noted changes in circulating myeloid cells as has been reported for other GD2-CAR-T cell therapy trials.^11 13 15^ There is an increasing evidence that subsets of suppressive myeloid cells can curtail the effectiveness of CAR-T cells, but it is less clear whether these are solely pre-existing, tumor-induced populations or are also induced in response to the CAR-T cell infusion and following target antigen engagement within the tumor.^11 13 15 16^ Our data support a CAR-T cell-mediated expansion of monocyte and MDSCs, which was observed on days 7–14 postinfusion in some patients. The postinfusion appearance in peripheral blood of different subsets of MDSC may serve as a biomarker for CAR-T cell engagement within the tumor and represent a tumor-driven mechanism for subverting T-cell effector responses.^41^ Certain cytotoxic drugs can reduce MDSC populations,^42 43^ and CAR-T engineering approaches that reprogram myeloid cells to an “immune-activation” phenotype or eliminate MDSC,^44 45^ may help to improve CAR-T cell therapy for solid cancer patients.

Determining biomarkers associated with CAR-T cell persistence and function in solid cancer patients remains an important clinical research goal. In this study, we have found that correlates of expansion included a higher proportion of naïve-like T cells in the infusion product and an increase in some inflammatory cytokines (GM-CSF, in keeping with a recent report,^13^ CCL2 and IL-8) but notably not CAR-T cell dose level. However, the low number of patients limited our ability to identify any biomarker correlates of potentially positive clinical outcomes like disease stabilization.

Of the patients with marked CAR-T cell expansion, patient 201 who had lymphodepletion, and patients 205, 305, 306, and 307, whose products were manufactured using the modified protocol, all had the highest fold increases in CCL2 and GM-CSF and, with the exception of Patient 305, also had the highest PIV. Together, these data are consistent with the hypothesis raised recently in the report of GD2-CAR-T cell therapy in patients with solid cancer,^13^ that robust CAR-T cell activity ultimately limits its own effectiveness via CAR-T cell-induced expansion of protumorigenic myeloid cells.

In summary, the CARPETS study confirmed the safety of the GD2-iCAR-PBT but also showed a lack of persistence of the CAR-T cell product in the circulation together with the induction of potentially immune suppressive myeloid cells in some patients. Despite prior expectations that, by apparently overcoming oncogene-induced intratumoral immune suppression,^24 25^ concurrent BRAF/MEK inhibitor therapy would augment adoptive cell therapies^27^ we did not discern any positive effect of BRAF/MEK inhibition (by dabrafenib and trametinib) on GD2-CAR-T cell expansion or persistence in metastatic melanoma patients. It is possible that BRAF/MEK inhibitors impairs the function of T cells as we have reported in vitro.^46^ Five patients initially treated with dabrafenib and trametinib responded as anticipated for patients receiving kinase inhibitor therapy only. Of patients receiving only GD2-iCAR-PBT without kinase inhibitor, five had progressive disease at follow-up, and two patients had disease stabilization. Disease progression was associated with the advanced treatment-resistant disease of these patients at the time of enrolment, as indicated by markers of cancer-induced inflammation and reduced performance status.

Although there was indirect and direct evidence of intratumoral CAR-T cell engagement in most patients by the detection of tumor CAR-T cell infiltration, immune phenotypic changes in circulating CAR-T cells, and postinfusion elevations of serum cytokines, these findings did not correspond to apparent CAR-T cell-related disease control. Although we do not consider the anticancer activity of this CAR-T cell product alone sufficient to warrant a phase 2 study, we note that, after the CARPETS trial began, several improvements in GD2-CAR retroviral constructs, which aim to prolong CAR-T cell persistence and enhance antitumor responses. For example, changes to the CAR molecule structure together with coexpression of the immunostimulatory cytokine, IL-15,^47 48^ or the addition of a constitutively active IL-7 receptor (NCT03635632), are being tested clinically. Recently, cancers such as gliomas with high and homogeneous GD2 expression also represent promising target diseases for GD2-CAR-T cell therapy.^3 16^ In conclusion, GD2-targeting CAR T cells remain an active and promising CAR-T cell therapy for multiple tumor indications.

## Methods

### Study design

This single-centre, open label, phase 1 trial was prospectively registered in the Australian and New Zealand Clinical Trials Registry (www.anzctr.org.au: ACTRN 12613000198729).

All patients provided written informed consent. Dose escalation employed the Bayesian dose-finding modified continual reassessment method (mCRM) to determine the safety of one intravenous injection of autologous GD2-iCAR-PBT. Dose escalation proceeded through three cell dose levels: (1) 1×10^7^/m^2^, (2) 2×10^7^/m^2^, and (3) 1×10^8^/m^2^. Patients over 18 years old were eligible for enrolment if their tumors had GD2 expression on at least 10% of tumor cells. For patients with unresectable, metastatic melanoma who had V600E/K/R/D BRAF mutant disease, oral dabrafenib (150 mg two times per day) and trametinib (2 mg once daily) kinase inhibitor therapy was commenced while their CAR-T cell product was being prepared. Administration of dabrafenib and trametinib continued as per standard of care until disease progression or intolerance of therapy. Metastatic melanoma patients whose tumors lacked a V600 BRAF mutation, and thus who were ineligible for combination kinase inhibitor therapy, could receive intravenous lymphodepletion chemotherapy comprising fludarabine 30mg/m^2^ on days −4 to –3 and −2 and cyclophosphamide 500 mg/m^2^ on days −4 and −3 before the CAR-T cell infusion on day 0. In patients not receiving dabrafenib and trametinib, a repeat CAR-T cell infusion was allowed in the absence of DLTs and with at least stable disease at the first tumor assessment.

All autologous cell production occurred in the Cellular Therapies Laboratory at SA Pathology, Adelaide, under GMP-like conditions with Clinical Trials Exemption/Clinical Trials Application scheme approval (#CTX-2014-001 V2) from the Therapeutic Goods Administration of Australia. PBMCs were collected from 280 mL peripheral venous blood of eligible patients to produce CAR-T cells. The original manufacturing protocol was adapted from the Center for Cell and Gene Therapy, Baylor College of Medicine (Houston, Texas, USA) and used for the first six treated patients.^20 28 29^ Aiming to improve CAR-T cell persistence in vivo, a modified manufacturing protocol using Miltenyi Biotec reagents was adopted for the final six treated patients^21^ with further details provided below. For the latter patients, dose escalation recommenced at the level 2 cell dose of 2×10^7^/m^2^. In all cases, after cell manufacture, CAR-T cells were cryopreserved and products were not released for later intravenous infusion unless stringent batch release quality criteria were satisfied.

All patients were monitored intensively for 24 hours postinfusion at the Royal Adelaide Hospital and then reviewed clinically with blood draws at the following time points: days 7, 14, 28, and 42 of the 6-week postinfusion DLT evaluation period. CT for tumor assessment was performed approximately 6 weeks postinfusion and subsequently as per standard of care to obtain tumor measurements using RECIST V.1.1. Subsequent clinical reviews with blood draws occurred at months 4, 8, 12 and at years 2–15 postinfusion.

Trial endpoints and evaluation criteria are defined in full in online supplemental methods.

10.1136/jitc-2023-008659.supp2 Supplementary data

### Retroviral vector transduction and CAR-T cell production

The GMP-grade SFG retroviral supernatant encoding the transgene iCasp9.2A.14g2a.OX40.CD28.zeta was obtained from Baylor College of Medicine (Houston, Texas, USA). The original manufacturing protocol was described previously.^23 29^ Briefly, PBMCs were activated with plate-bound CD3 and CD28-specific antibodies in T-cell media (45% advanced RPMI, 45% Click’s Media, 10% Fetal Bovine Serum, 1% L-Glutamine, supplemented with recombinant human (rh) IL-7 (10 ng/mL) and rhIL-15 (5 ng/mL) from 48 hours) for 72 hours before retroviral transduction and further expansion in Grex flasks (Wilson Wolff). Under the modified manufacturing protocol,^23^ CD4-positive and CD8-positive cells were magnetically selected from peripheral blood using the Clinimacs Plus with CD4 and CD8 Reagent (Miltenyi Biotec). Then, cells were activated using GMP-grade TransACT in TEXMACS medium (Miltenyi Biotec) according to the manufacturer’s instructions, and the medium was supplemented with rhIL-7 and rhIL-15 as above. Subsequently, activated T cells were retrovirally transduced and expanded as above. On the final day of expansion, cells were washed and resuspended in 45% Albumex20 (Human Serum Albumin; CSL Behring) 45% HBSS (Hank’s Balanced Salt Solution, Gibco) and 10% Cryosure DMSO (WAK-Chemie) for cryopreservation. Finally, GD2-PBT, iCAR-PBT underwent quality control testing against batch release criteria (online supplemental table S1).

### Assessment of CAR-T cell persistence

Persistence of GD2-iCAR-PBT was determined both by quantitative real-time PCR (qPCR) for GD2-iCAR transgene using the published method^1^ and flow cytometry using the anti-idiotypic mAb, 1A7 to detect the 14g2a paratope of the GD2-iCAR. Where available, dissociated tumor samples were also used for assessment of GD2-iCAR-PBT persistence by qPCR and flow cytometry, with tissue dissociation performed using the GentleMACS dissociator (Miltenyi Biotec) and human Tumor Dissociation Kit (Miltenyi Biotec).

### Serum cytokine assessment

Serum samples, which were collected from patients at day 0, post-infusion at 6 hours and on days 1, 7, 14, 28, and 42, were analyzed using a 13-plex Cytometric Bead Array (Legendplex Human Essential Immune Response Panel; Biolegend) for the presence of the following cytokines: IL-4, IL-2, CXCL10 (IP-10), IL-1β, TNF-alpha, CCL2 (MCP-1), IL-17A, IL-6, IL-10, IFN-gamma, IL-12p70, CXCL8 (IL-8), and TGF-β1. Cytokine levels (pg/mL) were normalized to baseline (day 0 preinfusion) and expressed as fold-change. Human GM-CSF was detected by ELISA (R&D systems).

### Flow cytometry for immune phenotyping, TCRvβ usage

To perform basic phenotyping of the starting PBMC population and manufactured CAR-T product, two antibody panels were used (see online supplemental methods for antibody details) and samples analyzed on the BD FACSymphony A5. FMO controls were used to determine gating for non-bimodal populations, and TruCount tubes (BD Biosciences) were used to give absolute counts/mL blood. For analysis of circulating myeloid populations, the Cytek cFluor MDSC kit was used with staining analyzed on the Cytek Northern Lights spectral flow cytometer. For TCR diversity assessment, the Beta Mark TCR Vβ Repertoire Kit (Beckman Coulter) and anti-CD3-PerCP-Cy5.5 (BD Biosciences) were used to stain the CAR-T product and PBMC collected on day 0 (preinfusion) and at months 4, 8, and 12 and then yearly for up to a total of 15 years.

### Tissue analysis by IHC and IF

To determine eligibility for the trial, GD2 IHC screening was performed in a diagnostic setting by SA (South Australia) Pathology on patient formalin-fixed, paraffin-embedded (FFPE) biopsy samples using heat-based antigen retrieval (CC1: Tris-EDTA buffer pH 7.8 at 92°C for 36 min) using the 14g2a antibody (1:40, BD biosciences) and ULTRAview red detection on the Ventana ULTRA automated system. Results were reported by independent pathologists.

Patients could consent to an optional tumor biopsy 2 weeks after the CAR-T cell infusion, and tumor biopsy material was also collected at later time points if a biopsy was indicated during a patient’s clinical care. Where sufficient material was available, biopsies were divided in three with material placed in formalin for FFPE processing, OCT for fresh-frozen tissue sectioning, and used to obtain a single cell suspension (Miltenyi Human Tumor Dissociation Kit) for flow cytometry and genomic DNA extraction. IF staining was performed on fresh-frozen sections, and the RNAScope detection of mRNA was performed on FFPE sections, as described in online supplemental methods. For preinfusion and postinfusion FFPE tumor samples, matched H&E and GD2 stains were provided by SA Pathology for digital scanning and analysis with the image processing software, QuPath, as described in online supplemental methods.

10.1136/jitc-2023-008659.supp3 Supplementary data

## Data Availability

Data are available on reasonable request. Original datasets (deidentified patient data, scanned microscopy images, flow cytometry FCS files) will be made available by the authors on reasonable request to the corresponding author (TG, RAH Cancer Clinical Trials Unit, 6E351, Royal Adelaide Hospital, Adelaide, SA 5000, E: tessa.gargett@sa.gov.au).
