# Supplementary material for: Safety and biological outcomes following a phase 1 trial of GD2-specific CAR-T cells in patients with GD2-positive metastatic melanoma and other solid cancers
Source: J Immunother Cancer. 2024 May 15;12(5):e008659. doi: 10.1136/jitc-2023-008659 (PMC11097842; doi:10.1136/jitc-2023-008659)
Supplement: Supplementary data [file jitc-2023-008659supp001.pdf]

Supplementary Figures.

Table S1: Batch release criteria
Figure S1: CONSORT flow diagram
Figure S2: CAR-T product phenotype, patient Pan-inflammation Value at baseline and correlation analysis for biomarkers and expansion
Table S2: Patient Safety Data following GD2-iCAR-PBT infusion
Table S3: Replication Competent Retrovirus (RCR) table
Figure S3: T Cell Receptor (TCR) diversity
Figure S4: Serum cytokine and biomarker individual profiles
Figure S5: Serum cytokine graphs with raw values
Figure S6: Myeloid Derived Suppressor Cell (MDSC) analysis
Figure S7: Human Anti-Mouse Antibody (HAMA) analysis
Figure S8: Patient 201 representative flow cytometry plots for PBMC
Table S4: Tumour samples collected post-treatment
Figure S9: Analysis of post-treatment biopsies by IHC, qPCR and flow cytometry
Figure S10: Fresh-frozen tissue section immunofluorescence

Patient Study Number	Cell Dose (dose level x BSA)	Patient Weight(kg)	Total Cells Required	Final Cell Yield	Viability Post Thaw(%)	Meet Viability Batch Release Criteria	Total Endotoxin/ Cell Dose	Endotoxin/kg	Meet Endotoxin Batch Release Criteria	Aerobic Peds Testing	Meet Contamination Testing Batch Release Criteria	Anaerobic Lytic Testing	Meet Contamination Testing Batch Release Criteria
Release Specification					≥70%			<5EU/kg/hour		Negative @10 days		Negative @10 days	
101	1.43E+07	45.9	1.53E+08	8.00E+08	94.12	TRUE	2.1	0.04	TRUE	Negative	TRUE	Negative	TRUE
102	2.00E+07	85.95	1.71E+08	9.00E+08	92.31	TRUE	2.4	0.03	TRUE	Negative	TRUE	Negative	TRUE
201	4.00E+07	120.7	2.76E+08	4.24E+08	82.56	TRUE	1.38	0.01	TRUE	Negative	TRUE	Negative	TRUE
202	2.68E+07	65.75	2.28E+08	2.29E+08	90.91	TRUE	2.4	0.036	TRUE	Positive	FALSE	Negative	TRUE
203	4.00E+07	90	3.37E+08	1.07E+09	83.33	TRUE	9.15	0.1	TRUE	Negative	TRUE	Negative	TRUE
303	2.00E+08	119.3	9.40E+08	1.81E+09	91.11	TRUE	0.6	0.005	TRUE	Negative	TRUE	Negative	TRUE
304	1.81E+08	63.5	8.39E+08	1.13E+09	95.37	TRUE	5	0.07	TRUE	Negative	TRUE	Negative	TRUE
303	4.00E+07	120	3.24E+08	3.30E+08	88.37	TRUE	9	0.075	TRUE	Negative	TRUE	Negative	TRUE
204	3.74E+07	74.85	2.00E+08	2.05E+08	86.36	TRUE	0.2	0.002	TRUE	Negative	TRUE	Negative	TRUE
205	3.88E+07	77.8	2.84E+08	5.00E+08	89.80	TRUE	0.2	0.0025	TRUE	Negative	TRUE	Negative	TRUE
207	3.92E+07	81	3.34E+08	2.65E+08	72.00	TRUE	0.2	0.0025	TRUE	Negative	TRUE	Negative	TRUE
305	2.00E+08	102	9.60E+08	1.30E+09	91.25	TRUE	2	0.019	TRUE	Negative	TRUE	Negative	TRUE
306	2.00E+08	128	1.06E+09	4.90E+08	71.11	TRUE	9.6	0.075	TRUE	Negative	TRUE	Negative	TRUE
307	1.80E+08	68.8	9.00E+08	4.40E+08	69.12	TRUE	9.6	0.139	TRUE	Negative	TRUE	Negative	TRUE

Patient Study Number	% Specific Lysis GD2CAR:LAN-1 20:1	Meet CTL Batch Release Criteria	% lymphocytes 1A7+	Meet Transduction Efficiency Batch Release Criteria	% Viable 1A7+ Lymphocytes Remaining after AP1903	Meet AP1903 Batch Release Criteria	TestGene CP GD2CARPBT	Meet RTPCR Batch Release Criteria
Release Specification	≥20%		≥20%		≤10% viable 1A7+ cells remaining		CP=0 for GALV env test gene	
101	25	TRUE	49.7	TRUE	8.88	TRUE	0	TRUE
102	40	TRUE	33.35	TRUE	8.71	TRUE	0	TRUE
201	25	TRUE	49.75	TRUE	8.26	TRUE	0	TRUE
202	29	TRUE	44.9	TRUE	0.00	TRUE	0	TRUE
203	20	TRUE	21.1	TRUE	5.80	TRUE	0	TRUE
303	22	TRUE	43.6	TRUE	5.65	TRUE	0	TRUE
304	30	TRUE	37.55	TRUE	7.00	TRUE	0	TRUE
303	94	TRUE	43.2	TRUE	8.49	TRUE	0	TRUE
204	87	TRUE	28	TRUE	6.60	TRUE	0	TRUE
205	96	TRUE	51	TRUE	5.77	TRUE	0	TRUE
207	98	TRUE	33.4	TRUE	4.98	TRUE	0	TRUE
305	100	TRUE	69	TRUE	7.32	TRUE	0	TRUE
306	100	TRUE	60.2	TRUE	7.46	TRUE	0	TRUE
307	100	TRUE	41.4	TRUE	7.73	TRUE	0	TRUE

Table S1. Batch release criteria for the manufacturing process for each CARPETS patient (n=14). Specifications for batch release: Required yield is calculated based on dose level adjusted for Body Surface Area (BSA) x3 and additional cells required for quality control testing and archive . Viability >70% by trypan blue exclusion; Endotoxin <5EU/kg/min. Microbial contamination negative (aerobic and anerobic BacTec). Cytotoxicity >20% killing of GD2+ target (originally determined by Cr51 release for the original manufacturing protocol (Patients 101, 102, 201, 202, 203, 303 and 304) and later by impedance based real-time cytotoxicity assay for the modified manufacturing protocol (Patients 204, 205, 207, 305, 306, and 307). Transduction efficiency >20% positive for CAR surface expression by 1A7 flow cytometry. Suicide gene activity less than 10% residual viable CAR+ T cells at 48 hours. Replication Competent Retrovirus assay - no GALV envelope DNA detected by real-time PCR (recorded as threshold crossing point (CP) of zero). NB: Patient 202 CAR-T product not released because of failure of batch release criteria and this individual was re-enrolled as Patient 304. Patient 303 was enrolled twice, once on each protocol.; Patient 206 CAR-T cell product not released because COVID travel restrictions prevented the patient's continuing participation in the trial.

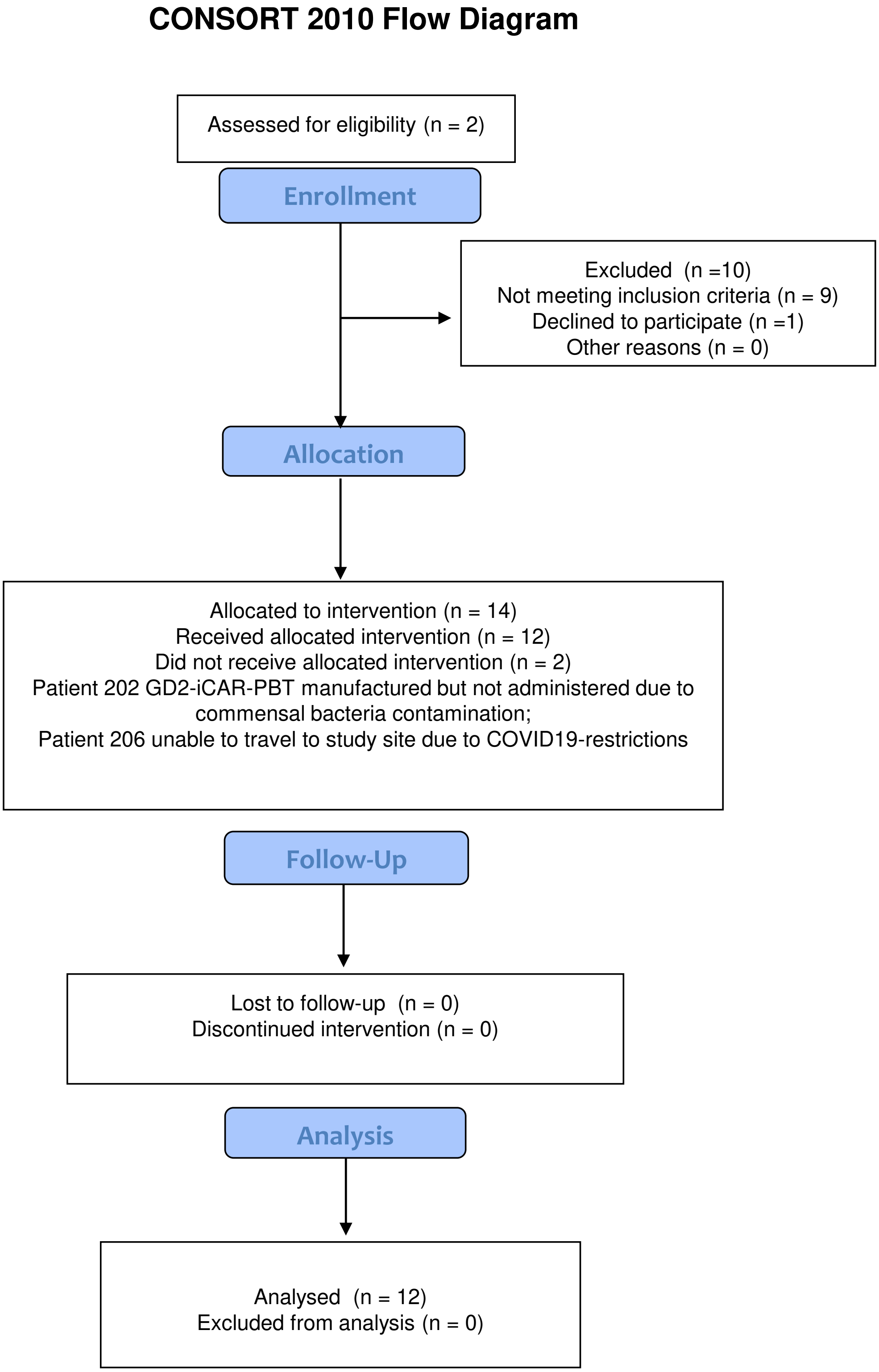

Figure S1. CONSORT flow diagram (2010 version) for CARPETS trial.

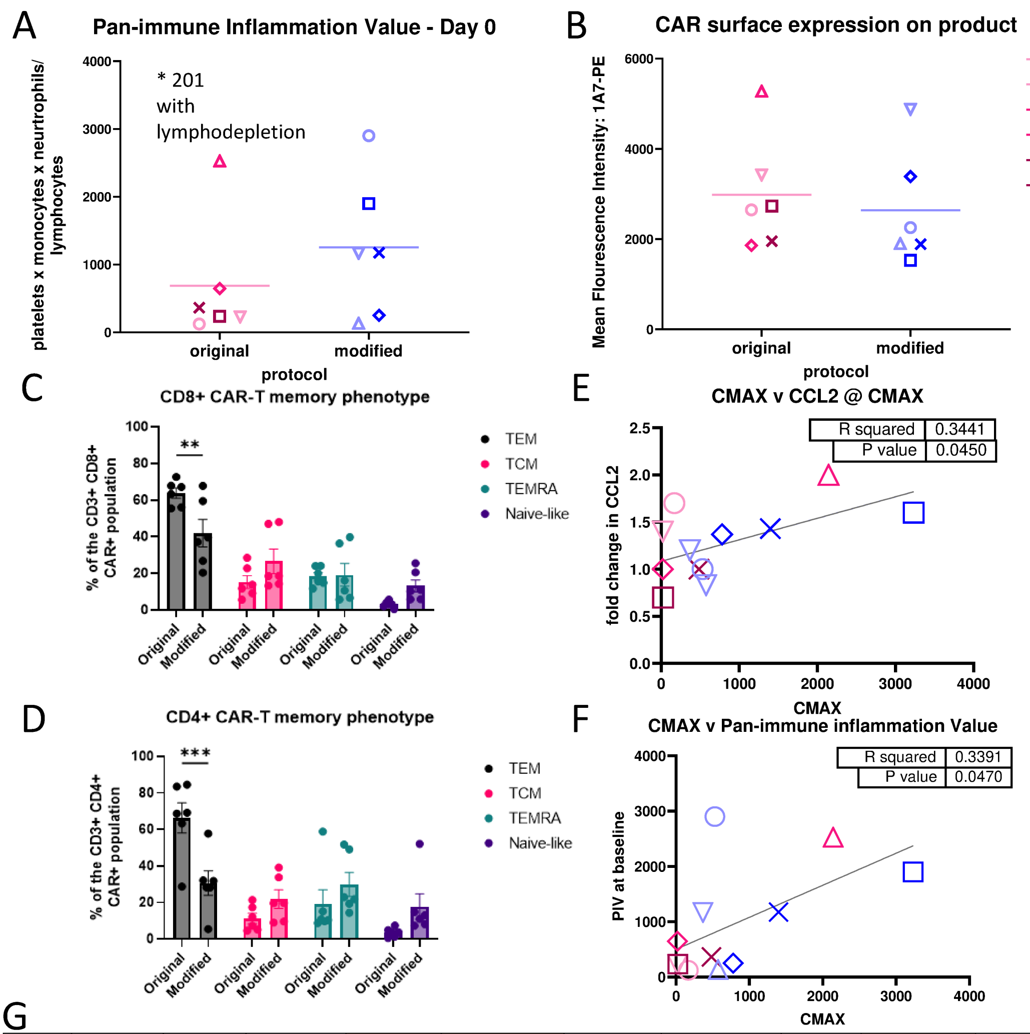

G

max IL-2	IL-2 @ CMAX	max IL-10	IL-10 @ CMAX	max GM-CSF	GM-CSF @ CMAX	max CXCL10	CXCL10 @ CMAX	max CCL2	CCL2 @ CMAX	max IL-6	IL-6 @ CMAX	max IL-8	IL-8 @ CMAX
0.01	0.0006	0.18	0.01	0.76	0.73	0.03	0.003	0.04	0.34	0.02	0.02	0.03	0.65
0.007	0.005	0.05	0.02	0.65	0.36	0.0003	0.03	0.15	0.3	0.06	0.05	0.07	0.34
0.73	0.93	0.15	0.67	0.0002	0.004	0.57	0.85	0.52	0.045	0.59	0.59	0.5	0.0013
0.78	0.81	0.48	0.65	0.001	0.03	0.95	0.54	0.2	0.06	0.43	0.45	0.37	0.043

CD4 TCM product	CD4 TCM PBMC	CD8 naive product	CD8 naïve PBMC	CD4 naive product	CD4 naive PBMC
0.06	0.06	0.75	0.48	0.004	0.001
0.009	0.04	0.32	0.16	0.08	0.02
0.7	0.58	0.002	0.1	0.83	0.89
0.75	0.54	0.05	0.19	0.35	0.65

MDSC @ day 0	MDSC @ CMAX	monocytes @ day 0	monocyes @ CMAX	lymphocytes @ day 0	lymphocyes @ CMAX	PIV @ day 0	PIV @ CMAX	Dose Level	GD2 expression
0.5	0.09	0.1954	0.1149	0.3	0.3928	0.33	0.2324	0.12	0.036
0.47	0.24	0.001	0.001	0.28	0.3564	0.2	0.21	0.07	0.021
0.07	0.5	0.1503	0.2811	0.06	0.0292	0.047	0.1155	0.26	0.55
0.08	0.25	0.9	0.89	0.07	0.0404	0.14	0.12	0.4	0.65

Figure S2. Patient and product characteristics with possible effects on post-infusion kinetics of CAR-T cells. **A**) The Pan-immune Inflammation Value (PIV) was determined using the formula [(neutrophils x monocytes x platelets)/ lymphocytes] for each patient at the time of the CAR-T cell infusion (day 0). **B**) Mean Fluorescence Intensity of CAR expression in the CAR-T cell products. Memory phenotype of the **C)** CD8+ and **D)** CD4+ CAR-T cells in the product as determined by flow cytometry. Memory subsets were defined as: Effector memory (TEM; CD45RA- CCR7-) T cells; Central memory (TCM; CD45RA- CCR7+) T cells; TEMRA T cells (CD45RA+ CCR7-) and Naïve-like (CD45RA+ CCR7+) T cells. Significance was assessed by unpaired t test, GraphPad Prism 10.1.1 Original manufacturing protocol (pink symbols); Modified manufacturing protocol (blue symbols). Pearson correlation analysis of maximum CAR-T expansion (C^{MAX}) and **E)** CCL2 at time of CMAX **F)** Pan-Immune Inflammation Value (PIV) at baseline **G**) Tabular summary of unbiased Pearson correlation analysis of biomarkers vs C^{MAX} or CAR-T cell area-under-the-curve (AUC).

Cohort by Cell Dose Level	1	1	2	2	3	3	$2^{\#}$	$2^{\#}$	$2^{\#}$	$3^{\#}$	$3^{\#}$	$3^{\#}$
**Patient ID**	101	102	201	203	303	304	204	205	207	305	306	307
**Constitutional Symptoms**												
**Fever**		2			1					1*		1
**Fatigue**		1										
**Pain**			1	2								
**Lethargy**		1										
**Malaise**						1						
**Respiratory Symptoms**												
**Cough**		1										
**Gastrointestinal Symptoms**												
**Nausea**			2		1							
**Vomiting**			2									
**Diarrhea**		1	1		1					1		
**Constipation**				1		1						
**Taste alteration**					1							
**Dyspepsia**												
**Anorexia**			1	1				2		1		
**Dermatological and Joint Symptoms**												
**Pruritus**			1							1		
**Rash**	1	2		1	2	1				1		
**Arthritis**				2								
**Haematological Abnormalities**												
**Anaemia**							1					1
**Leukopenia**												
**Lymphopenia**	1	1										
**Neutropenia**												
**Thrombocytopenia**												

Table S2. Patient Safety Data following GD2-iCAR-PBT infusion. Grading according to the NCI Common Terminology Criteria for Adverse Events version 4.0. Adverse events were included if they were assessed to be possibly, probably, or definitely related to treatment. No distinction was made between AE relationship to dabrafenib/trametinib or the GD2-iCAR-PBT infusion. # TGA-mandated cell dose re-escalation after change to modified manufacturing protocol; * fever related to grade 1 cytokine release syndrome.

Patient Study Number	Day Of RTPCR Exp	Test Gene CP in GD2CARPBT	Concentration Of Test Gene In GD2CARPBT	RunValid
101	Pre-Administration	0	0	TRUE
101	Month 4	0	0	TRUE
101	Month 8	0	0	TRUE
101	Month 12	0	0	TRUE
101	Year 2	0	0	TRUE
101	Year 3	0	0	TRUE
101	Year 4	0	0	TRUE
101	Year 5	0	0	TRUE
101	Year 6	0	0	TRUE
102	Pre-Administration	0	0	TRUE
102	Month 4	0	0	TRUE
102	Month 8	0	0	TRUE
102	Month 12	0	0	TRUE
201	Pre-Administration	0	0	TRUE
201	Month 4	0	0	TRUE
201	Month 8	0	0	TRUE
201	Month 12	0	0	TRUE
202	Pre-Administration	0	0	TRUE
203	Pre-Administration	0	0	TRUE
203	Month 4	0	0	TRUE
203	Year 6	0	0	TRUE
301	Pre-Administration	0	0	TRUE
301	Month 4	0	0	TRUE
301	Month 8	0	0	TRUE
301	Month 12	0	0	TRUE
301	Year 2	0	0	TRUE
301	Year 3	0	0	TRUE
303	Pre-Administration	0	0	TRUE
303	Month 4	0	0	TRUE
303	Month 8	0	0	TRUE
303	Month 12	0	0	TRUE
303	Year 2	0	0	TRUE
303	Year 3	0	0	TRUE
204	Pre-Administration	0	0	TRUE
205	Pre-Administration	0	0	TRUE
205	Month 4	0	0	TRUE
205	Month 8	0	0	TRUE
205	Month 12	0	0	TRUE
206	Pre-Administration	0	0	TRUE
207	Pre-Administration	0	0	TRUE
207	Month 4	0	0	TRUE
207	Month 8	0	0	TRUE
207	Month 12	0	0	TRUE
207	Year 2	0	0	TRUE
305	Pre-Administration	0	0	TRUE
305	Month 4	0	0	TRUE
305	Month 8	0	0	TRUE
305	Month 12	0		TRUE
306	Pre-Administration	0	0	TRUE
307	Pre-Administration	0	0	TRUE

Table S3. Replication competent retrovirus detection. Real-time quantitative PCR for the Gibbon-Ape Leukemia Virus (GALV) envelope gene was performed using TaqMan probes, as described in Chen and Cornetta, Human Gene Therapy. 2001 Jan 1;12(1):61-70. doi: 10.1089/104303401450979. Patient CAR-T cell products were assessed for the presence of the GALV envelope, as were PBMC samples collected from patients at months 4, 8, and 12 and then yearly for up to 15 years. TestGene CP = Threshold Crossing Point for the GALV envelope gene.

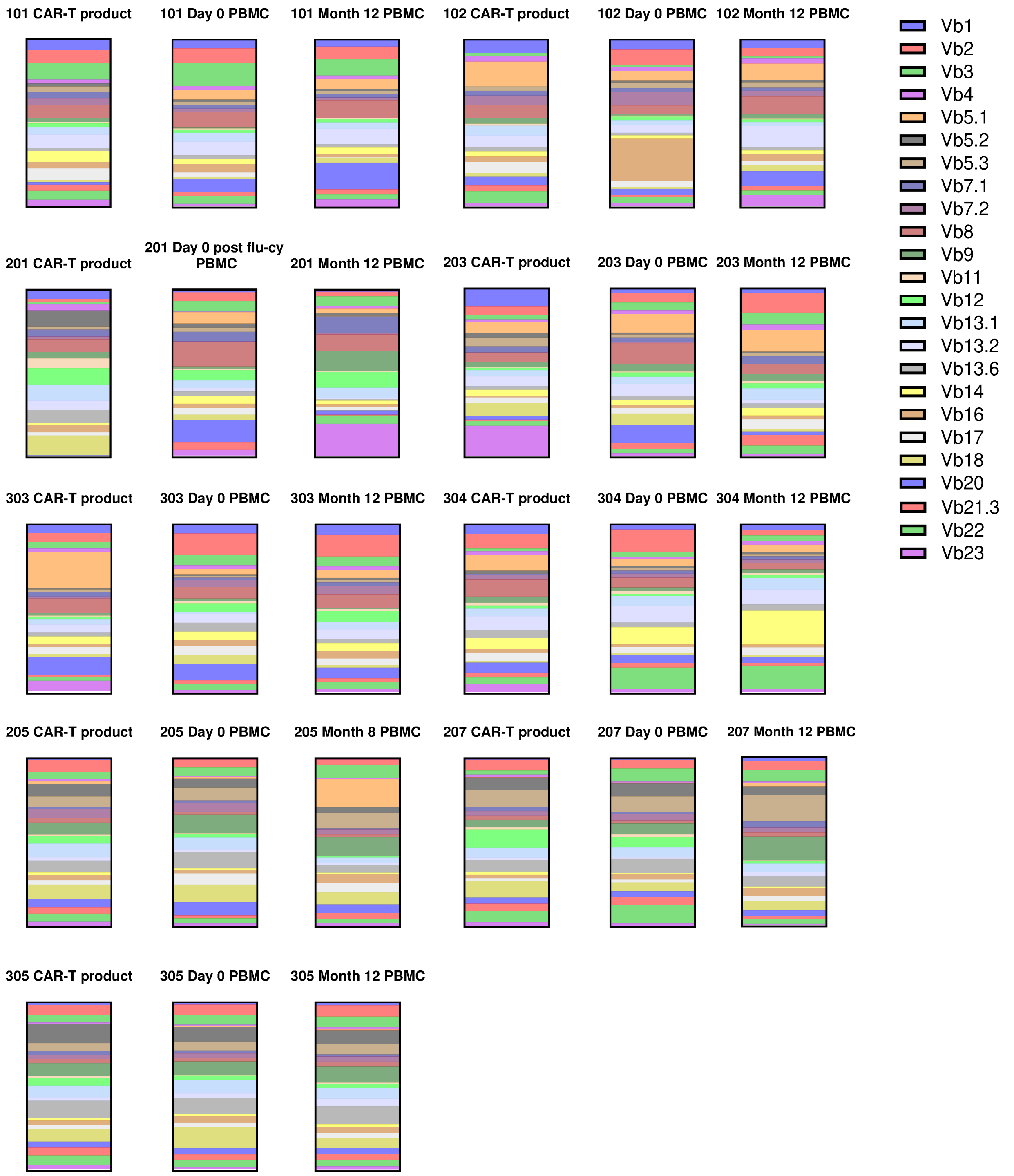

Figure S3 . TCR v-beta usage for CARPETS patients CAR-T cell product (column A), T cells from PBMC samples collected at day 0 pre-infusion (column B), and T cells from PBMC samples collected at month 8 or 12 post-infusion. PBMC were separated using density gradient and then stained using the Beta Mark TCR Vbeta Repertoire Kit and anti-CD3-PerCP-Cy5.5. Data are shown for 9 of 12 patients with data not shown for 3 patients who died of disease before month 4.

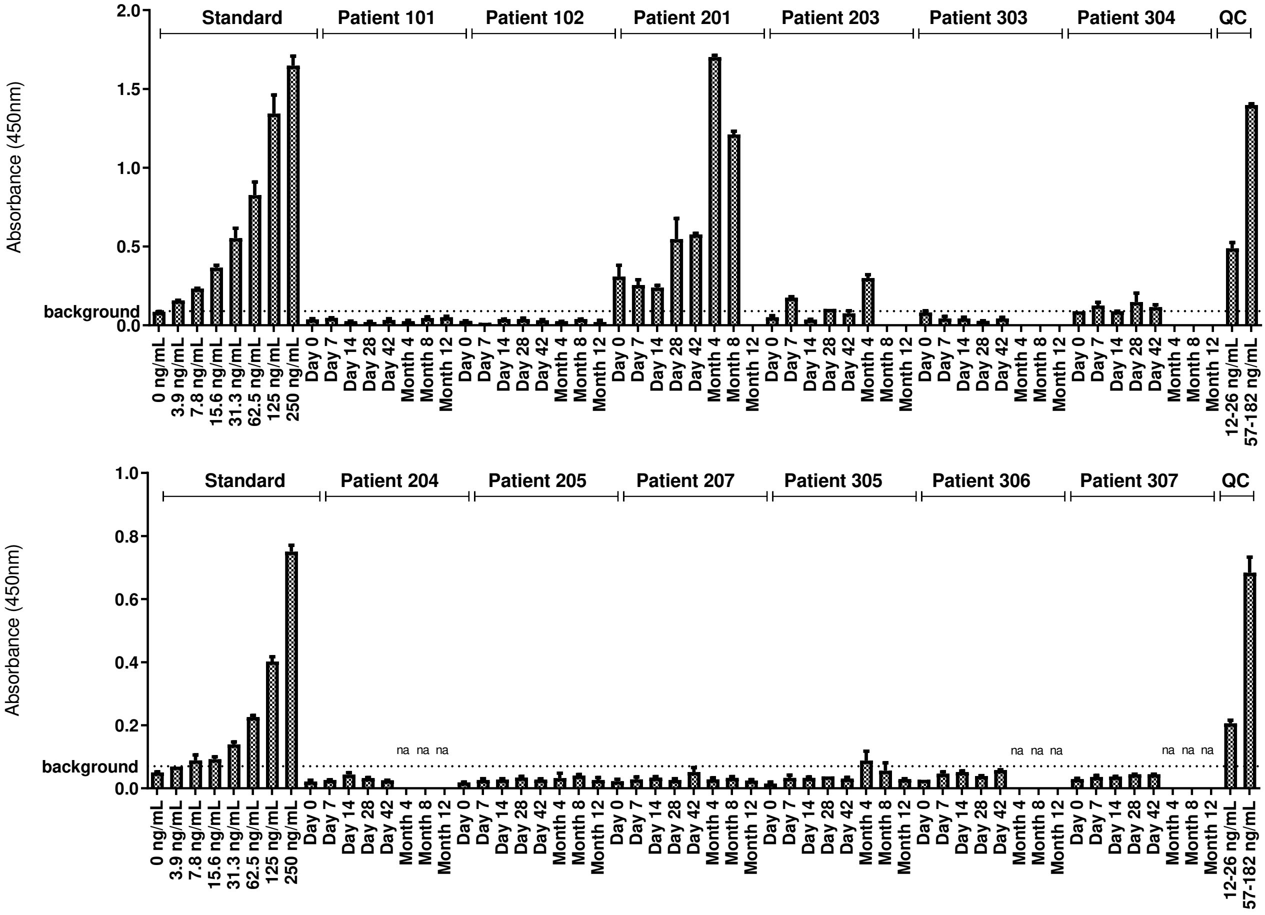

Figure S4. Human anti-mouse antibody (HAMA) detection. Serum was collected at day 0 (pre-infusion), and on days 7, 14, 28, 24 and months 4, 8, and 12 post-infusion. HAMA was determined using a LEGEND MAX Human anti-mouse Ig (HAMA) ELISA Kit. na = not available; Standard = HAMA standard diluted six-fold by 1 in 2 serial dilution; QC = pre-quantified HAMA samples for quality control. Patients were scored positive for HAMA when all three replicates above the mean background absorbance reading from negative control wells.

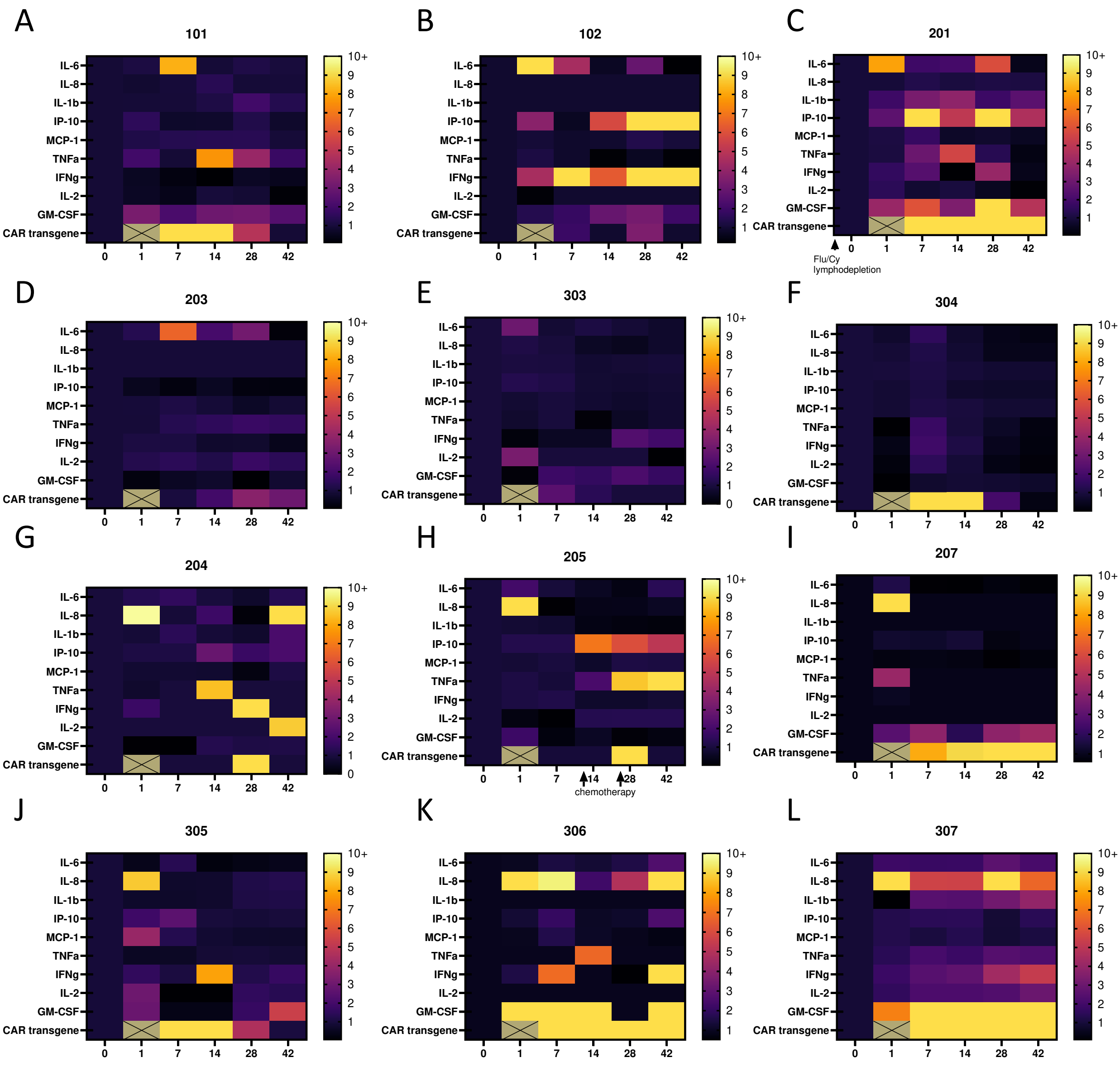

Figure S5. Summary of fold-change from day 0 baseline blood sample in peripheral biomarkers for each patient enrolled on CARPETS.

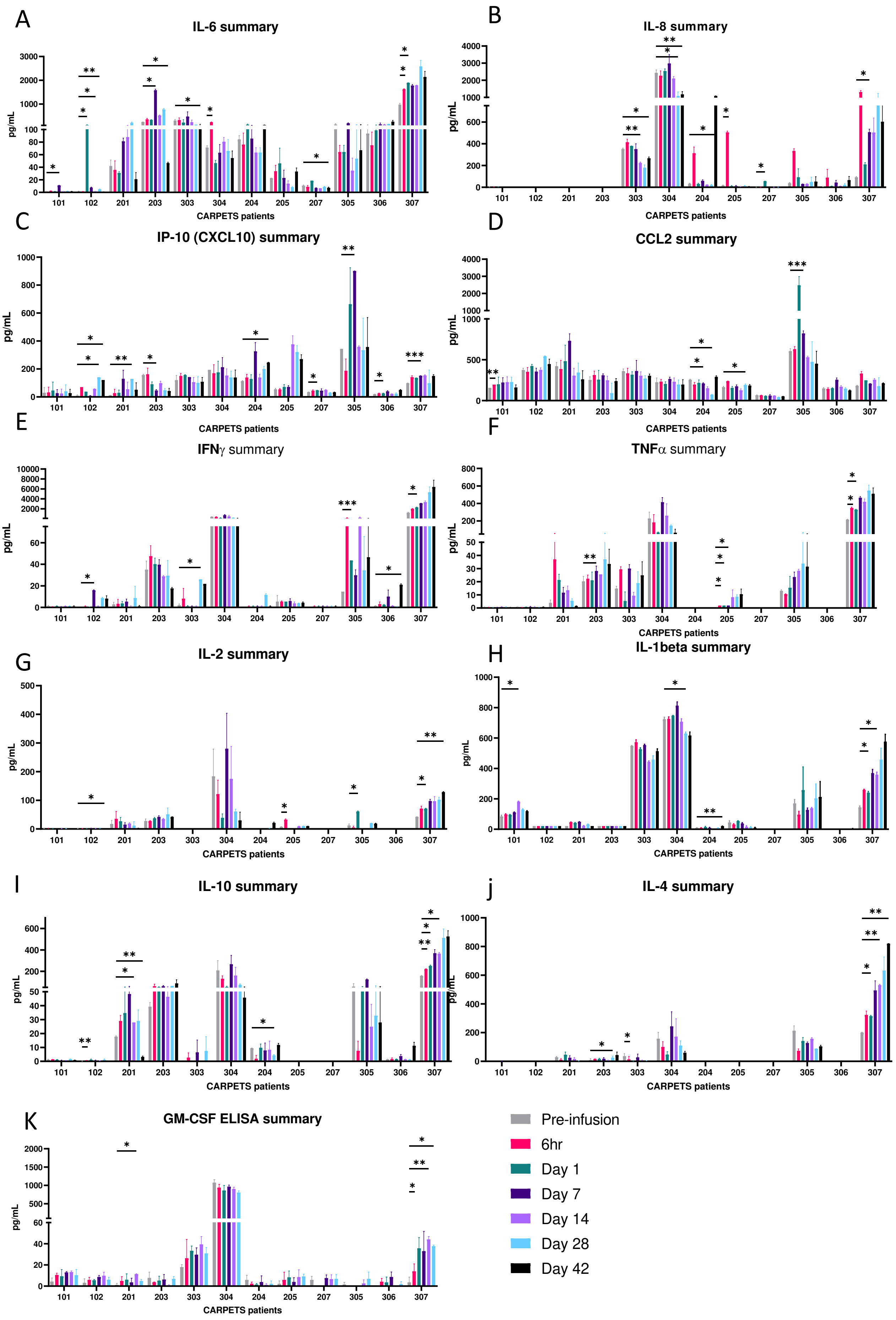
A
IL-6 summary
B
IL-8 summary
C
IP-10 (CXCL10) summary
D
CCL2 summary
E
IFNγ summary
F
TNFα summary
G
IL-2 summary
H
IL-1beta summary
I
IL-10 summary
j
IL-4 summary
K
GM-CSF ELISA summary
pg/mL
CARPETS patients
101
102
201
203
303
304
204
205
207
305
306
307
Pre-infusion
6hr
Day 1
Day 7
Day 14
Day 28
Day 42

Figure S6. Serum cytokine quantification in CARPETS study patients. Cytokines in patient sera collected at day 0 (pre-infusion), and at 6 hours, and at 1, 7, 14, 28, and 42 days after the CAR-T cell infusion. Values are shown for the 6 cytokines with consistently elevated levels in all trial patients: **A)** IL-6, **B)** IL-8 (CXCL8), **C)** IP-10 (CXCL10), **D)** MCP-1 (CCL2), **E)** IFN-gamma, **F)** TNF-alpha, **G)** IL-2, **H)** IL-1beta, **I)** IL-10, **J)** IL-4, and **K)** GM-CSF. Free-active TGF-beta, IL-12p70, IL-17A did not show consistent changes from baseline (pre-infusion) in most patients and are not shown. 13-plex cytokine detection by cytometric bead array (Biolegend). GM-CSF detection by ELISA (RandD systems). Significance was determined by one-way ANOVA with Dunnett's multiple comparisons test, *< 0.05, **<0.01, ***<0.001, ****<0.0001, GraphPad Prism 10.1.1.

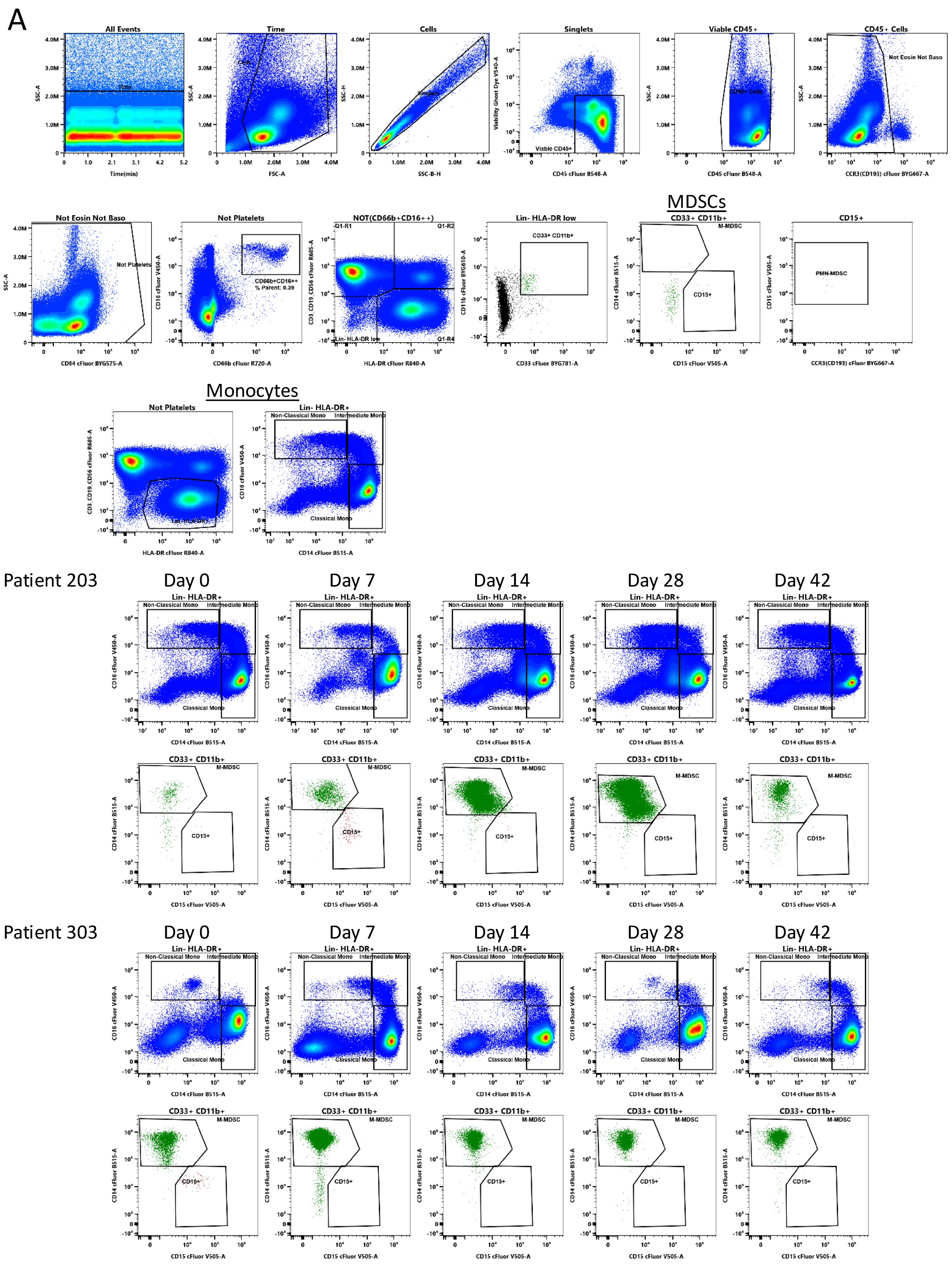
A
All Events
Time
Cells
Singlets
Viable CD45+
CD45+ Cells
MDSCs
Not Eosin Not Baso
Not Platelets
NOT(CD66b+CD16++)
Lin- HLA-DR low
CD33+ CD11b+
CD15+
Monocytes
Not Platelets
Lin- HLA-DR+
Patient 203
Day 0
Day 7
Day 14
Day 28
Day 42
Patient 303
Day 0
Day 7
Day 14
Day 28
Day 42

B

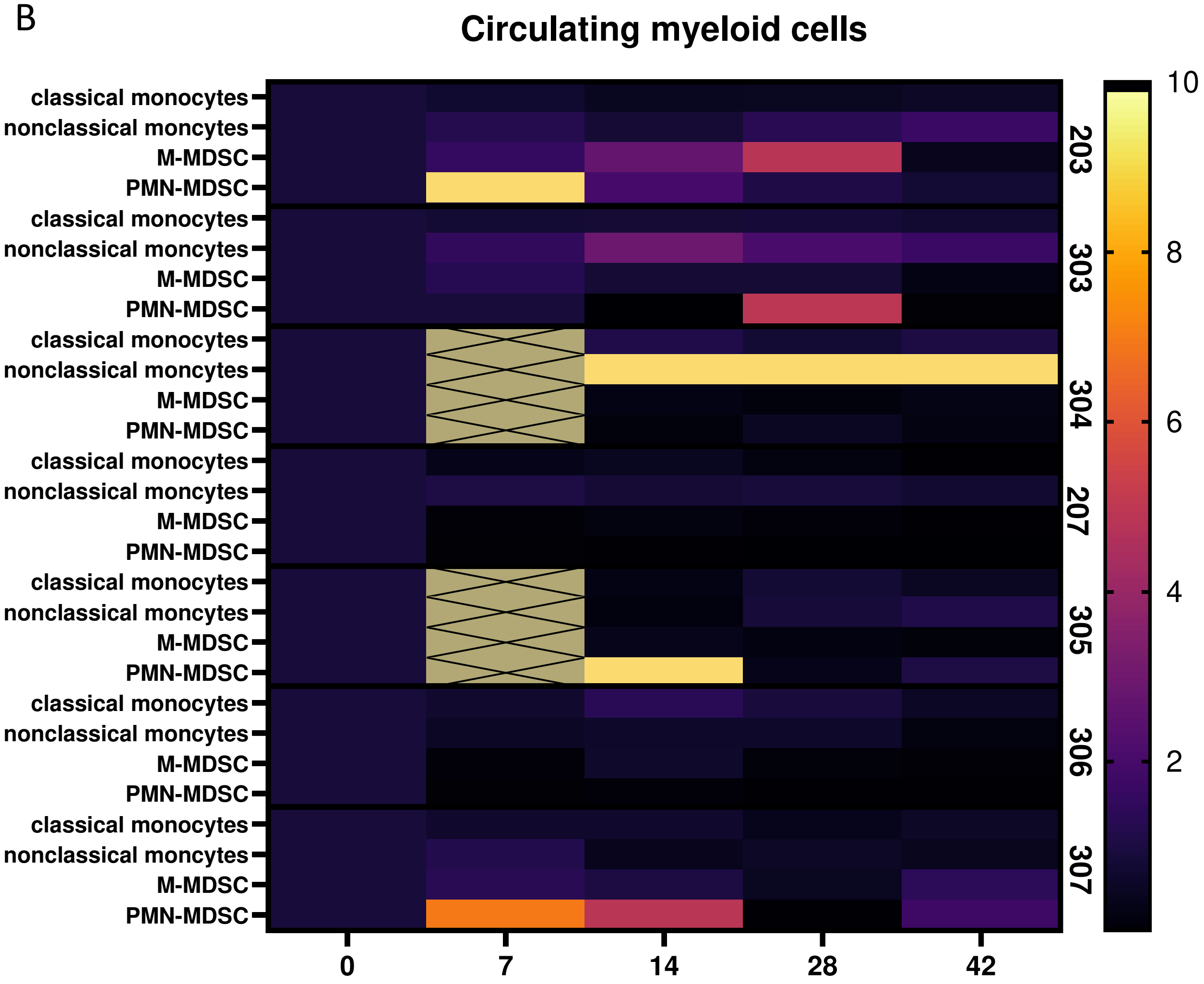

Figure S7. Analysis of circulating myeloid cells. **A)** Representative gating strategy showing the identification of Classical (Lin- HLADR+ CD14+) and Non-classical monocytes (Lin- HLADR+ CD16+); and Lin- HLA-DRlo/neg CD33+ CD11b Mononuclear-Myeloid Derived Suppressor Cells (M-MDSC); and representative dot plots of monocytes and polymorphonuclear (PMN)-MDSC (data from Patient 203 and 303 shown). **B)** Summary of fold-change from Day 0 baseline in Classical Monocytes; Non-Classical Monocytes; M-MDSC and PMN-MDSC for 7 CARPETS study patients. NB. Patients 304 and 305 had insufficient banked PBMC to assess myeloid populations for Day 7. Patients 101, 102, 201, 204 and 205 had insufficient banked PBMC to be included in the analysis.

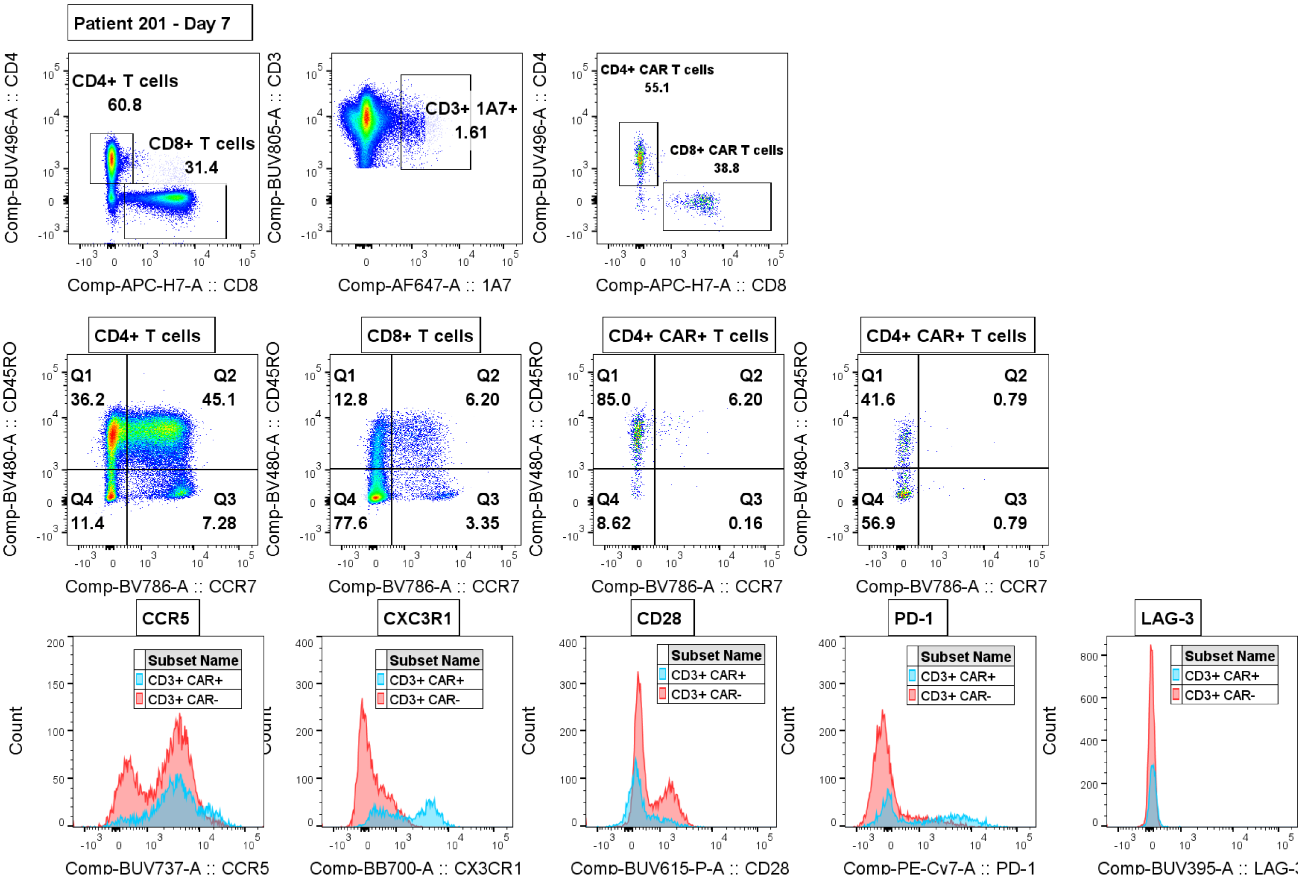

Figure S8. Gating strategy and representative plots for Patient 201 PBMC at day 7 post-infusion.

Patient ID	101	203	303	304	305	306
Time post-infusion	Year 3	Day 57	Year 2	Year 2	Day 21	Days -2, 1, 7, 14, 21
Sample type	Subcut. Biopsy	Subcut. Biopsy	Subcut. Biopsy x2	Subcut. Biopsy x2	Needle biopsy	Ascitic fluid
Storage	FFPE, OCT, single-cell	FFPE, OCT, single-cell	FFPE, single-cell	FFPE, signle-cell	FFPE, OCT, single-cell	single-cell
qPCR	Y	Y	Y	Y	Y	Y
Basic flow	Y	Y	Y	Y	Y	Y
IHC	Y	Y	Y	Y	Y	-
RNAScope	Y	Y	Y	Y	Y	-
IF	Y	Y	-	-	Y	-
High-parameter flow	-	Y	-	-	Y	Y

Table S4. Tumour samples collected post CAR-T cell infusion. Subcut. = subcutaneous; FFPE = formalin-fixed paraffin embedded tissue; OCT = optimal cutting temperature compound for fresh frozen tissue; single-cell = single cell suspension fresh frozen. Basic flow cytometry panel: CD3, CD4, CD8, 1A7. High Parameter flow cytometry panel as described for Figure 5 and 6.

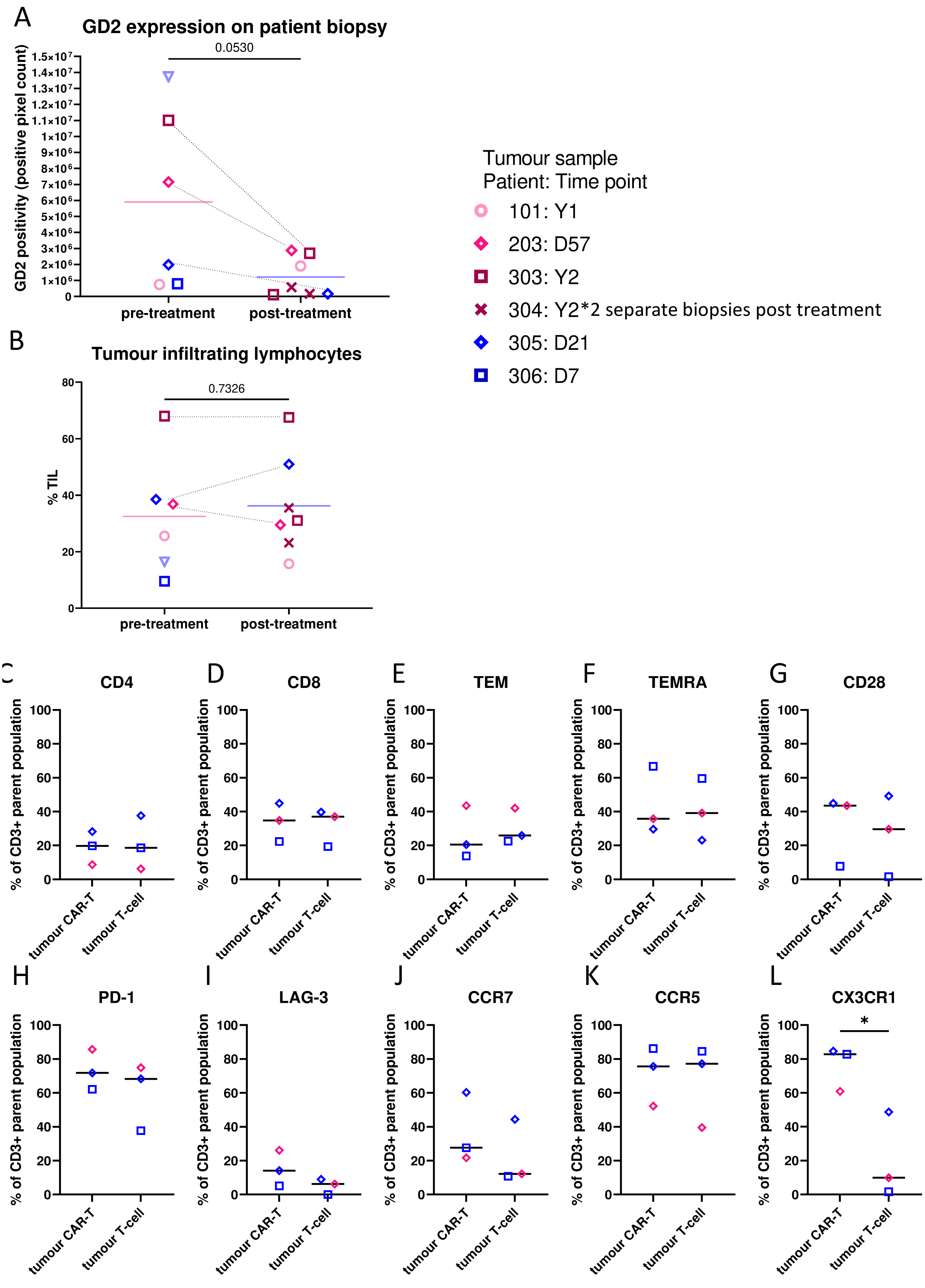
A
GD2 expression on patient biopsy
0.0530
GD2 positivity (positive pixel count)
pre-treatment
post-treatment
B
Tumour infiltrating lymphocytes
0.7326
% TIL
pre-treatment
post-treatment
Tumour sample
Patient: Time point
101: Y1
203: D57
303: Y2
304: Y2*2 separate biopsies post treatment
305: D21
306: D7
C
CD4
D
CD8
E
TEM
F
TEMRA
G
CD28
H
PD-1
I
LAG-3
J
CCR7
K
CCR5
L
CX3CR1
*
% of CD3+ parent population
tumour CAR-T
tumour T-cell

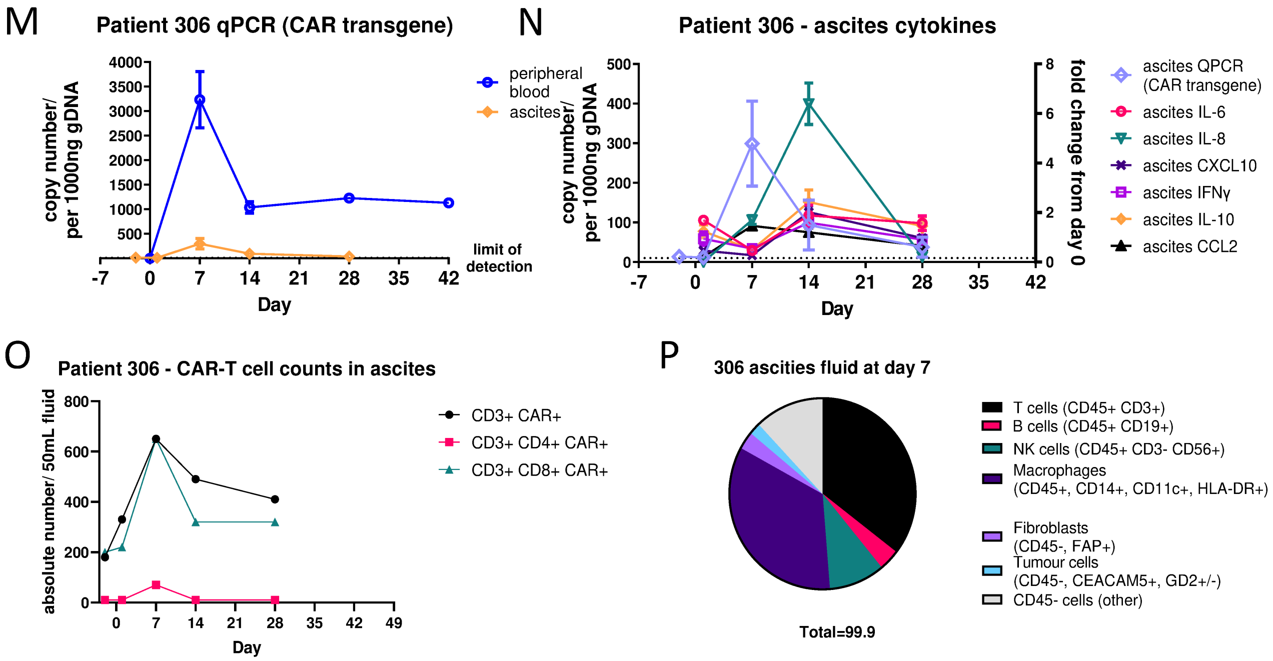

Figure S9. **A-B)** Assessment of GD2 staining intensity (positive pixel counts) and tumour infiltrating lymphocytes (percentage positive cell detection in total cell population). H&E and GD2 staining on archival tumour tissues was repeated on available FFPE samples and the whole tissue scanned by Nanozoomer slide scanner. Bioimage analysis was performed using QuPath automated cell counting, and analyses were performed by an independent pathologist in a blinded manner. Representative flow cytometry dot plots from staining on tumour biopsy samples. Percentage of viable CD3+ CAR+ T cells (infiltrating CAR-T cell population) or CD3+ CAR-T cells (tumour endogenous T cell population) that are **C)** CD4+, **D)** CD8+, **E)** Naïve (CCR7+ CD45RO- CD28**+**), **F)** TEMRA (CCR7-, CD45RO-, CD28-), **G)** CD28+, **H**) PD-1+, **I)** LAG-3+, **J)** CCR5+, **K)** CX3CR1+, or **L)** CCR7+**.** Significance was determined by one-way ANOVA with Dunnett's multiple comparisons test, *< 0.05, GraphPad Prism 10.1.1. Assessment in Patient 306 ascitic fluid collected at days -2, 0 (pre-infusion), 7, 14 and 28 post-infusion of CAR-T cells by **M)** quantitative PCR to detect the 14g2a scFv containing transgene using genomic DNA isolated from the cellular fraction of the ascites. **N)** Cytokine detection using filtered ascitic fluid. **O)** Absolute quantification of CAR-T cells by flow cytometry on the cellular fraction isolated from 50mL of ascitic fluid. **P)** Absolute quantification of T cells, NK cells, macrophages, and tumour cells by flow cytometry on the cellular fraction isolated from 50mL of ascitic fluid.

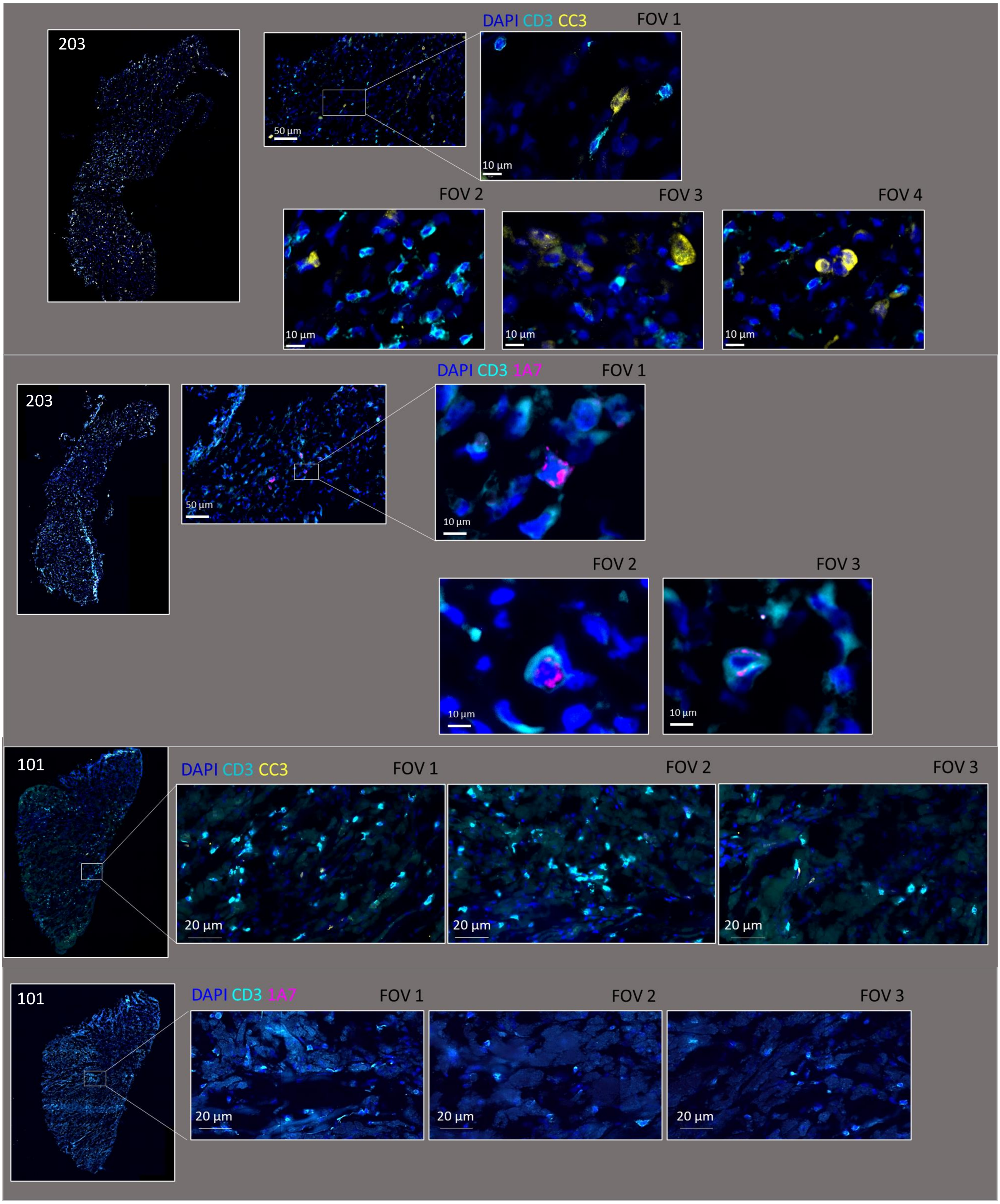

Figure S10. Immunofluorescence on post-treatment biopsy samples. Fresh-frozen whole tissue sections were sectioned (5 micron thickness) and stained with DAPI, anti-human CD3 and anti-CAR 1A7 anti-idiotypic monoclonal antibody, and anti-cleaved caspase 3 (CC3), in 3-parameter panels on serial sections. Whole-slide imaging was performed on a Zeiss Axio Scan.Z1 slide-scanner using 20x objective and ZEN 3.1 Blue system software. Staining for Patient 203 (Day 57 biopsy) Patient 101 (Year 1 biopsy), are shown. FOV = field of view.
