# Supplementary material for: Safety and biological outcomes following a phase 1 trial of GD2-specific CAR-T cells in patients with GD2-positive metastatic melanoma and other solid cancers
Source: J Immunother Cancer. 2024 May 15;12(5):e008659. doi: 10.1136/jitc-2023-008659 (PMC11097842; doi:10.1136/jitc-2023-008659)
Supplement: Supplementary data [file jitc-2023-008659supp002.pdf]

BMJ Publishing Group Limited (BMJ) disclaims all liability and responsibility arising from any reliance placed on this supplemental material which has been supplied by the author(s)

Supplementary Methods

Endpoints

The primary objectives of this study were to determine: 1. The feasibility of preparing T cell products for administration to patients with GD2-positive malignancy; 2. The safety profile and dose limiting toxicities of autologous peripheral blood T cells directed to GD2 through their chimeric antigen receptor (GD2-iCAR-PBT as the T cell product) in patients with GD2-positive malignancy. Secondary objectives were: To assess in vivo persistence of infused GD2-iCAR-PBT; To assess tumour infiltration by infused GD2-iCAR-PBT; To document persistence of anti-tumour effects after the infusion of GD2-iCAR-PBT as measured by partial or complete tumour response or stable disease.

Evaluation criteria - Toxicity. Toxicity was evaluated by the NCI Common Terminology Criteria for Adverse Events version 4.03. A DLT is an event considered to be primarily related to the PBT infusion if it occurs at any time up to 6 weeks from the PBT infusion and is defined as ≥ CTCAE version 4.0 grade 3 toxicity (not recovered within 5 days); non-haematological toxicities of any duration, which are severe enough and deemed DLT after discussion with the medical monitor; grade 3 hypersensitivity reaction, which did not respond to H1 and H2 blockade, recurred after prophylactic H1 and H2 blockade or any Grade 4 reaction; grade 3 fever (> 40.0℃ ≤ 24 hours), which did not respond to interruption of dabrafenib or of dabrafenib and trametinib or to dose reduction or oral prednisolone (up to 25 mg mane) or any Grade 4 fever (> 40.0℃ > 24 hours) associated with hypotension (SBP < 100 mm Hg); grade 3 chills or flu-like symptoms lasting more than 5 days, which did not respond to interruption of dabrafenib or of dabrafenib and trametinib or to dose reduction or oral prednisolone (up to 25 mg mane) or any Grade 4 reaction associated with hypotension (SBP < 100 mm Hg); and Grade 3 or 4 Cytokine Release Syndrome (CRS).

Evaluation criteria – tumour response. Tumour response was determined for all patients with measurable lesions using the international criteria proposed by the revised Response Evaluation Criteria in Solid Tumours (RECIST) guideline (version 1.1). Changes in the largest diameter (unidimensional measurement) of the tumour lesions and the shortest diameter in the case of malignant lymph nodes are used in the RECIST 1.1 criteria. The first assessment was made at 6 weeks after the GD2-iCAR-PBT infusion.

Flow cytometry panels

Panel A – T cells: CD3-AF488, CD4-bv510, CD8-PeCy7, CD45RA-AF647, CCR7-bv421, (all BD biosciences) with 1A7 (in house; anti-idiotypic CAR antibody)-PE and Panel B – B cells, monocytes, and NK cells: CD3-AF488, CD19-bv519, CD14-AF647, CD16-PeCy7 and CD56-bv421 (all BD biosciences). Samples were analysed on the BD Fortessa LSR Cytometer. For advanced, high-parameter phenotyping of circulating CAR-T cells, the following panel was used: CD3 buv805, CD4 buv496, CD8 APCH7, CD45RO bv480, PD-1 PeCy7, LAG-3 buv395, CD28-buv615, CCR7-bv786, CCR5-buv737, CXCR6-bv421, CX3CR1-BB700, L/D FVS575V; (all BD biosciences) with 1A7 anti-CAR antibody-AF647 (in house).

Immunofluorescence and RNAScope on tumour tissue sections

IF staining was performed on fresh-frozen tissue sections (5-6µm thickness). Tissue sections were stained using 5µg/mL purified mouse anti-human CD3 (UCHT1; BioLegend); 0.2 ug/mL anti-idiotypic CAR antibody (1A7; in-house purified from hybridoma); or anti-human Cleaved Caspase 3 (1:200, Asp175; Cell Signaling Technology) with goat anti-rabbit IgG AF55+, goat anti-mouse IgG AF555+ and goat anti-mouse IgG AF647+ secondary (1:1000; Thermo Fisher Scientific). Fluorescence overlays

OFFICIAL

were created by merging channels and applying false colour using FIJI (ImageJ, National Institutes of Health) and Qupath[24].

The RNAScope HD 2.5 Duplex system (ACDbio) was used to detect infiltrating CAR-T cells in FFPE sections using probes for CD3 and retroviral mRNA (Hs-CD3E-No-XMm-C2 and V-PMX-retroLTR). Whole-slide imaging was performed on a Zeiss Axio Scan.Z1 slide-scanner using 40x objective and ZEN 3.1 Blue system software.

Tissue staining analysis by QuPath

The area around each sample was digitally selected before cell detection methods were utilised. For the H&E samples, the object classifier tool was used on small sections of multiple tissue samples to train QuPath to improve accuracy in detecting tumour infiltrating lymphocytes. Following this, a threshold was used to determine the total number of tumour infiltrating lymphocytes for entire images. The following threshold parameters were used: detection image: H&E; requested pixel size: 0.5; background radius: 8; Opening reconstruction was used; mean filter radius: 0; sigma 1.5; minimum area 10; maximum area 400; threshold: 0.1; maximum background intensity: 2; split by shape; cell expansion: 1mml; include cell nucleus; smooth boundaries; score component nucleus: eosin; OD mean; threshold: 0.27. Once a positive cell count, negative cell count and total cell count were obtained for each sample, these values were imported into excel and graphed. For the G2D expression, the positive pixel detection feature was used to determine proportion of G2D staining per sample. The threshold parameters used were as follows: resolution high: 0.91; channel: DAB; pre filter: gaussian; soothing sigma: 1; threshold: 0.25; above threshold: positive; below threshold: unclassified; region: everywhere. A value was obtained for positive detections (GD2 staining), negative detections (non-GD2 staining) and total detections. The proportion of GD2 staining was determined from these values and this was graphed.

Statistics

Descriptive statistics were used for this Phase 1 study. Quantitative variables are described in terms of mean and standard error from the mean for each cohort (dose level) and for the overall population. Data were analyzed using GraphPad Prism Version 10.1.1. Two independent groups were compared using unpaired t-test. Multi-group single-variable data were analyzed by one-way ANOVA and Tukey's multiple comparison post-tests. Statistical significance is represented on graphs as * ≤ 0.05, ** ≤ 0.01 and *** ≤ 0.001.

OFFICIAL
