# Supplementary material for: Safety and biological outcomes following a phase 1 trial of GD2-specific CAR-T cells in patients with GD2-positive metastatic melanoma and other solid cancers
Source: J Immunother Cancer. 2024 May 15;12(5):e008659. doi: 10.1136/jitc-2023-008659 (PMC11097842; doi:10.1136/jitc-2023-008659)
Supplement: Supplementary data [file jitc-2023-008659supp003.pdf]

Confidential

CLINICAL PROTOCOL - CARPETS

A PHASE I STUDY OF THE SAFETY AND IMMUNE EFFECTS OF AN ESCALATING DOSE OF AUTOLOGOUS GD2 CHIMERIC ANTIGEN RECEPTOR-EXPRESSING PERIPHERAL BLOOD T CELLS IN PATIENTS WITH GD2-POSITIVE METASTATIC MELANOMA AND REFRACTORY SOLID TUMOURS

Sponsor: Royal Adelaide Hospital Cancer Centre
6E351,
Port Road, Adelaide SA 5000

Principal Investigator: Prof. Michael Brown

Co-investigators: Dr Shireen Sidhu, MBBS FRCP FACD
Royal Adelaide Hospital
Port Road, Adelaide SA 5000

Medical Monitor: Professor Paul Rolan MD FRACP FFPM FFPMANZCA
Affiliate Professor
School of Medical Sciences, The University of Adelaide
5 Tregenza Close
Beaumont SA 5066
E: paul.rolan@adelaide.edu.au
M: +61 405 670 420

Safety Monitor: Ms Anne Milton RN
Cancer Clinical Trials Unit
6E351, Port Road, Royal Adelaide Hospital SA 5000

Statistician: Dr Kerri Beckman
School of Public Health, Division of Health Sciences
University of South Australia Cancer Research Institute
North Terrace, Adelaide SA 5000

RAH No. 100524e	**Date**
VERSION 1.9c	01 October 2021

Gargett T, *et al. J Immunother Cancer* 2024; 12:e008659. doi: 10.1136/jitc-2023-008659

CONFIDENTIAL

INVESTIGATORS STATEMENT

I,……………………………………………………….the undersigned, understand that the Study will not be started without the prior written approval of the Human Research Ethics Committee. No changes will be made to the study protocol without the prior written approval of the Sponsor and the Research Ethics Committee.

I have read, understood, and agree to abide by all the conditions and instructions contained in this protocol. I agree to comply with the ICH Harmonized Tripartite Guideline for Good Clinical Practice for conducting clinical trials and local regulations and will conduct the above study under these standards.

__________________ __________________

Principal Investigator Date (day/month/year)

Gargett T, *et al. J Immunother Cancer* 2024; 12:e008659. doi: 10.1136/jitc-2023-008659

CONFIDENTIAL

1. PROTOCOL SUMMARY

**Protocol Name:**	CARPETS
**Protocol Title:**	A Phase 1 Study of the Safety and Immune effects of an Escalating Dose of Autologous GD2 Chimeric Antigen Receptor-Expressing Peripheral Blood T cells in Patients with GD2-Positive Metastatic Melanoma and Refractory Solid Tumors
**Study Phase:**	Phase 1
**Study Design**	Patients who have a Hb ≥ 105g/L and unresectable metastatic melanoma, which is either BRAF gene mutation negative or positive (BRAF V600E/K/R/D mutations) or other GD2-positive malignancies, will be asked to consent to the study. Patients with BRAF-mutant melanoma must be eligible to receive dabrafenib and trametinib. The consent will allow initial testing of the patients' (i) blood samples for evidence of active infection with human immunodeficiency virus 1 or 2 (HIV1/2), hepatitis B virus (HBV), and hepatitis C virus (HCV), and (ii) archived or fresh tumour tissue samples for GD2 expression. These tests have a turn-around time of up to 2 days.<br><br>If patients do not have evidence of active infection with HIV1/2, HBV or HCV and have tumour expression of GD2 then patients will be asked to provide a 360mL blood sample for (i) generation of the T-cell product (180mL), (ii) isolation and storage of peripheral blood mononuclear cells (PBMC) for later immunological testing (80mL), (iii) baseline studies and routine haematology and biochemistry (MBA20) tests (100mL).<br><br>Patients who are eligible to receive dabrafenib and trametinib will receive an initial dose of dabrafenib 150 mg bd and trametinib 2 mg od while their T-cell product is being prepared. As part of disease evaluation on the BRAF/MEK inhibitor combination therapy, patients will have CT scans every 9 to 12 weeks.<br><br>It is anticipated that it will take 3 weeks to prepare a qualified autologous T-cell product. Patients will receive a single intravenous injection of the

Gargett T, *et al. J Immunother Cancer* 2024; 12:e008659. doi: 10.1136/jitc-2023-008659

	previously prepared T-cell product, which is a dose of GD2-iCAR-PBT. The subsequent treatment evaluation period is 6 weeks.<br><br>If a patient not receiving dabrafenib or trametinib has a confirmed complete or partial response without evidence of dose-limiting toxicity (DLT) at their subsequent CT scan evaluations, then a written request will be made to the Research Ethics Committee to consider two additional infusions of GD2-iCAR-PBT, if available and if eligibility criteria for administration continue to be met. The gene-modified T cells for any additional infusions will have been made and qualified before the initial dose. An additional infusion would be at the previous cell dose and would be administered no less than 6 weeks after the first dose when the initial safety profile is completed. A similar 6-week safety evaluation period would follow the second or third infusions using the same Assessment Schedule.<br><br>Since virtually all of the patients who receive dabrafenib and trametinib will achieve stabilization or shrinkage of their disease, these patients will not receive further infusions of GD2-iCAR-PBT.<br><br>The study will be of open-label, single ascending dose escalation design, and will employ the Bayesian dose-finding modified continual reassessment method (mCRM) to determine the safety of one intravenous injection of autologous PBT directed to GD2 through the chimeric antigen receptor (CAR) in patients with GD2-positive melanoma, which is either BRAF mutation negative or BRAF mutation positive (containing dabrafenib-eligible *BRAF* gene mutations at the V600 codon i.e. V600E/K/R/D) or with other GD2-positive malignancies. The mCRM minimises the number of patients who may be exposed to an ineffective dose. Given the lack of adverse events in the first-in-human study using the related first-generation GD2-CARζ vector and in the completed GRAIN study in neuroblastoma patients using the same GD2-iCAR vector (both studies hosted at Baylor College of Medicine, Houston, USA), and our own favourable safety data concerning the first six melanoma patients enrolled via the CARPETS protocol, the proposed dosing schedule of the GD2-iCAR-T PBT product

	made under a new set of ex vivo cell manufacturing conditions comprises the following dose levels:<br>Dose Level 2: 2 x10^7 cells/m^2<br>Dose Level 3: 1 x 10^8 cells/m^2<br>Each patient will be followed for 6 weeks after the PBT infusion for evaluation of dose limiting toxicity (DLT). The toxicity will be evaluated by the NCI Common Terminology Criteria for Adverse Events version 4.0. A DLT is an event considered to be primarily related to the PBT infusion if it occurs at any time up to 6 weeks from the PBT infusion and is defined as ≥ CTCAE version 4.0 grade 3 toxicity (not recovered within 5 days); non-haematological toxicities of any duration, which are severe enough and deemed DLT after discussion with the medical monitor; grade 3 hypersensitivity reaction, which did not respond to H1 and H2 blockade, recurred after prophylactic H1 and H2 blockade or any Grade 4 reaction; Grade 3 fever (>40.0˚C ≤ 24 hours), which did not respond to interruption of dabrafenib and trametinib, or to dose reduction or oral prednisolone (up to 25 mg mane) or any Grade 4 fever (> 40.0˚C > 24 hours) associated with hypotension (SBP < 100 mm Hg); grade 3 chills or flu-like symptoms lasting more than 5 days, which did not respond to interruption of dabrafenib and trametinib, or to dose reduction or oral prednisolone (up to 25 mg mane) or any Grade 4 reaction associated with hypotension (SBP < 100 mm Hg); and Grade 3 or 4 Cytokine Release Syndrome (CRS) (see Appendix V).<br>A DLT may trigger the administration of rimiducid (AP1903), which itself is defined as a DLT. For example, Grade 3 CRS not responding to standard clinical management with supportive measures, dexamethasone, and tocilizumab, or Grade 4 CRS will trigger the administration of rimiducid. The indication for rimiducid administration will be discussed with the medical monitor. Rimiducid is a bioinert small-molecule drug that activates a cellular suicide program in GD2-iCAR-PBT, which results in their immediate destruction and thus may terminate any GD2-iCAR-PBT-related adverse events.

	For this trial, the maximum tolerated dose (MTD) is defined to be the dose that causes a DLT in 20% of patients. All patients within a dose cohort should have completed the 6-week window after the PBT infusion for assessment of DLT prior to enrolment of patients into the next recommended dose level. To facilitate the dose escalation process, there will be a review of the safety data by the Medical Monitor and Principal Investigator who will provide this information to the data safety monitoring board (DSMB) as described in detail in section 12.3.6. The DSMB will provide a written recommendation on a decision to dose escalate based on protocol specified criteria and advise the study site accordingly. Copies of the documentation will be retained in the study site file.<br><br>The mCRM will employ a cohort of size 2 based on the exponential dose-toxicity model. The fixed prior probabilities of DLT for the three dose levels indicated above were assumed to be 5%, 8% and 25%, respectively. To ensure patient safety, the mCRM starts from the lowest dose level and limits the dose escalation to one dose level at a time. A DLT event in the lower dose cohort will be used to update the dose-toxicity curve. The next patient cohort is assigned to the dose level with an associated probability of DLT closest to the target probability of 20%. This process continues until at least 10 patients have been accrued into the trial or 6 patients have been treated at the current MTD. Depending on patient availability and dose escalation, a maximum of 15 patients will be recruited into this Phase I trial. The final MTD will be the dose with probability closest to the target toxicity rate at these termination points. Since GD2-iCAR-PBT generation for this trial takes 3 weeks, up to 15 T-cell products may need to be generated to allow for potential ‘drop outs’ and change in patients’ eligibility status (80% completion rate).<br><br>During the study, patient toxicity outcomes will be monitored in real time in order to estimate the dose-toxicity curve and determine the dose level for the next patient cohort using one of the pre-specified dose levels**.** Patients in a cohort can be enrolled concurrently but both patients within a dose cohort should have completed the 6-week window after the PBT infusion for assessment of DLT prior to enrolment of patients into the next recommended

	dose level. To increase safety, patients enrolled in the same dose cohort will not be treated within the same week. The recommended phase II single agent dose will be based on the findings of the toxicity, immune effects, and activity profile of the patients in this phase I study.
**No. of Patients:**	Up to 12 evaluable patients
**Primary Objectives:**	The primary objectives of this study are to determine:<br>1. The feasibility of preparing T cell products for administration to patients with GD2-positive malignancy<br>2. The safety profile and dose limiting toxicities of autologous peripheral blood T cells directed to GD2 through their chimeric antigen receptor (GD2-iCAR-PBT as the T cell product) in patients with GD2-positive malignancy
**Secondary Objective:**	The secondary objectives of this study are:<br>1. To assess *in vivo* persistence of infused GD2-iCAR-PBT<br>2. To assess tumour infiltration by infused GD2-iCAR-PBT<br>3. To assess bystander anti-melanoma immune effects of BRAF inhibitor therapy and infused GD2-iCAR-PBT<br>4. To document persistence of anti-tumour effects after the infusion of GD2-iCAR-PBT as measured by partial or complete tumour response or stable disease.
**Assessments:**	**Screening**: Screening assessments will be completed within 28 days prior to administration of GD2-iCAR-PBT.<br>• Blood sampling for PBMC isolation (180mL) to prepare the T-cell product, which will be stored frozen for later use<br>• Blood sampling for dendritic cell (DC) production and banking (5 x 9mL Li heparin tubes). DC to be stored frozen in ready to use aliquots for subsequent immunological assays<br>• Blood sampling for PBMC isolation (7 x 9mL Li heparin tubes) to be stored frozen as a baseline for immunological assays including

	assay of anti-iCasp9 immune responses and fluorocytometric analysis of TCR Vβ oligoclonality<br>• Stored plasma for anti-tumour antibody studies and GD2 quantification studies (from above tubes)<br>• Serum storage for human anti-mouse antibody studies (baseline value) (5mL)<br>• EDTA blood for GD2-iCAR-PBT persistence studies (5mL)<br>• MBA20, C-reactive protein (CRP), serum pregnancy (10mL)<br>• HIV, Hepatitis B & C testing<br>• Haematology (5mL)<br>• Serum cytokines (5mL red top [clot activation] tube)<br>• PBMC isolation and storage for RCR testing (1 x 9mL Li heparin tubes)<br>• Urinalysis<br>• An optional pre-treatment tumour biopsy may be collected<br>• Baseline radiographic scanning for disease evaluation performed within 28 (± 4) days prior to administration of GD2-iCAR-PBT.<br>• Full skin assessment by a designated dermatologist will be done in the 28-day period before commencing dabrafenib and trametinib, at 8 and 16 weeks after commencing dabrafenib and trametinib, then approximately every 12 weeks thereafter while the patient continues to take dabrafenib and trametinib<br><br>It is anticipated that preparation of the autologous T-cell product will take 3 weeks from the time of collection in the screening period.<br><br>**Day 0 (day of GD2-iCAR-PBT infusion)**:<br>• MBA20, and Haematology (15mL) and baseline serum cytokine and CRP measurement (5mL)<br>• GD2-iCAR-PBT persistence studies using EDTA blood (5mL) for DNA-PCR and 2 x 9mL Li heparin tubes for flow cytometry (20mL)<br>• Urinalysis

	• <u>*GD2-iCAR-PBT dose*</u><br>• Serum cytokine and CRP measurements (5mL), and GD2-iCAR-PBT persistence studies (25mL), 6 hours after administration of GD2-iCAR-PBT<br><br>**Day 1:**<br>• MBA20, serum cytokine and CRP measurement 24 hours after administration of T-cell product (15mL)<br><br>**Days 4 to 14:**<br>An optional post-treatment tumour biopsy may be collected at any time between Days 4 to 14 post-infusion<br><br>**Day 7 (± 1):**<br>• MBA20 and Haematology (15mL)<br>• Serum cytokine and CRP measurement (5mL)<br>• GD2-iCAR-PBT persistence studies, and RNA analysis (25mL)<br>• Urinalysis<br><br>**Days 14 (± 1) and 28 (± 1):**<br>• MBA20 and Haematology (15mL)<br>• Serum cytokine and CRP measurement (5mL)<br>• GD2-iCAR-PBT persistence studies, and RNA analysis (25mL)<br>• PBMC isolation and storage for i*n vitro* analysis of immune response (4 x 9mL Li heparin tubes)<br>• Stored plasma for anti-tumour antibody studies and GD2 quantification studies (from above tubes)<br>• Urinalysis<br><br>**Day 42 (± 1):**<br>• MBA20 and Haematology (15mL)

Gargett T, *et al. J Immunother Cancer* 2024; 12:e008659. doi: 10.1136/jitc-2023-008659

	• Serum cytokine and CRP measurement (5mL)<br>• Serum storage for human anti-mouse antibody studies (baseline value) (5mL)<br>• GD2-iCAR-PBT persistence studies, and RNA analysis (25mL)<br>• PBMC isolation and storage for i*n vitro* analysis of immune response (4 x 9mL Li heparin tubes) and for *in vitro* analysis of cellular anti-iCasp9 immune responses (3 x 9mL Li heparin tubes)<br>• Stored plasma for anti-tumour antibody studies and GD2 quantification studies (from above tubes)<br>• Urinalysis<br><br>**Follow-up testing begins at 4, 8, 12 months, and yearly for a total of 15 years (as per Assessment Schedule Section 10):**<br>• PBMC isolation and storage for RCR testing (1 x 9mL Li heparin tubes)<br>• Serum storage for human anti-mouse antibody studies (5mL)<br>• PBMC isolation and storage for i*n vitro* analysis of cellular anti-iCasp9 immune responses and fluorocytometric analysis of TCR Vβ oligoclonality (5 x 9mL Li heparin tubes)
**DLT Definition:**	An event will be considered a dose-limiting toxicity (DLT) if it occurs at any time up to 6 weeks from the PBT infusion, is considered to be primarily related to GD2-iCAR-PBT, and meets one of the criteria below despite maximum provision of supportive care.<br>a. CTCAE version 4.0 Grade 3 or higher toxicity that is not recovered within 5 days;<br>Some non-haematological toxicities of any duration are severe enough and will be deemed DLT after discussion with the medical monitor.

Gargett T, *et al. J Immunother Cancer* 2024; 12:e008659. doi: 10.1136/jitc-2023-008659

	b. Grade 3 hypersensitivity reaction which did not respond to H1 and H2 blockade, recurred after prophylactic H1 and H2 blockade or any Grade 4 reaction.<br>c. Grade 3 fever (> 40.0˚C ≤ 24 hours), which did not respond to interruption of dabrafenib and trametinib, or to dose reduction or oral prednisolone (up to 25 mg mane) or any Grade 4 fever (> 40.0˚C > 24 hours) associated with hypotension (SBP < 100 mm Hg).<br>d. Grade 3 chills or flu-like symptoms lasting more than 5 days, which did not respond to interruption of dabrafenib and trametinib, or to dose reduction or oral prednisolone (up to 25 mg mane) or any Grade 4 reaction associated with hypotension (SBP < 100 mm Hg).<br>e. Grade 3 or 4 Cytokine Release Syndrome (CRS) (see Appendix V).<br>f. A DLT may trigger the administration of rimiducid, which itself is defined as a DLT. For example, Grade 3 CRS not responding to standard clinical management with supportive measures, dexamethasone, and tocilizumab, or Grade 4 CRS will trigger the administration of rimiducid. The indication for rimiducid administration will be discussed with the medical monitor.
**Duration of Therapy:**	Patients will receive dabrafenib and trametinib until disease progression or drug intolerance.<br><br>Patients will be treated with a single dose of GD2-iCAR-PBT.
**Study Population:**	**Inclusion Criteria**<br>1. At least 18 years old;<br>2. Histological diagnosis of small cell lung cancer, triple negative breast cancer, osteosarcoma, Ewing sarcoma, and such soft tissue sarcomas as rhabdomyosarcoma, liposarcoma, fibrosarcoma, synovial sarcoma, pleomorphic undifferentiated sarcoma, and desmoplastic small round cell tumours (DSRCT), or metastatic melanoma (surgically incurable and unresectable stage III or stage

Gargett T, et al. J Immunother Cancer 2024; 12:e008659. doi: 10.1136/jitc-2023-008659

	IV; AJCC Cancer Staging Manual, 7th edition, 2010) and ≥ 10% GD2 positive cells (by an independent pathologist); patients with other malignancies with ≥ 10% GD2 positive cells are also eligible;<br>3. Unresectable stage III melanoma must have confirmation from a surgical oncologist. Melanoma will have V600 *BRAF* gene mutation status determined; V600E, K, R, or D mutations may be eligible for treatment with dabrafenib and trametinib;<br>4. Must have failed standard therapy;<br>5. For metastatic melanoma patients, who will not receive dabrafenib and trametinib concurrently with the GD2-iCAR-PBT infusion, standard therapy includes prior use of BRAF and/or MEK inhibitor for PBS-eligible BRAF-mutant melanoma, combination ipilimumab/nivolumab immunotherapy or pembrolizumab or nivolumab monotherapy. Patients who have not previously tolerated dabrafenib and/or trametinib in the adjuvant or metastatic setting are also eligible. Patients in whom re-induction with ipilimumab or pembrolizumab or nivolumab is contra-indicated because of grade 3 or 4 non-endocrine immune-related adverse events are also eligible;<br>6. Measurable disease by RECIST 1.1;<br>7. Must be able and willing to provide written informed consent;<br>8. Eastern Cooperative Oncology Group Performance Status of 0 or 1;<br>9. Recovered to ≤ Grade 1 from the acute toxic effects of all prior anti-cancer treatment at least a week before entering this study; for prior ipilimumab, nivolumab, or pembrolizumab,<br>10. Life expectancy of ≥ 12 weeks;<br>11. Availability of T-cell product that has met batch release criteria including ≥ 20% expression of GD2-iCAR (by flow cytometry) on the autologous PBT;<br>12. Fertile male patients must use an effective method of contraception during treatment and for 4 months following discontinuation of trametinib in combination with dabrafenib, or for 4 weeks following discontinuation of dabrafenib;

	13. Female patients are eligible to enter and participate in the study if they meet the following inclusion criteria:<br>• Hysterectomised;<br>• Bilateral oophorectomy (ovariectomy), or<br>• Bilateral tubal ligation, or<br>• Post-menopausal (demonstrated total cessation of menses for greater than or equal to 1 year).<br>For females of childbearing potential, the patient must:<br>• Have a negative serum pregnancy test at screening, and a negative urine pregnancy test, prior to dosing at each treatment course.<br>The female patient must also agree to the use of the following contraceptive methods:<br>An intrauterine device (IUD) with a documented failure rate of less than 1% per year;<br>Vasectomized partner who is sterile prior to the patient's entry and is the sole sexual partner for that woman;<br>Double barrier contraception defined as condom with spermicidal jelly, foam, suppository, or film; OR diaphragm with spermicide; OR male condom and diaphragm;<br>Complete abstinence from sexual intercourse where the lifestyle of the patient ensures compliance;<br>Continue these methods of contraception during treatment and for 4 months following discontinuation of trametinib in combination with dabrafenib, or for 4 weeks following discontinuation of dabrafenib.<br>**Exclusion Criteria**<br>1. Evidence of symptomatic CNS lesions as determined by investigator, use of steroids or anti-seizure medications for treatment of brain metastases. Patients with asymptomatic lesions previously irradiated or surgically resected that are radiologically stable are eligible. Patients

BMJ Publishing Group Limited (BMJ) disclaims all liability and responsibility arising from any reliance placed on this supplemental material which has been supplied by the author(s)

	with incidentally found brain metastasis that are asymptomatic and for which no treatment is planned are also eligible;<br>2. Inadequate bone marrow reserve as demonstrated by an absolute neutrophil count ≤ 1.5 x 10^9/L or platelet count ≤ 100 x 10^9/L (cannot be post-transfusion) or hemoglobin < 90 g/L (can be post-transfusion);<br>3. Serum bilirubin > 1.5 times the upper limit of normal;<br>4. In absence of metastases, liver transaminase levels > 2.5 times the upper limit of normal;<br>5. If metastases are evident, liver transaminase levels > 5 times the upper limit of normal will be acceptable;<br>6. Creatinine clearance of ≤ 50mL/min calculated by Cockcroft-Gault;<br>7. Refractory nausea and vomiting, malabsorption, external biliary shunt, or significant bowel resection that would preclude adequate absorption. Patients must be able to swallow tablets;<br>8. Evidence of severe or uncontrolled systemic diseases (e.g., infection requiring treatment with intravenous (IV) antibiotics, unstable or uncompensated respiratory, cardiac [including life threatening arrhythmias], hepatic, or renal disease.<br>9. Unresolved toxicity ≥ CTC Grade 2 from previous anti-cancer therapy except alopecia (if applicable) unless agreed that the patient can be entered after discussion with the Medical Monitor;<br>10. Presence of at least grade 2 peripheral neuropathy;<br>11. Immune checkpoint inhibitor therapy or participation in a trial of an investigational agent within the 30 days prior to day 0;<br>12. Pregnant or breast-feeding females;<br>13. Patients with an active seizure disorder;<br>14. History of congenital long QT syndrome, history or presence of clinically significant ventricular or atrial dysrhythmias ≥ Grade 2 (NCI CTCAE Version 4.0);

Gargett T, *et al. J Immunother Cancer* 2024; 12:e008659. doi: 10.1136/jitc-2023-008659

	15. Patients with a corrected QTc interval of greater than 450 ms (males) and 470 ms (females);<br>16. Evidence of active infection with HIV, hepatitis B, or hepatitis C;<br>17. Immunosuppressive therapy including corticosteroids within four weeks of screening;<br>18. Patients with a history of hypersensitivity reactions to murine protein-containing products, dabrafenib, trametinib, or another BRAF or MEK inhibitor;<br>19. Use of prophylactic low-dose aspirin, NSAIDs, anticoagulants (unless prescribed for venous or arterial thrombo-embolic disease or atrial fibrillation) will be prohibited in patients commencing dabrafenib and trametinib.<br>20. Patients with a tumour in a location where enlargement could cause airway obstruction;<br>21. Any concurrent condition which in the investigator's opinion makes it undesirable for the patient to participate in this trial or which would jeopardise compliance with the protocol.
**Investigational Product**	GD2 chimeric antigen receptor gene-modified peripheral blood T cells (GD2-iCAR-PBT)
**Treatment Regimen; Route of Administration :**	For patients receiving dabrafenib and trametinib, dabrafenib 150 mg bd orally and trametinib 2 mg od orally is given concurrently while patients are administered GD2-iCAR-PBT by IV injection on Day 0.
**Criteria for Evaluation:**	**Gene Transfer data:**<br>Results will be considered positive when the values exceed the detection threshold and negative control values set for each assay: DNA-PCR for transgene and flow cytometry for 1A7^{+} (GD2-iCAR-bearing) cells.<br>**Primary Immunological data:**<br>A response will be considered to be positive (defined as an increase in the post-test mean of more than 2 SD from the pre-test mean) when at least one of the following criteria is met:

Gargett T, *et al. J Immunother Cancer* 2024; 12:e008659. doi: 10.1136/jitc-2023-008659

	a. ELISPOT analysis shows an increase in the number of IFN-gamma producing cells following culture with or without melanoma antigen-specific stimulation when compared with baseline results;<br>b. Tritiated thymidine incorporation shows an increase in proliferation of PBMC cultures with or without melanoma antigen-specific stimulation when compared with baseline results;<br>c. Cell culture supernatants from PBMC cultures with or without melanoma antigen-specific stimulation, show an increase in the secretion of at least one Th1 or Th2 cytokine when compared with baseline results;<br>d. MHC tetramer analysis by flow cytometry shows an increase in the mean percentage of tetramer-positive cells specific for melanoma antigens when compared to baseline results.<br>**Efficacy Measures:**<br>Tumour response will be determined for all patients with measurable lesions, using RECIST 1.1 criteria for tumour response. The assessments will be made at screening and every 9 to 12 weeks after the GD2-iCAR-PBT infusion.
**Statistical Methods:**	**Safety:**<br>Incidence of adverse events and of marked abnormalities of clinical laboratory tests will be summarised by dose/cohort.<br>**Efficacy:**<br>Individual tumour responses (as defined by the RECIST 1.1 criteria) will be listed by dose/cohort.<br>**Immunology and gene transfer data:**<br>Descriptive statistics will be used for immunological data and, in addition, regression analyses may be possible with gene transfer data.

2. TABLE OF CONTENTS

1. PROTOCOL SUMMARY.. 3

2. TABLE OF CONTENTS 16
3. ABBREVIATIONS AND DEFINITIONS OF TERMS 20
4. INTRODUCTION 22
4.1 Background 22
4.1.1 Malignant melanoma 22
4.1.2 Summary of current treatment for metastatic melanoma 22
4.1.3 The molecular classification of advanced melanoma dictates its treatment .. 23
4.1.4 Oncogenic BRAF in Melanoma and Immunological Consequences 24
4.1.5 Oral BRAF Inhibitors are the New Standard of Care for Advanced BRAF Mutant Melanoma 24
4.1.6 Safety Profile of Dabrafenib 25
4.1.7 Mechanisms of Resistance to Dabrafenib 25
4.1.8 Approved Immunotherapy for Metastatic Melanoma 26
4.1.9 Dabrafenib and Ipilimumab Both Control Brain Metastases of Melanoma .. 27
4.1.10 Sequencing of Treatments with Dabrafenib and Ipilimumab 28
4.2 Adoptive Cell Therapy for Melanoma 29
4.2.1 The GD2 Target in Melanoma, Other Solid Tumours, and Their Treatment 29
4.2.2 Chimeric Antigen Receptors (CAR) 31
4.2.3 Improving Adoptive Cell Therapy Using Chimeric Antigen Receptors 32
4.2.4 Rationale for Combining Dabrafenib with Adoptive T Cell Therapy 33
4.2.5 Preliminary Data in Support of This Protocol 34
4.3 CAR-GD2-PBT 35
4.3.1 Description of product 35
4.3.2 Generation of Transduced Peripheral Blood T Cells (the T-cell Product) 36
4.3.2.1 Retroviral Production 36
4.3.3 Summary of preclinical testing 37
4.3.4 Justification of starting dose and dose progression 37
4.4 Potential Risks and Benefits 37
4.4.1 Infusion of Autologous T cells including Chimeric Antigen Receptor-T cells 38
4.4.2 Improving the Safety of Adoptive Cell Therapy Using Chimeric Antigen Receptors 40
4.4.3 General Consequences of Retroviral Transduction 43
4.4.4 Specific Consequences of Transgene Expression 44
4.4.4.3 Potential Benefits 45
5. STUDY OBJECTIVES 47
5.1 Primary Objectives 47
5.2 Secondary Objectives 47
6. STUDY DESIGN 47
6.1 Continued Treatment 49
7. STUDY POPULATION 49
7.1 Inclusion Criteria 49
7.2 Exclusion Criteria 51
7.3 Concomitant Medications and Treatment 52
7.3.1 Excluded Therapy and Potential Interactions with Concomitant Drugs 52
7.4 Dose Limiting Toxicities 54
7.5 Patient Withdrawal Criteria 54
8. TREATMENT OF PATIENTS 55

8.1 Patient Enrolment, Registration, and Assignment to a Treatment 55
8.2 Dose Levels 55
8.3 Dosing Schedules 55
8.4 Site of Administration 55
8.5 Premedication 55
8.6 Cell Administration 55
8.7 Monitoring 56
8.7.1 Preparation of GD2-iCAR-PBT for Injection 56
8.8 Administration of Rimiducid (AP1903) Homodimeriser Drug 56
8.8.1 Instructions for Preparation 56
8.8.2 Instructions for Infusion 56
8.9 Blinding/Unblinding 57
8.10 Concurrent Anti-cancer Chemotherapy 57

9. STUDY ASSESSMENTS 57

9.1 Safety Assessments 57
9.2 Efficacy Assessments 57
9.2.1 Malignant Disease Evaluation 57
9.2.2 Definitions of Response 58
9.2.2.2 Non-target Lesions 59
9.2.3 Symptomatic Deterioration 60
9.3 Evaluation of Patient's Best Overall Response 60
9.3.2 First Documentation of Response 60
9.3.3 Confirmation of Response 60
9.3.4 Duration of Response 61
9.3.5 Methods of Measurement 61
9.4 Dermatology Assessments 62
9.5 Laboratory Assessments 63
9.5.1 Routine Laboratory Investigations 63
9.5.2 Tests of GD2-iCAR-PBT persistence 63
9.5.3 Tests for Human Ant-Mouse Antibodies (HAMA) 63
9.5.4 Safety Testing for Replication Competent Retrovirus (RCR) 63
9.5.5 Immunological Tests 63
9.5.6 Tumour Biopsies 64
9.5.7 Electrocardiogram (ECG) Assessment 64
9.5.8 Multigated acquisition (MUGA) Scan Assessment 65

10. ASSESSMENT SCHEDULE 66

10.1 Screening Procedures and Assessments 69
10.2 Post Study Follow-up Procedures and Assessments 69

11. ADVERSE EVENTS 69

11.1 Safety Parameters 69
11.2 Adverse Events 69
11.3 Adverse Event Reporting 69
11.3.1 Action(s) Taken 71
11.3.2 Definition of Expectedness 72
11.3.3 Definition of Outcome at the Time of Last Observation 72
11.3.4 Follow-up of Patients with an Adverse Event 72
11.4 Serious Adverse Events 72
11.4.1 Serious Adverse Event Reporting 73

Gargett T, *et al. J Immunother Cancer* 2024; 12:e008659. doi: 10.1136/jitc-2023-008659

CONFIDENTIAL

12. STATISTICAL METHODS **74**
12.1 Sample Size Determination 74
12.2 Definition of Analysis Populations 74
12.3 Statistical Analyses 74
12.3.1 Baseline Characteristics 76
12.3.2 Treatment Compliance 76
12.3.3 Safety Analyses 76
12.3.4 Gene Transfer and Immunology Data 76
12.3.5 Efficacy Analyses 77
12.3.6 Data Safety Monitoring 77
13. ETHICAL CONSIDERATIONS AND ADMINISTRATIVE SECTION **78**
13.1 Local regulations / Declaration of Helsinki 78
13.2 Compliance With Protocol and Protocol Revisions 78
13.3 Informed Consent 78
13.4 Records and Reports 79
13.5 Ethics Committee 79
13.6 Study Records 79
13.6.1 Case Report Form Completion 79
13.7 Warnings, Precautions, and Contraindications 80
13.8 Modification of Protocol 80
13.9 Criteria for Termination of the Study 80
14. PUBLICATION POLICY **81**
16. APPENDIX I – CTC V.4.03 .... **95**
17. APPENDIX II – Effects of Dabrafenib on Concomitant Medications **96**
18. APPENDIX III – Hypersensitivity Reactions **99**
19. APPENDIX IV – HIV/Hepatitis Test Discussion Checklist **100**
20. APPENDIX V – Revised Cytokine Release Syndrome (CRS) Grading System **101**

Gargett T, *et al. J Immunother Cancer* 2024; 12:e008659. doi: 10.1136/jitc-2023-008659

3. ABBREVIATIONS AND DEFINITIONS OF TERMS

Abbreviation	Definition
Ab	Antibody
AE	Adverse Event
APC	Antigen Presenting Cell
COX2	Cyclo-oxygenase-2
CRF	Case report form
CRP	C-Reactive Protein
CTCAE	Common Terminology Criteria of Adverse Events
DC	Dendritic Cells
DTIC	Dacarbazine
DLT	Dose Limiting Toxicity
DTH	Delayed Type Hypersensitivity
EBV	Epstein-Barr Virus
FDA	Food and Drug Administration
GCP	Good Clinical Practice
IL-2	Interleukin-2
ICF	Informed consent form
ICH	International Conference on Harmonization
IFN	Interferon
IV	Intravenous
MAPK	Mitogen Activated Protein Kinase
MHC	Major Histocompatibility Complex
MTD	Maximally Tolerated Dose
NCI	National Cancer Institute
PBMC	Peripheral Blood Mononuclear Cells
PD	Pharmacodynamics
PGE_2	Prostaglandin-E2
PMA	Phorbol 12-myristate 13-acetate

Gargett T, *et al. J Immunother Cancer* 2024; 12:e008659. doi: 10.1136/jitc-2023-008659

SAE	Serious adverse event
SC	Subcutaneous
SDS-PAGE	Sodium Dodecyl Sulphate-Poly Acrylamide Gel Electrophoresis
TNF	Tumour Necrosis Factor
TGFβ	Transforming Growth Factor Beta
VEGF	Vascular Endothelial Growth Factor
V_H	Variable Heavy Chain

Gargett T, *et al. J Immunother Cancer* 2024; 12:e008659. doi: 10.1136/jitc-2023-008659

4. INTRODUCTION

4.1 Background

4.1.1 Malignant melanoma

Malignant melanoma arises from mutations in the melanin producing cells, the melanocytes, which are found mostly in the skin. Consequently, malignant melanoma is usually cutaneous in origin although it may also arise in non-cutaneous sites such as the central nervous system including the eye, aerodigestive and urogenital tracts, and anus.

Excluding non-melanoma skin cancer, melanoma is the fourth most commonly diagnosed cancer in Australia, which has the world’s highest incidence and mortality rate. Melanoma is usually curable surgically in its earliest stages, which is reflected in the 5-year relative survival rate of 92%. Melanoma carries a lifetime risk of approximately 1 in 17. Its incidence is rising in Caucasian populations at a growth rate that is second only to prostate cancer. Over 10,000 new cases occur annually in Australia and, as the ninth most lethal cancer, melanoma is projected to cause over 1,400 deaths per year by 2010 (1). In Australia, cutaneous melanoma is the commonest cancer in the 15-24 year age group (2), and the commonest cancer among males aged 30 to 51 and females aged 17 to 33 (1). Mean age at diagnosis is around 50 years, which is 10-15 years earlier than the commoner diagnoses of prostate, bowel, and lung cancer.

Melanoma prognosis relates to the tumour (T), node (N), and metastasis (M) stage of the disease. Biological aggressiveness and metastatic potential of melanoma is related directly to lesion thickness measured in millimetres, and also to lesion ulceration, the number of lymph nodes involved, and distant organ spread. The 15-year survival rates for localised disease (stages 1 and II) exceed 50% but fall to 30% for stage III disease (nodal involvement), and to less than 2% for stage IV disease (systemic involvement).

4.1.2 Summary of current treatment for metastatic melanoma

Over 2011-2013, the first of the small-molecule class of selective BRAF inhibitors was approved in Australia, Europe, and USA. The striking clinical results will be discussed in more detail below. In Australia, the case for Pharmaceutical Benefits Schedule (PBS) listing for first-line treatment of advanced melanoma with the first-in-class BRAF inhibitor, vemurafenib, was submitted in July 2012 to the Commonwealth Pharmaceutical Benefits Advisory Committee (PBAC). Unfortunately, this and subsequent applications were deferred because of demonstrated lack of pharmaco-economic benefit. Subsequently, vemurafenib’s manufacturer, Roche, withdrew vemurafenib from further consideration by PBAC although vemurafenib remains TGA-approved. Over the same period, the first in an entirely different therapeutic class of anti-melanoma agent, the immune checkpoint inhibitory monoclonal antibody (mAb), ipilimumab (Yervoy®), was approved in Australia (TGA), Europe, and USA, for the second-line treatment of advanced melanoma. The case for PBS listing of ipilimumab was resubmitted to PBAC in July 2012 and ipilimumab was finally PBS-listed a year later.

For unresectable stage III and IV melanoma in Australia, medical treatment is first decided on knowing the BRAF mutation status of the patient's melanoma. If a patient's melanoma has a V600 *BRAF* gene mutation then standard treatment is the BRAF inhibitor, dabrafenib (Tafinlar®). Dabrafenib was listed on the PBS on 1 December 2013 for the following indication: Patients with

Gargett T, *et al. J Immunother Cancer* 2024; 12:e008659. doi: 10.1136/jitc-2023-008659

WHO performance status of 2 or less who have unresectable stage III or stage IV malignant melanoma positive for a BRAF V600 mutation. The condition must not have been treated previously with PBS subsidised therapy and must be used as the sole PBS-subsidised therapy for the condition.

In February 2014, the TGA approved the combination of dabrafenib and the MEK inhibitor, trametinib (Mekinist®) for unresectable stage III or stage IV malignant melanoma positive for a BRAF V600 mutation, and subsequently this combination treatment became listed on the PBS. Hence, combination dabrafenib-trametinib is considered one of the standard of care first-line treatments for BRAF mutation positive melanoma in Australia.

PBS-listed ipilimumab is the standard treatment for Australian patients with unresectable stage III and IV melanoma without a V600 *BRAF* gene mutation. The PBS listing of ipilimumab is for monotherapy of unresectable stage III or stage IV malignant melanoma. This PBS listing of ipilimumab varies from the TGA indication, which is for the treatment of patients with unresectable or metastatic melanoma who have failed or are intolerant to prior therapy. Consequently, the PBS listing of ipilimumab permits first as well as later line use of ipilimumab. Re-induction treatment with ipilimumab is permitted on the PBS if it used as a monotherapy. Patient must have progressive disease after achieving an initial objective response to the most recent course of ipilimumab treatment (induction or re-induction). Again, the treatment must not exceed a total of 4 doses at a maximum dose of 3 mg per kg every 3 weeks. An initial objective response to treatment is defined as either: (i) sustained stable disease of greater than or equal to 3 months duration measured from at least 2 weeks after the date of completion of the most recent course of ipilimumab; or (ii) a partial or complete response.

Nevertheless, the PBS listing of dabrafenib and trametinib constrains its PBS use to the first-line setting and, consequently, precludes the first-line PBS use of ipilimumab, pembrolizumab, or the combination of ipilimumab and nivolumab. For patients who fail dabrafenib and trametinib, there are no randomised controlled clinical data to specify the standard of care after BRAF inhibitor failure. Indeed, in the pivotal second-line trial of ipilimumab (3), which lead to its TGA and PBS approval, BRAF inhibitors were not available as treatment. In Australia, the available and commonly used treatments after failure of BRAF inhibitor therapy are PBS-listed ipilimumab, pembrolizumab, or the combination of ipilimumab and nivolumab. Sometimes, depending on clinical circumstances, fotemustine or dacarbazine chemotherapy may be considered. As will be discussed in section 4.1.11, there are significant risks of rapid progression after BRAF inhibitor failure, which provide a rationale for testing combinations of BRAF inhibitor therapy and immunotherapy to mitigate the risk of disease progression.

4.1.3 The molecular classification of advanced melanoma dictates its treatment

The molecular classification of tumours is rapidly altering the cancer therapy paradigm and nowhere more dramatically than in malignant melanoma. For example, up to 4% and 60% of melanoma samples in total contain activating mutations of KIT (4) or the RAS-regulated kinase BRAF (5), respectively. Most BRAF mutations are of the V600E type (5), and in an almost mutually exclusive manner with the less common activating mutations of NRAS (6), activate the Mitogen Activated Protein Kinase (MAPK) signalling pathway. Activation of this pathway is more common in melanomas that have arisen from intermittently sun-exposed skin (4). In contrast, mutations and/or copy number increases of KIT were found in 28-39% of melanomas found in mucosal or acral regions or on chronically sun-damaged skin (4). Genetic lesions such as amplification, translocation, or other activating mutations of a key gene convert a signal transduction pathway to the dominant driver of

cancer cell proliferation and survival; a cellular state described as oncogene addiction (7). As a corollary, oncogene addiction makes the cancer cell acutely susceptible to pathway blockade using signal transduction inhibitors, which usually abrogate kinase-mediated activation.

4.1.4 Oncogenic BRAF in Melanoma and Immunological Consequences

Vemurafinib and dabrafenib are small-molecule ATP-competitive and highly selective inhibitors of the oncogenic $BRAF^{V600}$ mutant serine-threonine kinase. This mutation is found in about 40% of advanced melanoma cases and causes constitutive activation of the MAPK signalling pathway, which terminates in phosphorylation of ERK (5). Of crucial importance for immunotherapeutic approaches to treatment of BRAF-mutant melanoma, oncogenic BRAF signalling promotes immune evasion by enhancing secretion of the pro-tumorigenic factors, TGFβ and COX-2 (8) (9). Oncogenic V600E BRAF mutation promoted the *in vitro* production of the immunosuppressive factors IL-10, VEGF, and IL-6 from melanoma cells, and this secretion was reduced after inhibition of the oncogene. The culture supernatant from BRAF mutant melanoma cells impaired the secretion of inflammatory cytokines by dendritic cells, which are the critical cell type for initiating anti-melanoma T-cell responses (10). Factors such as TGFβ and IL-10 help to induce regulatory T (Treg) cells, which may play an immunosuppressive role in the tumour microenvironment (11).

4.1.5 Oral BRAF Inhibitors are the New Standard of Care for Advanced BRAF Mutant Melanoma

The first clinical investigation of a selective BRAF inhibitor was performed with vemurafenib. In a phase 1 study, vemurafenib demonstrated mild and manageable toxicity (12) and most patients (24/32) had tumour responses. The phase 1 study determined that the maximum tolerated dose was 960mg bd, which became the recommended phase 2 dose. Through pharmacokinetic/pharmacodynamic correlations, this study also demonstrated that the area under the curve (AUC) values found for doses of 720mg bd and 960mg bd were associated with at least 90% inhibition of phosphoERK in tumour biopsies and tumour regression.

In the BRIM3 phase 3 registration trial of 675 patients with previously untreated, metastatic melanoma containing the V600E BRAF mutation, patients were randomized to standard dacarbazine *versus* vemurafinib. The co-primary study endpoints of superior progression-free survival (PFS) and overall survival (OS) were met (13). At 6 months, OS was 84% (95% confidence interval [CI], 78 to 89) in the vemurafenib group and 64% (95% CI, 56 to 73) in the dacarbazine group. In the interim analysis for OS and final analysis for PFS, vemurafenib was associated with a relative reduction of 63% in the risk of death and of 74% in the risk of either death or disease progression, as compared with dacarbazine ($P<0.001$ for both comparisons).

The clinical activity of dabrafenib is almost indistinguishable from that of vemurafenib. In the BREAK2 phase 2 study (14) of 92 mostly pre-treated metastatic melanoma patients, the confirmed objective response rate (ORR) was 59%. In BREAK2, while a confirmed partial response (PR) was observed for 13% of 16 patients with V600K *BRAF*-mutant melanoma, the median PFS and median OS were 4.5 months and 12.9 months (cf. 6.3 months and 13.1 months for the 76 patients with V600E *BRAF*-mutant melanoma), respectively. These results indicate that patients with V600K *BRAF*-mutant melanoma may obtain a less marked and sustained response to dabrafenib than patients with V600E *BRAF*-mutant melanoma. Furthermore, it is estimated that BRAF V600R mutations comprise 5-7% of all BRAF mutations, and are also sensitive to BRAF inhibition. Five objective responses were observed in six assessable metastatic melanoma patients with the BRAF V600R mutation (15).

Gargett T, *et al. J Immunother Cancer* 2024; 12:e008659. doi: 10.1136/jitc-2023-008659

In the BREAK3 phase 3 study (16) of patients with untreated, advanced *BRAF*-mutant melanoma, the median progression-free survival of dabrafenib-treated patients was 5.1 months (cf. 2.7 months in the dacarbazine arm). The updated median OS (mOS) data from BREAK3 showed mOS of 18.2 months for dabrafenib-treated patients vs 15.6 months for dacarbazine-treated patients. In contrast, in the BRIM3 registration trial of vemurafenib, in which the comparator arm was also dacarbazine, mOS of vemurafenib-treated patients was 13.6 months (cf. 10months in the dacarbazine arm) (17). The longer survival in dabrafenib-treated patients may be due to the more consistent application of the clinical practice of treatment beyond progression in which isolated or symptomatically progressing lesions are treated with a local therapy before dabrafenib treatment is resumed (18).

4.1.6 Safety Profile of Dabrafenib

Dabrafenib was well tolerated with 3% of patients discontinuing dabrafenib in the BREAK3 study. Compared to rates of vemurafenib dose modification (resulting from toxicity) of 45% and 38% in the BRIM2 phase 2 and BRIM3 phase 3 studies of vemurafenib, respectively, the rates of dose modification for dabrafenib in the BREAK2 phase 2 and BREAK3 phase 3 studies were 22% and 28%, respectively. In patients receiving dabrafenib on the BREAK3 study, the most common adverse events (AEs) were cutaneous (hyperkeratosis, papillomas, palmar-plantar erythrodysaesthesia [PPE]), pyrexia, fatigue, headache, and arthralgia. One case of grade 4 hyperkeratosis was reported and grade 3 AEs were uncommon (SCC 4%; pyrexia 3%; fatigue 2%; PPE 2%; arthralgia 1%; hyperkeratosis, 1%; thrombocytopenia 1%; neutropenia 1%). Mandatory reporting of SCCs as grade 3 was required but all were simply excised and did not require dose modification or interruption. Photosensitivity with dabrafenib was rarely seen.

4.1.7 Mechanisms of Resistance to Dabrafenib

Although the BREAK2 and BREAK3 results show that most tumour responses are rapid and most patients achieve tumor regression, the disease persists and most patients relapse. *In vitro* studies supported by studies of tumour biopsies at progression in the presence of drug have demonstrated acquisition of drug resistance despite the retention of the oncogenic BRAF mutation by virtually all tumours. The mechanisms of acquired resistance are both MAPK-dependent and MAPK-independent. The most common mechanism (in approximately 70% of cases), however, is reactivation of MAPK signalling *via* lesions such as BRAF gene amplification, truncated splice variants of BRAF, activating mutations in NRAS lying upstream of BRAF, as well as overexpression of other MAPK pathway enzymes (19) (20). 'Oncogene bypass' mechanisms such as increased extracellular signalling through receptor tyrosine kinases (RTKs) and overexpression of RTKs are also very important (21) (22) (23) (24) (25). Irrespective of the mechanism of resistance, approaches that combine oral BRAF inhibitor with other, non-cross-resistant, therapeutics are urgently required.

The most clinically successful approach to managing the emergence of drug-resistant clones thus far has been further suppression of the MAPK pathway by the combination of a BRAF inhibitor and a MEK inhibitor. The double-blind, placebo-controlled, COMBI-D study was published recently. In this study, 423 treatment-naïve patients with unresectable stage IIIC or stage IV melanoma with a *BRAF* V600E or V600K mutation were randomly allocated to receive the combination of dabrafenib (150 mg orally twice daily) and the MEK inhibitor, trametinib (2 mg orally once daily), or dabrafenib and placebo. Among other factors, patients were stratified according serum LDH status (normal or elevated). The treatment arms were balanced with respect to serum LDH status with approximately

Gargett T, *et al. J Immunother Cancer* 2024; 12:e008659. doi: 10.1136/jitc-2023-008659

one-third of patients having an elevated serum LDH. The primary endpoint of the study was PFS, and an interim survival analysis was performed at 6 months.

The median PFS was 9.3 months in the dabrafenib–trametinib group and 8.8 months in the dabrafenib only group (HR for progression or death in the dabrafenib–trametinib group, 0.75; 95% CI, 0.57-0.99; p = 0.03). In patients with an elevated serum LDH, median PFS was 7.1 months in the dabrafenib–trametinib group compared to 3.8 months in the dabrafenib-only group (HR for disease progression or death, 0.64; 95% CI, 0.42-0.95). The disease control rate (CR + PR+ SD) was at least 92% and 85% in the combination therapy and monotherapy groups, respectively.

The interim OS rate at 6 months was 93% in the dabrafenib–trametinib group and 85% in the dabrafenib only group (HR for death, 0.63; 95% CI, 0.42-0.94; p = 0.02), and median survival had not been reached in either group. Post-hoc subgroup analysis seemed to show that patients with the poorer prognostic feature of elevated serum LDH derived greater benefit from dabrafenib-trametinib than patients overall. In the subgroup with elevated serum LDH at the time of the interim survival analysis, patients receiving dabrafenib–trametinib compared to those receiving dabrafenib alone, had a 52% relative reduction in the risk of death (HR for death, 0.48; 95% CI, 0.29-0.80). In this subgroup, the median survival among patients receiving combination therapy was 13.7 months compared to 8.9 months among those receiving monotherapy (26). However, GSK announced *via* the company website on 6 February 2015 that, compared to dabrafenib monotherapy, the combination therapy demonstrated a statistically significant reduction in the risk of death (HR for death 0.71, 95% CI, 0.55-0.92, p = 0.011).

Nevertheless, recent data indicate that the predominant mechanism of resistance to combination therapy is still MAPK pathway reactivation (27) (28). This result further indicates the importance for ongoing clinical investigation of combinations of MAPK pathway inhibitors with other, non-cross-resistant, therapeutics such as immunotherapy.

4.1.8 Approved Immunotherapy for Metastatic Melanoma

The monoclonal antibody (mAb), ipilimumab (Yervoy®), is a PBS-approved treatment for patients with unresectable or metastatic melanoma. This mAb binds specifically to CTLA4, which is a T cell surface molecule that is expressed after T cell activation. Ipilimumab blocks the interaction of CTLA4 with CD80 and CD86 molecules, which are expressed on the surface of antigen presenting cells. The binding of CD80 or CD86 to CTLA4 terminates T cell activation and clonal expansion and therefore contributes to T cell homeostasis. Because ipilimumab blocks the interaction of CTLA4 with CD80 or CD86, it abrogates this negative feedback signalling in T cells and thus potentiates T cell activation and proliferation. Ipilimumab is the first in a class of therapeutic agents called immune checkpoint inhibitors.

Two phase 3 randomised controlled trials have been conducted and both show a similar survival advantage for use of ipilimumab. In the first trial of pre-treated patients with metastatic melanoma, MDX-020, ipilimumab was compared to a gp100 vaccine, which was considered a relatively inactive control arm. A landmark survival analysis at 2 years showed that 23.5% of patients receiving ipilimumab alone were alive compared to only 13.7% of patients who did not receive ipilimumab (3). In this study, subgroup analysis for OS showed that the hazard ratio for ipilimumab vs control was significant only in patients with normal serum LDH levels. In a retrospective study of 166 Dutch metastatic melanoma patients treated with ipilimumab, a multivariate analysis demonstrated that baseline serum LDH was the strongest predictive factor for OS. This finding was validated in an

independent British cohort of 64 patients. In the Dutch cohort, median OS was 14.7 months for normal LDH vs 3.7 months for elevated LDH (29). Hence, a baseline normal serum LDH, which is also a better prognostic factor in many other cancers, favours use of other T-cell-mediated immunotherapy such as CAR T-cell therapy in patients with metastatic melanoma.

In the second trial of treatment-naïve patients, MDX-024, ipilimumab and dacarbazine was compared to dacarbazine alone. At 2 years, 28.5% of patients receiving ipilimumab were alive compared to 17.9% of patients receiving dacarbazine (30).

Anti-PD1 mAbs represent a second-generation class of immune checkpoint inhibitors available on the PBS for the treatment of melanoma patients who have BRAF mutation negative tumours, or who have failed BRAF/MEK inhibitor combination therapy, or who are intolerant of BRAF/MEK inhibitor combination therapy. Two therapeutic molecules are available: pembrolizumab (Merck, Sharp and Dome) and nivolumab (Bristol-Myers-Squibb). Compared to objective response rates of 10-15% for ipilimumab monotherapy, response rates of up to 44% for anti-PD1 antibody monotherapy have been reported. In particular, pembrolizumab has demonstrated superior overall survival compared to ipilimumab (31). More recently, combination immunotherapy using ipilimumab and nivolumab has been PBS listed for patients with BRAF mutation negative melanoma and has demonstrated superior overall survival compared to ipilimumab monotherapy(32).

4.1.9 Dabrafenib and Ipilimumab Both Control Brain Metastases of Melanoma

Brain metastases are a common feature of melanoma. Cerebral metastases are present at diagnosis in 20% of patients, occur during its disease course in almost 50% of patients, and are found in up to 75% of patients at autopsy (33) (34). Hence, cerebral metastases must be managed as effectively as extra-cranial disease for any systemic treatment to have a favourable impact on survival of metastatic melanoma patients. The median survival of patients with cerebral metastases of melanoma has been 4 months. As a treatment for cerebral metastases, whole brain radiotherapy has not been shown to extend life (34).

The BRIM2, BRIM3, BREAK2, and BREAK 3 trials largely excluded patients with cerebral metastases of melanoma. However, the phase 1 trial of dabrafenib demonstrated activity in the brain (35). Indeed, the inclusion criteria for the phase 4 expanded access program of vemurafenib (MO25515) included patients with asymptomatic untreated cerebral metastases as well as stable treated cerebral metastases (36).

However, the activity of two systemic treatments against brain metastases was formally investigated in separate phase 2 studies of ipilimumab (34) and dabrafenib (33). In the phase 2 ipilimumab study, of 51 patients in cohort A who had asymptomatic cerebral metastases of melanoma and who were not receiving any corticosteroids, 18% exhibited disease control (complete response, partial response, or stable disease) at 12 weeks after commencement of ipilimumab, 24% had disease control in the brain, and 27% had disease control outside of the brain. Of 21 patients in cohort B who had symptomatic cerebral metastases and who were receiving corticosteroids, 5% had disease control at 12 weeks after commencement of ipilimumab, 10% had disease control in the brain, and 5% had disease control outside of the brain. Ipilimumab was given as four 3-weekly doses of 10 mg/kg. Median OS for patients in cohorts A and B was 7.0 and 3.7 months, respectively.

Importantly, for the CARPETS study, these data demonstrate the potential of anti-melanoma lymphocytes, which result from the activity of ipilimumab, to control cerebral metastases, and

Gargett T, *et al. J Immunother Cancer* 2024; 12:e008659. doi: 10.1136/jitc-2023-008659

demonstrate that ipilimumab may provide useful treatment for cerebral metastases of melanoma, particularly if they are asymptomatic and small (34).

Strikingly, as reported in the BREAK-MB study, the anti-melanoma activity of dabrafenib in cerebral metastatic disease mirrors its effects on extra-cranial disease (33). Patients with V600E or V600K BRAF-mutant melanoma and at least one asymptomatic brain metastasis (≥ 5 mm and ≤ 40 mm in diameter) were enrolled into either cohort A (had not received previous local treatment for brain metastases) or cohort B (had progressive brain metastases after previous local treatments). Patients received dabrafenib 150 mg twice daily and the study end point was overall intracranial response in patients with V600E BRAF-mutant melanoma.

Of a total of 172 BREAK-MB patients, 139 (81%) had V600E BRAF-mutant melanoma. In the patients with V600E BRAF-mutant melanoma, intra-cranial disease control (complete response, partial response, stable disease) rates were 81.1% and 89.2% in cohorts A and B, respectively. Similarly, overall disease control rates (intra-cranial and extra-cranial) were 79.7% and 83.1% in cohorts A and B, respectively. PFS in such patients was 16.1 and 16.6 weeks in cohorts A and B, respectively. OS in such patients was 33.1 and 31.4 weeks in cohorts A and B, respectively.

In patients with V600K BRAF-mutant melanoma, intra-cranial disease control rates were 33.3% and 50.0% in cohorts A and B, respectively. Similarly, overall disease control rates (intra-cranial and extra-cranial) were 46.7% and 50.0% in cohorts A and B, respectively. PFS in such patients was 8.1 and 15.9 weeks in cohorts A and B, respectively. OS in such patients was 16.3 and 21.9 weeks in cohorts A and B, respectively.

Importantly, for the CARPETS study, the BREAK-MB data provided two other significant results: (i) serum lactate dehydrogenase (LDH) concentration at study entry was the most discriminating prognostic factor with increased LDH predicting lower response (both overall intra-cranial response and overall response) and shorter median PFS and OS in both cohorts; and (ii) the median PFS for patients with V600E BRAF-mutant melanoma was longer than 16 weeks irrespective of whether patients had had previous local treatment for brain metastases, and that OS was greater than 31 weeks (33).

Hence, these data support the proposed design of the CARPETS study by including: (i) patients with brain metastases in a clinical protocol of a new investigational agent for melanoma (GD2-iCAR-PBT); (ii) melanoma patients with normal serum LDH; (iii) melanoma patients with previously treated, stable brain metastases or asymptomatic brain metastases.

4.1.10 Sequencing of Treatments with Dabrafenib and Ipilimumab

As described in section 4.1.3, the current PBS listings of dabrafenib and ipilimumab do not allow for publicly reimbursed use of ipilimumab before dabrafenib. Both prospective and retrospective clinical data indicate that a significant risk for rapid disease progression following BRAF inhibitor failure exists. Rapid progression of a significant proportion of patients who failed BRAF inhibitor therapy has been documented in several of the prospective vemurafenib clinical trials. Death occurred within 28 days of vemurafenib cessation in 41% and 52% of patients in the phase 2 BRIM2 (37) and phase 3 BRIM3 (38) studies, respectively. In the phase 4 Expanded Access Program of vemurafenib, death within 60 days after the last dose of vemurafenib occurred in 887 (28%) of 3222 patients, mainly because of disease progression (36).

Similarly, in retrospective analyses, rapid disease progression was observed in significant proportions of patients who had prior BRAF inhibitor therapy. In the Italian expanded access program of ipilimumab in 848 melanoma patients, 23% of patients did not receive a third dose of ipilimumab, and of 54 patients, who had previously received BRAF inhibitor therapy, 41% did not continue beyond the second ipilimumab administration (39). In another study of 193 patients who discontinued BRAF inhibitor therapy, median time to death was 3.0 months. Forty of these patients subsequently received ipilimumab but only 50% completed 4 doses, and PFS and OS were 2.7 months and 5.0 months, respectively. No responses to ipilimumab were observed after BRAF inhibitor therapy (40).

In summary, up to 50% of patients with BRAF-mutant melanoma do not stand to benefit from ipilimumab, which is available on the PBS only as a second-line treatment in the event of BRAF inhibitor failure, because the disease will progress too rapid to permit delivery of more than one or two doses, which are too few to have efficacy. These data justify clinical investigations of the combination of BRAF inhibitor therapy and immunotherapy.

4.2 Adoptive Cell Therapy for Melanoma

Evidence has long existed for immune system involvement in the natural history and treatment melanoma. Although occurring at a rate of <1%, spontaneous regression of melanoma occurs more frequently than with other malignancies (41). Moreover, the natural history of melanoma is sometimes marked by an indolent and unpredictable course. The presence of tumour infiltrating lymphocytes (TIL) is a favourable prognostic factor in both primary cutaneous melanoma (42) and regional node metastases (43). Spontaneous albeit ineffective anti-melanoma T cell responses are detected in the peripheral blood of metastatic melanoma patients (44), and may be induced by melanoma antigen-specific vaccines.

T cells that are to be used for adoptive T cell therapy of melanoma may derive from the *ex vivo* expansion of either TIL or peripheral blood T cells (PBT) responding to stimulation with specific melanoma antigens. In either case, the effector T cells bear the native T cell receptor (TCR). Alternatively, *ex vivo*-expanded PBT can be transduced with retroviral vectors that express a high-affinity TCRαβ or a chimeric antigen receptor (CAR) as described below. In which case, the genetically engineered receptor re-directs the effector T cells to melanoma cells (45).

The effector T cells used in TIL therapy were used to clone their cognate melanoma regression antigens, which were mainly normal tissue differentiation antigens involved in melanin pigment formation such as MART-1/MelanA, tyrosinase, and gp100 (46). Many of these same antigens are spontaneously recognised as autoantigens in the sera of melanoma patients (47). A recent review of melanoma adoptive T cell therapy from Rosenberg's group at NCI indicates impressive results. The response rates increased as the extent of lympho-depletion intensified. Of 25 metastatic melanoma patients who received myeloablative conditioning with fludarabine/cyclophosphamide and total body irradiation (12 Gy) followed by peripheral blood stem cell rescue and high-dose IL-2 concurrent with adoptive T cell therapy, the objective response rate was 72% (48). However, these clinical studies were not randomised and lacked intent-to-treat analyses. Hence, it is not clear how much this response rate owes to patient selection, particularly because patients whose melanomas have TIL also have a better prognosis.

4.2.1 The GD2 Target in Melanoma, Other Solid Tumours, and Their Treatment

GD2 is expressed on the cell surface of many types of paediatric, adolescent and young adult (AYA) as well as adult solid tumours. These include the embryonal tumours of neuroblastoma, diffuse

intrinsic pontine glioma, retinoblastoma, medulloblastoma and rhabdomyosarcoma, Ewing sarcoma, osteosarcoma and the soft tissue sarcomas such as the spindle cell sarcomas [pleomorphic undifferentiated sarcoma (previously known as malignant fibrous histiocytoma), leiomyosarcoma, fibrosarcoma], liposarcoma, synovial sarcoma, and desmoplastic small round cell tumour (DRSCT) as well as glioblastoma, small cell lung cancer, melanoma and breast cancer (49).

GD2 is generally expressed at high levels on neuroblastoma. It is also expressed at intermediate density on many melanoma cells (50) (51). Melanoma GD2 expression is significantly upregulated after transformation of melanocytes (52) and is associated with proliferation, migration and metastatic potential of melanoma (53). GD2 is poorly expressed or absent from most normal tissue. GD2 may be a good choice of target for CAR gene-modified T cells, since its expression is highly tissue restricted. Natural or vaccine-induced antibodies to gangliosides in melanoma patients have been correlated with improved disease relapse-free survival (54). Treatment of neuroblastoma or osteosarcoma patients with anti-GD2 mAb has been associated with partial or complete remission of disease (55) (56). Our colleagues at Baylor College of Medicine, Houston, Texas, USA, demonstrated that the overexpression of GD2 by human primary melanoma cells allows these cells to be targeted *in vitro* and *in vivo* by GD2 CAR-expressing lymphocytes (57).

In the following two paragraphs, we report human tumour immunohistochemistical (IHC) studies using GD2-specific antibodies. In this paragraph, we discuss IHC data from studies with the GD2-specific mAb, 3F8. For sarcomas and melanoma, the mean patient age, the number of primary and recurrent cases together the percentage of cases positive by GD2 immunohistochemistry (IHC) is given specifically for osteosarcoma (15.5 years; 9 primary and 10 recurrent; 84%), Ewing sarcoma (17.8 years; 9 primary and 6 recurrent; 40%), rhabdomyosarcoma (10.4 years; 10 primary and 8 recurrent; 50%), DRSCT (19.1 years; 20 primary; 10%) and melanoma (20 primary; 75%). The highest and most uniform level of expression of GD2 was on neuroblastoma followed by osteosarcoma, and the intensity of GD2 expression was heterogeneous on the other tumour types (58). In earlier IHC studies using 3F8, the rates of GD2 immunoreactivity among patients with following soft tissue sarcomas were: liposarcoma 100% (11 of 11), fibrosarcoma 100% (6 of 6), malignant fibrous histiocytoma 86% (6 of 7), leiomyosarcoma 40% (4 of 10), synovial sarcoma 86% (6 of 7), rhabdomyosarcoma 100% (5 of 5), and spindle cell sarcoma 80% (4 of 5) (59). GD2 immunoreactivity was 88% (15 of 17) among patients with primary and metastatic osteosarcoma (60) and 70% (32 of 46) among paediatric and AYA patients with DSRCT (61). In an imaging study using the radiolabelled 3F8 antibody, sites of disease were successfully identified in all 12 of the SCLC patients studied (62).

In this paragraph, we report human tumour immunohistochemistical (IHC) studies using other GD2-specific antibodies including the GD2-specific mAb, 14g2a, from which the scFv employed in the CARPETS study derives. Using 14g2a, the rates of GD2 immunoreactivity were 86% (12 of 14) among patients with Ewing sarcoma (63), and 100% (15 of 15) among patients with uterine leiomyosarcoma (64). Using a polyclonal GD2-specific antibody, GD2 expression was enriched among the tumours of patients with triple-negative breast cancer (TNBC) with an immunoreactivity rate of 67% (27 of 40) (65). The non-switched IgG3 version of 14g2a, 14.18, produced strong immunoreactivity with tissues and cell lines of SCLC (66).

Finally, we provide a clinical update on the use of the GD2-specific monoclonal antibody (mAb), dinutuximab, which is an IgG1 human/mouse chimeric switch variant of the murine mAb, 14G2a.

Gargett T, *et al. J Immunother Cancer* 2024; 12:e008659. doi: 10.1136/jitc-2023-008659

Dinutuximab induces antibody-dependent cell-mediated cytotoxicity (ADCC) and complement-dependent cytotoxicity (CDC). In 2015, the US FDA approved use of dinutuximab in combination with granulocyte macrophage colony-stimulating factor, interleukin-2 and 13-cis retinoic acid in the treatment of paediatric patients with high-risk neuroblastoma who achieve at least partial response to prior first-line multi-agent, multimodality therapy (67) (68). At RAH, we participated in the DISTINCT Study, which is in follow-up phase from end 2018. DISTINCT is a Two-Part, Open-Label, Randomized, Phase II/III Study of Dinutuximab and Irinotecan versus Irinotecan for Second Line Treatment of Subjects with Relapsed or Refractory Small Cell Lung Cancer (ClinicalTrials.gov Identifier: NCT03098030). Peripheral neurotoxicity is known side effect of dinutuximab, which is probably related to the Fc domain of the antibody and which has never been observed with GD2-CAR-T cell therapy using the same 14g2a antigen binding domain. The CAR does not contain a Fc domain, which may explain the lack of evident neurotoxicity with GD2-CAR-T cell therapy.

4.2.2 Chimeric Antigen Receptors (CAR)

Chimeric receptors are generated first by joining the heavy and light chain variable regions of a monoclonal antibody with a linker to form a single-chain Fv (scFv) molecule. This scFv is then attached to the transmembrane and cytoplasmic portion of a T-cell signalling endodomain *via* a flexible hinge region. Engagement of the extracellular scFv of the chimeric receptor results in tyrosine phosphorylation of immune-receptor activation motifs present in the cytoplasmic domain, initiating T cell signalling to the nucleus.

Human T lymphocytes genetically engineered to express these recombinant receptor genes have exhibited specific lysis via the perforin/granzyme pathways, as well as cytokine secretion upon exposure to tumour cells expressing the cognate target antigen (69). Engagement of single T cell or Fc receptor chains suffices to induce cellular activation and proliferation (70-73). Adoptively transferred chimeric receptor-transduced cells were protective in murine tumour models (74-76).

CAR-transduced T cells have numerous advantages over immunotherapies based on monoclonal antibodies or T lymphocytes alone. Since there is no need to select and expand tumour-specific antigens from scanty precursors, large populations of antigen-redirected T lymphocytes can be obtained in a matter of weeks. Moreover, CAR are MHC-unrestricted, so that tumour escape by down-regulation of HLA class I molecules or defects in antigen processing are bypassed. Finally, since both $CD4^+$ and $CD8^+$ T cells can express the same CAR, T helper as well as T cytotoxic functions are directed against tumour cells. The presence of chimeric TCR mediated effector function may be more likely to produce tumour cell lysis than humoral immune responses alone. The perforin/granzyme killing mechanism may be effective against cells that are relatively resistant to antibody and complement, while cytokine secretion upon T cell activation by tumour antigen recruits additional components of the immune system, amplifying the anti-tumour immune response. Furthermore, unlike intact antibodies, T cells can migrate through microvascular walls, extravasate and penetrate the core of solid tumours to exert their cytolytic activity. Finally, a single T lymphocyte can sequentially kill a multiplicity of target cells.

There is little doubt from clinical studies that T cells genetically modified with first-generation CARζ chimeric antigen receptors rapidly lose their function and may disappear from the circulation. The principal problem appears to be that scFv expressing T cells rapidly return to a resting state. Both *in vitro* and *in vivo* studies of T cells stimulated by antigen through their conventional receptors has indicated that in the absence of continuing co-stimulation and of growth factors (as in most tumour

directed killing), there is a progressive decrease in proliferation and loss of cytotoxic T cell activity (77) (78).

4.2.3 Improving Adoptive Cell Therapy Using Chimeric Antigen Receptors

However, the co-stimulation required for survival, expansion and cytotoxic activity may be provided to PBT by adding the required signalling domains to the CAR construct (79) (57). In a comparative study of ζ, CD28-ζ and CD28-OX40-ζ endodomains of GD2-specific CAR responding to GD2-expressing neuroblastoma cells, the CD28-OX40-ζ construct optimised the proliferation, survival and cytolytic capacity of transgenic T cells (79). The sustained cytotoxic functions of the CD28-OX40-ζ containing GD2-iCAR was manifest as significant anti-tumour efficacy of GD2-iCAR gene-modified polyclonal human T cells in a xenograft metastatic model of GD2-expressing human melanoma (57). Importantly, as observed for another CAR target (80), melanoma cell killing by these GD2-iCAR gene-modified T cells occurred even when GD2 expression was relatively low (57). As illustrated in Figure 1, the 14g2a scFv sequence was cloned in the SFG retroviral backbone in frame with a spacer derived from the human IgG1 hinge region, followed by CD28 and OX40 signalling domains and then the ζ-chain of the TCR/CD3 complex to form the GD2-iCAR construct as described (79).

To allow tracking of transduced PBT by real-time PCR, a modification has been introduced into the non-coding region of the retroviral vector. A short (13 bp) oligonucleotide is inserted after the stop codon of the CAR and before the 3' LTR (Figure 1). By measuring the level of the transgene in peripheral blood, our American collaborators can estimate the overall kinetics of gene-modified T-cell survival and determine whether expansion or persistence occurs *in vivo*. Currently, they can regularly detect a single *neo*-marked cell in 50,000 cells by real-time PCR analysis, which has been sufficient to track T cells for up to 6 years in their ongoing EBV-prophylaxis study. Furthermore, the 14g2a scFv can be detected by an anti-idiotypic mAb, 1A7 (81), so that gene-modified T cells may be detectable in PBMC samples using flow cytometry. Hence, 25 mL of patient blood will be taken preinfusion, and at various intervals subsequently.

CONFIDENTIAL

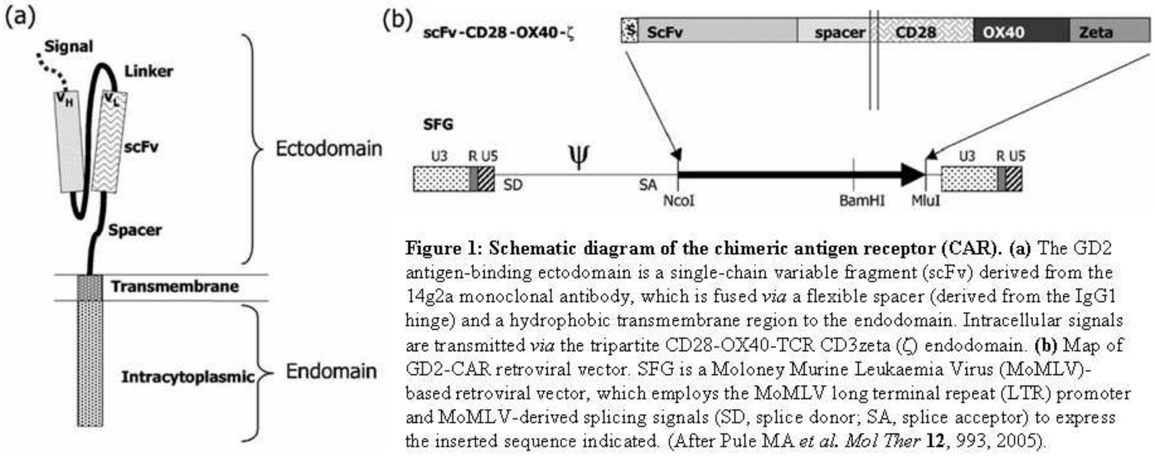

Figure 1: Schematic diagram of the chimeric antigen receptor (CAR). (a) The GD2 antigen-binding ectodomain is a single-chain variable fragment (scFv) derived from the 14g2a monoclonal antibody, which is fused *via* a flexible spacer (derived from the IgG1 hinge) and a hydrophobic transmembrane region to the endodomain. Intracellular signals are transmitted *via* the tripartite CD28-OX40-TCR CD3zeta (ζ) endodomain. **(b)** Map of GD2-CAR retroviral vector. SFG is a Moloney Murine Leukaemia Virus (MoMLV)-based retroviral vector, which employs the MoMLV long terminal repeat (LTR) promoter and MoMLV-derived splicing signals (SD, splice donor; SA, splice acceptor) to express the inserted sequence indicated. (After Pule MA *et al. Mol Ther* **12**, 993, 2005).

4.2.4 Rationale for Combining Dabrafenib with Adoptive T Cell Therapy

The recent successful control of advanced melanoma with new targeted agents such as vemurafinib and dabrafenib has advanced the concept of using this approach as a platform to develop potentially curative treatments for this lethal disease. BRAF inhibitors enable reliable, rapid, but not sustained, control of disease. Inevitably, therapeutic resistance results in relapse. Conversely, lymphocyte-mediated immunotherapies including IL-2, ipilimumab and adoptive cellular therapies are slow to produce tumor remissions which, while occurring at a lower rate, may nonetheless be durable in a proportion of responders. We now propose to combine adoptive transfer of autologous lymphocytes engineered to be reactive to the melanoma-associated antigen GD2 with the BRAF inhibitor, dabrafenib. The established activity of dabrafenib in melanoma and of GD2-specific T cells in human studies of neuroblastoma and murine studies of melanoma coupled with their non-overlapping mechanisms of action, lack of cross-resistance, and complementary clinical activity (speed versus duration of control) provide a compelling rationale for investigating the combination of BRAF inhibitors and adoptive T-cell therapy. Several recent findings strengthen the rationale for combining a BRAF inhibitor, and even combined BRAF/MEK therapy, with adoptive T cell therapy.

First, melanoma tumour biopsies, which were taken approximately two weeks after the commencement of BRAF inhibitor therapy, revealed that BRAF inhibition produced an intratumoral infiltrate of $CD4^+$ T cells and granzyme-B-expressing (activated) $CD8^+$ T cells (82). An *in vitro* study showed that a specific inhibitor of oncogenic V600E BRAF in melanoma cell lines and tumor digests resulted in increased expression of melanoma differentiation antigens and was associated with improved recognition by antigen-specific T lymphocytes (83). In another study, melanoma biopsies taken from patients 14 days after commencing vemurafenib alone or dabrafenib and trametinib showed both increased melanoma differentiation antigen expression and an influx of $CD8^+$ T cells. These changes were also associated with a decrease in immunosuppressive cytokines (IL6 and IL8) and an increase in markers of T-cell cytotoxicity. At the time of progression on BRAF inhibitor alone, a decrease in melanoma antigen expression and CD8 T-cell infiltrate was observed but this was reversed with combined BRAF and MEK inhibition (84). Furthermore, selective BRAF inhibition has the additional pro-immunogenic effect of reducing the frequency of circulating myeloid-derived suppressor cells (85).

Gargett T, *et al. J Immunother Cancer* 2024; 12:e008659. doi: 10.1136/jitc-2023-008659

Second, pharmacological BRAF inhibition did not impair T cell activation, proliferation or function, and did not produce any deleterious effect on pre-existing systemic immunity or on the *de novo* generation of tumour-specific T cells (83) (86) (87). In addition, serum cytokine levels were not altered in dabrafenib-treated melanoma patients (88), and lymphopenia was observed in vemurafenib-treated rather than in dabrafenib-treated melanoma patients (89).

Third, the combination of MAPK inhibitor therapy and immunotherapy has produced unexpected findings. A phase 1 trial of ipilimumab and vemurafenib was closed early due to hepatotoxicity although the mechanism was not clear (90). In an ongoing phase 1 trial of ipilimumab with or without dabrafenib and/or trametinib in patients with metastatic melanoma (NCT01940809), an interim finding of several cases of severe colitis has prevented further recruitment to the ipilimumab and dabrafenib/trametinib arm while recruitment to the ipilimumab and dabrafenib arm continues (91). Finally, a recently reported preclinical study of adoptive TCR T-cell therapy (ACT) in combination with dabrafenib/trametinib treatment in a murine model of melanoma indicated that while the dabrafenib/trametinib combination inhibited T cell function *in vitro*, it enhanced ACT *in vivo via* T cell tumour infiltration, cytotoxicity and cytokine production (92). In section 4.2.5, we briefly describe the favourable interaction between GD2-iCAR T cells and dabrafenib *in vitro*.

Therefore, it can be reasoned that reactivation of MAPK signalling during the acquisition of resistance to BRAF inhibitor therapy will have biological consequences including immune evasion, which resemble those observed in cells driven by unrestrained oncogenic BRAF signalling (8) (9) (10). Some *in vivo* support for this proposition derives from the observation that an intratumoral T-cell infiltrate was absent at the time of melanoma progression (82) (84).

We hypothesise that concurrent inhibition of oncogenic BRAF with adoptive transfer of autologous GD2-iCAR-T cells may facilitate entry of the T cells into a tumor microenvironment in which pro-immunogenic melanocyte differentiation antigens and inflammatory cytokines are expressed, dendritic cells are functionally competent, and immunosuppressive cytokines and Treg cells are reduced or absent. Our unpublished preliminary data indicate that the functional capacity of GD2-iCAR-T cells may be intact in the presence of some immunosuppressive factors such as regulatory T cells, COX2-generated PGE_2, and TGFβ (included in Pharmaceutical & Pharmacological Data document).

4.2.5 Preliminary Data in Support of This Protocol

To examine the feasibility of conducting this study at RAH Cancer Centre, 44 formalin-fixed and paraffin-embedded (FFPE) samples of metastatic melanoma held in the archives at SA Pathology were analysed by immunohistochemistry (IHC) in the laboratory of Dr Andrew Ruszkiewicz using our adaptation of a published method (93). These samples were collected between 2002-2009 from patients with a mean age of 64 years and M:F ratio of 1.6, and thus were representative of the proposed target population of this clinical study. Applying a stringent criterion of >50% of tumour cells staining strongly, 18/44 (41%) of samples were positive. These results are consistent with an earlier study in which 50% of metastatic melanoma samples (*n*=83) express GD2 using the 14.18 mAb (51). If a less stringent criterion was applied of >50% of tumour cells staining weakly then an additional 20/44 (45%) samples were positive.

We have recently generated data using V600E BRAF mutant melanoma cell lines to show that *in vitro* expression of GD2 is highly dependent on culture conditions including the kind of the medium and plating density. These data suggest that *in vivo* expression of GD2 will depend on cues in the

Gargett T, *et al. J Immunother Cancer* 2024; 12:e008659. doi: 10.1136/jitc-2023-008659

tumour microenvironment. Moreover, other *in vitro* data indicate that GD2 expression levels on V600E BRAF mutant melanoma cell lines can be varied by various endoplasmic reticulum (ER) stressors, which include pharmacological BRAF inhibition. Therefore, we believe that even limited expression of GD2 by the V600E/K/R/D BRAF-mutant melanoma cells of patients receiving dabrafenib may be therapeutically relevant because GD2 expression levels may be dynamically modulated *in vivo*. Hence, we have chosen a low threshold of GD2 expression, ≥ 10% tumour cells as GD2 positive, as a trial entry criterion. Furthermore, we will investigate melanoma expression of GD2 prospectively in any tumour biopsies obtained from patients during the course of the study.

Our own *in vitro* studies investigated the effects of approved MAPK pathway-targeted therapies for melanoma (vemurafenib, dabrafenib and trametinib) on the viability, activation, proliferation and CTL activity of GD2-iCAR T cells and non-transduced T cells. While all of these drugs inhibited the stimulation of the T cells at high concentrations, only vemurafenib inhibited T cells at concentrations equivalent to the plasma concentrations reported in treated patients. The combination of dabrafenib and trametinib also resulted in inhibition of T-cell effector functions at some therapeutic-like concentrations, but dabrafenib itself had little adverse effect on CAR T-cell function at these concentrations (94). In the clinical setting, this degree of inhibition may be countered by increased antigen expression, an improved tumour microenvironment (85) and increased lymphocyte infiltration into the tumour (84) (82), and by the greater range of co-stimulatory, cell adhesion, and chemokine and cytokine stimuli available *in vivo.* As we found that GD2-iCAR T cells stimulated directly *via* their CAR may be more resistant to kinase inhibition, we believe that the GD2-iCAR T cells remain strong candidates for therapy in combination with dabrafenib. Furthermore, data generated from preclinical tumour models of the combination either of dabrafenib/trametinib and adoptive T-cell therapy (95) or trametinib and anti-PD1 or anti-CTLA4 monoclonal antibodies (96) show that *in vivo* trametinib-containing regimens can promote anti-tumour activity that is significantly greater than any of the treatments individually (95) (96).

4.3 CAR-GD2-PBT

4.3.1 Description of product

The product is an autologous T-cell product that is generated *ex vivo* under code of Good Manufacturing Practice (cGMP) conditions in the SA Pathology Therapeutic Products Facility. It comprises peripheral blood T cells (PBT) that have been genetically modified to express a chimeric antigen receptor (CAR). The CAR consists of an ectodomain, which uses the 14g2a antibody fragment to target melanoma-expressed GD2. The ectodomain located on the outside of the cell is fused on the inside the cell to a tripartite T-cell activating endodomain, which comprises CD28, OX40 and the CD3ζ chain. Thus, upon binding of the ectodomain to melanoma-expressed GD2, the genetically modified PBT become activated, and kill GD2-expressing melanoma cell targets *in vitro* and *in vivo* (57). In addition to the CAR, the expression cassette encoded by the retroviral vector includes inducible caspase 9 (iCasp9), which is fused to the CAR by a cleavable 2A linker sequence (Fig. 2). Consequently, the hybrid iCasp9 protein can be dimerised by the external application of a bioinert small-molecule drug, rimiducid (AP1903), resulting in caspase 9 activation and the rapid induction of apoptosis in the genetically modified T cell (97).

Gargett T, *et al. J Immunother Cancer* 2024; 12:e008659. doi: 10.1136/jitc-2023-008659

4.3.2 Generation of Transduced Peripheral Blood T Cells (the T-cell Product)

Peripheral blood (180mL) will be collected from each patient and used to generate the cellular components listed below, for infectious disease testing required to generate a local Certificate of Analysis for the T-cell product, and for HLA typing as autologous identity testing of the T-cell product. All steps in the preparation of the T-cell product will be conducted in accordance with the Standard Operating Procedures (SOP's) of the Cell and Gene Therapy Centre, Baylor College of Medicine, Houston, Texas, USA.

4.3.2.1 Retroviral Production

First, a well-characterised retroviral producer line that makes the vector encoding the GD2-iCAR is used to create a clinical grade retrovirus Master Cell Bank (MCB). The MCB contains approximately 100-200 vials at $1x10^7$ cells per vial, and is used to create a working cell bank from which clinical grade retroviral supernatant is produced. Subsequently, sufficient clinical grade retrovirus will be produced for the study patients. The harvested supernatants will be filtered, aliquoted, and rapidly frozen and stored at –80°C. Retroviral producer cells and retroviral supernatants are tested at each step of this production process for Replication Competent Retrovirus (RCR) and contamination by bacteria, fungi, mycoplasma, and viruses. The Cell and Gene Therapy Center at Baylor College of Medicine, Houston, Texas, USA issues the Certificates of Analysis, which document these test results, for the MCB, vialed producer cells, end-of-production producer cells, and retroviral supernatants. These documents will be filed in an updated IB.

4.3.2.2 PBT Transduction

PBT are stimulated *in vitro* with OKT3 (anti-CD3 mAb) and IL-7 and IL-15. On Day 3, PBT are transduced using Retronectin (98). Plates are coated with recombinant Fibronectin fragment CH-296 (Retronectin™, Takara, Madison, WI). The transducing viruses are attached to Retronectin by incubating producer supernatant in coated plates onto which the cells are transferred.

4.3.2.3 *Ex vivo* expansion

After transduction, transgenic PBT are further expanded *in vitro* with OKT3 and fed with IL-7 and IL-15 every 3 days to reach the sufficient number of cells as per protocol (usually 15 ± 3 days).

4.3.2.4 Characterisation of T-cell Product and Freezing

After transduction a small number cells will be removed. Transduction efficiency will be determined by flow cytometry. Cytotoxicity assays against GD2-expressing and GD2-negative cell lines will be done to check the function of the transgenic receptors (57).

The line will be checked for identity, phenotype and microbiological culture and cryopreserved prior to administration according to SOPs of the Cell and Gene Therapy Centre, Baylor College of Medicine, Houston, Texas, USA.

4.3.2.5 Certificate of Analysis for the Autologous T-cell Product

Before the autologous T-cell product can be infused into the patient, certain batch release criteria will have been met (as listed in the Table below). Fulfilment of the batch release criteria will be documented with a Certificate of Analysis for each T-cell product generated in the SA Pathology Therapeutic Products Facility.

Test	*Specification*
Viability (Trypan Blue Dye exclusion)	> 70% Viable
Endotoxin (LAL assay)	≤ 5.0 EU/kg/hour
Bacterial sterility final product	Negative at 10 days
Fungal sterility final product	Negative at 10 days
Cytotoxicity Assays	≥20% killing of the $GD2^+$ LAN1 neuroblastoma cell line at 10:1 effector:target ratio at 12 hours concurrent with <10% killing of autologous PHA blasts at 10:1 effector:target ratio.
Transduction	≥20% positive of assigned vector by 1A7 mAb-based immunophenotyping
Suicide gene function	After 48h treatment with 20nM rimiducid, <10% residual GD2-$iCAR^+$ cells (MFI >10^2)
RCR testing	Negative real-time PCR assay for GALV envelope sequences

Table 4.3.2

4.3.3 Summary of preclinical testing

The construction, expression and biological characterisation of the GD2-iCAR retroviral vector has been published. In a comparative study of ζ, CD28-ζ and CD28-OX40-ζ endodomains of GD2-specific CAR responding to GD2-expressing neuroblastoma cells, the CD28-OX40-ζ construct optimised the proliferation, survival and cytolytic capacity of transgenic T cells (79). The sustained cytotoxic functions of the CD28-OX40-ζ containing GD2-iCAR was manifest as significant anti-tumour efficacy of GD2-iCAR gene-modified polyclonal human T cells in a xenograft metastatic model of GD2-expressing human melanoma (57). For additional information including evidence of intact proliferative and cytolytic functions of GD2-iCAR-PBT, which include the iCasp9 suicide gene in the retroviral expression cassette, in the presence of the vemurafenib analogue, PLX4720, as well a demonstration of suicide gene function, please refer to the Pharmaceutical & Pharmacological Data document.

4.3.4 Justification of starting dose and dose progression

The starting dose and dose levels will be those employed in the published phase I study of a related first-generation vector (GD2-iCARζ), which was used to genetically modify PBT in patients with advanced neuroblastoma (99), and in the ongoing GRAIN study (Clinicaltrials.gov: NCT01822652) in the same patient population. The proposed dosing schedule is justified because no adverse events were observed in these studies.

4.4 Potential Risks and Benefits

Potential toxicities may be categorised as those related to infusion of T cells, general consequences of retroviral transduction, and specific consequences of the transgene expressed. As with any immunotherapeutic agent there is a possibility of severe immediate hypersensitivity reactions including anaphylaxis. The first dose of GD2-iCAR-PBT will be administered intravenously in a phase 1 clinical trial unit called PARC, which is adjacent to the RAH Intensive Care Unit where full resuscitation facilities and physicians experienced in the management of anaphylaxis will be

available. Patients will be observed in this ward for 24 hours and it is unlikely that serious immediate hypersensitivity reactions will present after that time.

4.4.1 Infusion of Autologous T cells including Chimeric Antigen Receptor-T cells

In many previous studies, larger numbers of *ex-vivo* cultured T cells, which have been activated *ex vivo*, have been infused with no adverse effects (100) (101). In previous adoptive immunotherapy protocols of our US collaborators, 100 patients have been treated in which EBV-specific cytotoxic T lymphocytes (CTL) were administered after bone marrow transplantation (102) (103) (104). No immediate complications, which could be attributed to the CTL infusions, were observed. The main complications have been an inflammatory reaction in a patient with bulky EBV positive disease at the time of CTL administration and mild inflammatory reactions in three other patients (102). Thirteen patients have received autologous EBV-specific CTL for EBV-positive Hodgkin's lymphoma (105). One patient developed a transient cachexia syndrome after a third dose of CTL. Another patient had erosion of the mediastinal tumour through bronchus and pulmonary artery two months post-CTL, but gene marked CTL were not found at the site of the tumour erosion. A third patient had back pain prior to receiving his first dose of CTL and developed cord compression due to progressive disease one week later. Over 30 patients with Hodgkin disease or non-Hodgkin lymphoma (NHL) have received LMP1- or LMP1/2-specific CTL with no significant adverse effects (106). In addition, 33 patients have been treated on a study using EBV specific CTL for nasopharyngeal cancer with no toxicity attributed to CTL (107) (108).

A number of studies using T cells transduced with CAR have also been reported:

(i) Using a first-generation GD2-iCARζ vector, which is a precursor of the retrovector proposed in this application, 19 patients with advanced neuroblastoma and who were EBV seropositive were treated with a single injection of an equal number of GD2-iCARζ gene-modified EBV-CTL (CAR-CTL) and GD2 CARζ gene-modified PBT (CAR-PBT), for a total dose of $2x10^7$ to $2x10^8$ cells. Each cell type had been genetically modified with retrovectors, which could be distinguished by PCR because of vector-specific 13-basepair sequences in non-coding DNA of each vector. No adverse events (AE) were attributable to the genetically modified T cells in the 11 patients in 24 months of follow-up (99) (109).

This phase I study generated interesting results. CAR-CTL persisted longer in blood of patients than did CAR-PBT. The apparent persistence of CAR-CTL was hypothesised to result from preferential *in vivo* survival of CAR-CTL because endogenous antigen presenting cells present latent EBV antigen to stimulate the native TCR and to provide 'physiological' co-stimulation. Conversely, the relatively poor survival of CAR-PBT is suggested to result from a lack of *in vivo* co-stimulation and is consistent with results from other studies (110) (99). Five of 10 advanced neuroblastoma patients with evaluable tumours had evidence of tumour necrosis or regressions including two sustained complete responses. Two of 9 patients with extensive disease developed pain at the site of tumour and fever 10-14 days after infusion, associated with biopsy proven tumour necrosis. All patients rapidly responded to analgesia.

Interestingly, needle biopsies of three of the necrotic tumours yielded T cell infiltrates but not PCR signals for the gene-modified T cells, which suggested that the observed tumour responses might have resulted from indirect mechanisms of cytotoxicity. Based on the slow but progressive development of the complete responses and the persistence only of

Gargett T, *et al. J Immunother Cancer* 2024; 12:e008659. doi: 10.1136/jitc-2023-008659

functional CAR-CTL and not CAR-PBT, the hypothesis was developed that CAR-CTL rather than CAR-PBT initiated the tumour cell killing and provoked the immune cascade that recruited immune effector cells other than those actually infused (99) (and personal communications).

Nevertheless, the long-term clinical and immunological consequences of the GD2-iCAR T cell infusions in 19 patients with high risk neuroblastoma; 8 in remission at infusion, 11 with active disease, was reported recently (109). Three of 11 patients with active disease achieved complete remission, and persistence of either CAR-PBT or CAR-CTL beyond 6 weeks was associated with superior clinical outcome. Persistence for up to 192 weeks for CAR-PBT and 96 weeks for CAR-CTL was observed, and duration of persistence was highly concordant with the percentage of $CD4^+$ cells and central memory cells ($CD45RO^+CD62L^+$) in the infused product (109). These important observations in one type of $GD2^+$ tumour warrant further clinical exploration of the effects of modified GD2-iCAR-PBT in subjects with $GD2^+$ melanoma.

(ii) Six patients with neuroblastoma received T cells expressing a CAR specific for CD171 (111). T-cell infusions were well tolerated with no over toxicities. CD171-CAR T cells could be detected for up to 7 days post-infusion in patients with bulky disease and up to 42 days post-infusion in patients with limited disease burden.

(iii) Seven patients with non-Hodgkin lymphoma or mantle cell lymphoma received T cells expressing a CAR specific for CD20 (112). No dose limiting toxicity was observed and of the 7 treated patients, 2 maintained a previous complete response, 1 achieved a partial response, and 4 had stable disease.

(iv) Three patients with advanced and treatment-refractory chronic lymphocytic leukaemia (CLL) were treated with autologous T cells expressing a CAR specific for CD19 (CART19) (113) (114). The CAR was encoded in a lentiviral vector and had a bipartite T-cell endodomain comprising 4-1BB (CD137) and CD3ζ signalling moieties. After preparative bendamustine chemotherapy, patients were infused with three consecutive divided doses of CART19 cells ranging from 1.5×10^5 cells/kg to 1.6×10^7 cells/kg. Approximately 2 weeks after the infusions, all patients developed high fevers and rigors and two patients had hypotension at the same time as a cytokine storm was documented (114). In addition, one patient developed tumour lysis syndrome with transient renal impairment approximately 3 weeks post-infusion (113). Two patients were reported to have been hospitalized during these acute episodes (113) (114). The CART19 cells expanded 1,000- to 10,000-fold during the period the clinical events were documented and persistent memory CART19 cells were detected 6 months later. Two patients had a durable complete response and one patient had a partial response (113) (114).

(v) Two recent deaths have been reported in relation to the use of tripartite chimeric antigen receptors.

The first was a female patient who had colon cancer metastatic to the lungs and liver and was refractory to multiple standard treatments. She was treated at the U.S. National Cancer Institute under the supervision of Prof Steven Rosenberg. After non-myeloablative conditioning, she received a 10^{10} dose of peripheral blood lymphocytes (PBL), which had been transduced with a retroviral vector that expressed a tripartite chimeric antigen receptor (CAR). The CAR was based on the humanised and ERBB2 (HER2)-specific monoclonal

antibody (mAb) trastuzumab (HERCEPTIN) and contained CD28, 4-1BB, and CD3ζ signalling moieties. Seventy-nine percent of $CD3^+$ PBL expressed the CAR. Within 15 minutes after cell infusion, the patient experienced respiratory distress, and displayed a dramatic pulmonary infiltrate on chest X-ray. She was intubated and, despite intensive medical intervention, she died 5 days after treatment. Serum samples after cell infusion showed marked increases in interferon-gamma (IFNγ), granulocyte macrophage-colony stimulating factor (GM-CSF), tumour necrosis factor (TNF), interleukin-6 (IL-6), and IL-10, consistent with a cytokine storm.

The authors speculated that the large number of administered cells localised to the lung immediately following infusion and were triggered to release cytokine by the recognition of low levels of ERBB2 on lung epithelial cells (115). However, the patient presumably had ERBB2-expressing pulmonary metastases, and the HERCEPTIN product information (for breast cancer) cautions against use of HERCEPTIN in patients with pulmonary metastases:

“Severe pulmonary events leading to death have been reported rarely with the use of HERCEPTIN in the post-marketing setting. Signs, symptoms and clinical findings include dyspnoea, interstitial lung disease including pulmonary infiltrates, pleural effusions, respiratory distress, non-cardiogenic pulmonary oedema, pulmonary insufficiency, hypoxia, pneumonitis, pulmonary fibrosis and acute respiratory distress syndrome and pneumonia. Interstitial pneumonitis has been reported as a rare but serious complication in clinical trials of HERCEPTIN in localised breast cancer. These events may occur as part of an infusion-related reaction or with a delayed onset. Patients with symptomatic intrinsic lung disease or with extensive tumour involvement of the lungs, resulting in dyspnoea at rest, may be at greater risk of severe reactions.”

The second patient was treated at Memorial Sloan Kettering Cancer Center, New York, NY, USA. That patient had chronic lymphocytic leukaemia (CLL) and received approximately 10^{10} PBL transduced with a retroviral vector, which expressed a tripartite CAR construct directed toward CD19 on CLL cells. The patient was prepared for the infusion with lymphoreductive therapy (cyclophosphamide 1g). Shortly after infusion, the patient deteriorated suddenly with a cytokine storm-like picture and died (116).

These two deaths occurred in circumstances that contrast sharply with those proposed in the current protocol. The vector used in this protocol is related to the vector used in a phase I trial of advanced neuroblastoma. No adverse events were reported in the 11 treated patients (99). In this protocol, no prior myeloablative or lymphoreductive therapy will be used, and the maximum cell dose (10^8) will be 100-fold less than that administered in both of the fatal cases.

4.4.2 Improving the Safety of Adoptive Cell Therapy Using Chimeric Antigen Receptors

The abovementioned reports of clinical toxicities related to the infusion of second or third generation CAR T cells, which incorporate more than just a CD3ζ signalling chain in the T cell endodomain of the CAR, indicate that risks exist for any CAR T cell infusion irrespective of conditioning regimen and the number of cells infused. Hence, to minimise these risks, we have incorporated into the retroviral expression cassette an inducible cellular suicide gene that is expressed in frame with the GD2-iCAR (Fig. 2). The suicide gene is expressed as a chimeric protein comprising the intracellular portion of the human caspase 9 protein, which signals apoptotic cell death, fused to a drug-binding

domain derived from human FK506-binding protein (FKBP). This chimeric protein is quiescent inside cells until administration of the bioinert small-molecule drug, rimiducid (AP1903), which cross-links the FKBP domains, initiating caspase signalling and hence apoptosis.

4.4.2.1 SFG.iCasp9.2A.GD2-iCAR Retroviral Vector

SFG.iCasp9.2A.GD2-iCAR consists of inducible caspase 9 (iCasp9) linked *via* a 'cleavable' 2A-like sequence to the GD2-iCAR (Fig. 2). iCasp9 consists of a human FK506-binding protein (FKBP12; GenBank AH002 818) with an F36V mutation, connected *via* a Ser-Gly-Gly-Gly-Ser linker to human caspase 9 (CASP9; GenBank NM 001229) (117). The F36V mutation increases the binding affinity of FKBP12 to the synthetic homodimeriser, rimiducid (AP1903) (118). The caspase recruitment domain (CARD) has been deleted from the human caspase 9 sequence because its physiological function has been replaced by FKBP12, and its removal increases transgene expression and function (117). The 2A-like sequence encodes an 18 amino acid peptide from *Thosea Asigna* insect virus, which mediates a non-proteolytic ribosomal 'skip' step that simulates protein cleavage and thus enables the production of separate GD2-iCAR and icasp9 translation products (119) (120).

Gargett T, *et al. J Immunother Cancer* 2024; 12:e008659. doi: 10.1136/jitc-2023-008659

CONFIDENTIAL

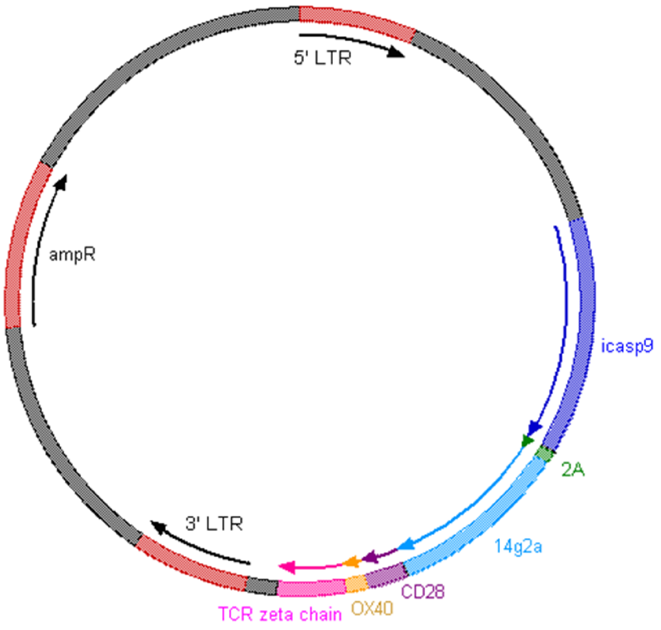

Figure 2: Schematic depiction in Gene Construction Kit (v.2.5) of the genetic map of the SFG.iCasp9.2A.GD2-CAR retroviral vector. **LTR:** long terminal repeat (promoter); **iCasp9:** inducible caspase 9 gene; **2A:** 2A-like ribosomal 'skip' sequence; **14g2a:** ectodomain comprising single chain Fv of anti-GD2 monoclonal antibody; **CD28, OX40, TCR zeta chain:** components of intracellular T cell activating endodomain; **ampR:** ampicillin [illegible]

4.4.2.2 Inducible Caspase 9 (iCasp9) Suicide Gene

Inducible caspase 9 (iCasp9)-mediated suicide is based on conditional dimerisation of pro-apoptotic molecules, which comprise a drug-binding domain joined to human caspase 9 (117). Administration of rimiducid dimerises and activates caspase 9; this activates downstream caspases and leads to apoptosis of both dividing and non-dividing cells within 24 hours. The iCasp9 gene encodes human proteins and is therefore unlikely to be immunogenic (117). The iCasp9 suicide gene system is trademarked CaspaCIDe and is the proprietary technology of Bellicum, Inc. (www.bellicum.com).

4.4.2.3 Safety of Synthetic Homodimeriser, rimiducid

rimiducid is an investigational drug that has not been approved for marketing in any country.

rimiducid was evaluated in a phase I clinical safety study using normal healthy male volunteers. No significant adverse effects were noted when rimiducid was administered over a 0.01mg/kg to 1.0mg/kg dose range (121). The incidence of adverse events was very low following each treatment, with all adverse events being mild in severity. Only one adverse event was considered possibly related to rimiducid. This was an episode of vasodilatation, described as "facial flushing" for one volunteer at the 1.0 mg/kg rimiducid level. This event occurred at 3 minutes after the start of infusion and resolved after 32 minutes' duration. All other adverse events reported during the study were

Gargett T, et al. J Immunother Cancer 2024; 12:e008659. doi: 10.1136/jitc-2023-008659

considered by the investigator to be unrelated or to have improbable relationship to the study drug. These events included chest pain, flu syndrome, halitosis, headache, injection site pain, vasodilatation, increased cough, rhinitis, rash, gum haemorrhage, and ecchymosis (121).

In a clinical trial of 4 children with leukaemia who were given a 2 hour intravenous infusion at the dose proposed in this study, 0.4mg/kg, no rimiducid-related adverse events were observed (97).

Finally, in a phase I study, 18 patients with metastatic prostate cancer received multiple subcutaneous injections with a gene modified dendritic cell vaccine followed 24 hours later by a 2 hour intravenous infusion of rimiducid (0.4mg/kg) resulting in side effects in two patients. In one patient, there was evidence of a cytokine mediated release or hypersensitivity like reaction upon infusion of rimiducid as a result of the activation of the modified dendritic cells. The other patient had urticaria and flushing, which improved with antihistamine administration and allowed administration of further rimiducid infusions.

4.4.2.4 Pharmacology and Pharmacokinetics of the Synthetic Homodimeriser, rimiducid

Our patients would receive 0.4 mg/kg of rimiducid as a 2 h infusion based on published PK data which show maximal plasma concentrations of 10 to 1275ng/mL (equivalent to 7 to 892nM) over the 0.01mg/kg to 1.0mg/kg dose range with plasma levels falling to 18% and 7% of maximum at 0.5 and 2hrs post dose (121). There are no known drug interactions.

In preclinical experiments, efficient function of the iCasp9 suicide gene was shown when it produced >90% apoptosis after treatment with dimeriser. Although transgene expression tends to be down-modulated with time, it is rapidly reversed by T cell activation, which occurs when the gene modified T cells encounter their targets, thus enabling efficient killing of the gene modified T cells with rimiducid (122).

4.4.3 General Consequences of Retroviral Transduction

Retroviral transduction results in new random integration in host cell DNA, which rarely may cause abnormal or uncontrolled proliferation (123). This effect is much more common with replication-competent retrovirus (RCR) where each cell receives multiple integrants (124). We will test the producer line and all batches of supernatant with biological assays of RCR to exclude this possibility. This will be done at the time of production at the Cell and Gene Therapy Center, Baylor College of Medicine, Houston, TX, USA, and absence of RCR is a specification included in the Certificates of Analysis for the producer line and batches of supernatant to be used in this study.

Recently, there has been concern that even a single retroviral integration can contribute to oncogenesis. Fischer's group replaced the missing common gamma chain (γc) in X-SCID patients with *ex-vivo* retroviral transduction of autologous stem cells. Four of 11 treated patients have developed T-acute lymphoblastic leukaemia (ALL). The first two of the cases were associated with a single retroviral integrant at the LMO2 proto-oncogene site. In the latter two cases, one patient had retroviral integrations at the LMO2 and BMI1 proto-oncogene sites and the other patient had a retroviral integrant at the CCND2 proto-oncogene site (125). More recently, 1 of 12 patients in a similar study in England has also developed T cell lymphoproliferation. In 4 of 5 affected X-SCID patients, the insertion site was in the LMO2 proto-oncogene, and it is likely that correction of common-gamma chain deficiency and related immunodeficiency syndromes will represent special cases (126).

Gargett T, *et al. J Immunother Cancer* 2024; 12:e008659. doi: 10.1136/jitc-2023-008659

The γc is a shared component of IL-2, IL-4, IL-7, IL-9 and IL-15 (127). Hence, it is a crucial component T-cell proliferation and thymogenesis. A proliferative advantage is expected for progeny of stem cells with functional γc. In female carriers of X-SCID there is a pattern of non-random X-inactivation in T-cells, B-cells and NK-cells (128). Moreover a patient with X-SCID developed substantial numbers of T-cells following reversion of the mutant allele in a single haemopoietic stem cell (127). High efficiency retroviral transduction of human stem cells is difficult to achieve even with GALV pseudotyping (129). It is likely that in X-SCID patients, few truly pluripotent stem cells were transduced and the stem cell pool expressing the highest level of transgene (due to integration at a transcriptionally active site) then undergo numerous doublings to restore the entire T-cell compartment. Random mutations caused by this supra-physiological proliferation combined with a retroviral integration in a transcriptionally active region led to leukemogenesis (126).

To date more than 200 patients have received genetically modified cells in clinical trials (130) including patients who have treated on the protocols of our US collaborators at Baylor College of Medicine, Houston, Texas – using retrovirally marked autologous marrow (131) or retrovirally marked EBV-specific CTL (104) (103). In none of these patients has malignancy caused by retroviral transduction been reported. However, there remains a finite possibility that the vector could randomly integrate into a site that could lead to leukaemogenesis.

Patients eligible for the current study will have advanced melanoma. In light of this, the natural history and poor prognosis of advanced melanoma and given the entire previous experience with retroviral gene therapy, we feel that the risks of retrovirally induced leukaemogenesis are small and are justified in this patient group.

4.4.4 Specific Consequences of Transgene Expression

4.4.4.1 The GD2 Antigen in Patients

GD2 (Diasialoganglioside $II^3a(NeuAc)_2GgOse_3Cer]$) is a disialoganglioside expressed in tumours of neuroectodermal origin. Gangliosides are sialic-acid (neuraminic acid) containing glycosphingolipids. They consist of a sialated polysaccharide chain linked through a ß-glycosidic linkage to a ceramide moiety, which anchors them into the plasma membrane. They are major constituents of neuronal cell membranes and endoplasmic reticulum. The principal gangliosides of normal mammalian brain are GM1, GD1a, GD1b and GT1a. The GD2 tumour-associated antigen seems to be ideal for Ab-mediated therapy of melanoma, since it is expressed at intermediate density on the majority of melanoma cells, and is poorly expressed or absent from most normal tissue. It is expressed on cerebellum and peripheral nerves. Many clinical studies have been performed using a variety of monoclonal antibodies targeting GD2. Published studies are summarised in table 4.4.1.

Table 4.4.1

Clone	Effects	Reference
L72	8 patients with melanoma were treated. One patient developed complete regression. Side effects were limited to mild erythema.	(132)
3F8	9 patients with melanoma and 8 with neuroblastoma. Four major responses noted. Complement depletion, hypertension and urticaria were noted.	(133)

14g2a	12 patients with melanoma. Reversable peripheral motor neuropathy. Delayed pain syndrome. High HAMA titers in all patients. SIADH also occurred.	(134)
14g2a	5 neuroblastoma patients – 2 partial remissions.	(135)
Ch14.18	9 patients with stage IV neuroblastoma. Disease response in 6/9. Abdominal and joint pain, pruritis, urticaria and optic atrophy occurred.	(136)
Ch14.18	16 patients with metastatic melanoma. No clinical benefit noted. Abdominal pain, hypotension, dysasthesias, atrial fibrillation.	(135)
14g2a	33 pediatric patients with neuroblastoma and GD2 positive osteosarcoma. Response was noted in 9 patients. Side-effects included fever, thrombocytopenia, neutropenia, diarrhea, bronchospasm, hypotension and angioedema.	(137)
Ch14.18	10 pediatric patients with neuroblastoma. Response noted in 5. Toxicities included pain, tachycardia, hypertension, fever, rashes, nephropathy, arthritis and notably mild peripheral paresthesias.	(138)
3F8 coupled to I-131	24 previously untreated patients with neuroblastoma, 75% remain progression-free with a median follow-up of 19 months	(139)

Disease responses were noted. The majority of toxicities seemed to result from infusing murine antibodies into human subjects. Although we will be using variable regions derived from one of these monoclonals (14g2a), these will be integrated into the CAR and expressed on T-cells.

4.4.4.2 Cross-reactivity

GD2 is expressed at low levels on cerebellum and peripheral nerves. Clinical studies using 14g2a murine monoclonal Ab (donor of variable domains for our chimeric receptor) have resulted in reversible neurotoxicity. Patients in these studies developed high titres of human-anti-mouse antibodies (HAMA) and toxicity was believed to be caused by immune precipitation of HAMA-14g2a complexes. Although our chimeric receptor is derived from 14g2a mouse hybridoma only the variable regions are retained and hence only anti-idiotype antibodies can be formed. Since the chimeric receptor is membrane-bound anti-idiotype antibodies will lead to clearance of the transduced cells in the spleen rather than immune-precipitation. Direct neurotoxicity from targeting of GD2 expressing normal tissues by transduced cells is however still possible. In case of this eventuality, and in consultation with the medical monitor, a short intravenous infusion of rimiducid will be administered to induce programmed destruction of gene modified T cells that may be responsible for the toxicity. If this event occurs, it will be defined as a DLT.

4.4.4.3 Potential Benefits

It is unknown whether there will be an improvement of disease symptoms or survival in patients undergoing therapy with GD2-iCAR-PBT. Anti-tumour responses recorded in advanced neuroblastoma patients who received T-cells modified using the related first-generation retroviral

vector (GD2-iCAR-CD3ζ) suggest that anti-tumour responses in metastatic melanoma patients are possible using this tripartite (CD28-OX40-CD3ζ) chimeric antigen receptor. Metastatic melanoma patients who participate in this trial of GD2-iCAR-PBT will provide useful information about the safety and immune effects of this tripartite (CD28-OX40-CD3ζ) chimeric antigen receptor that will guide use of it or its successors in future patients with malignant melanoma.

5. STUDY OBJECTIVES Primary Objectives

The primary objectives of this study will be to determine:

1. The feasibility of preparing T cell products for administration to patients with GD2-positive malignancy
2. The safety profile and dose limiting toxicities of autologous peripheral blood T cells directed to GD2 through their chimeric antigen receptor (GD2-iCAR-PBT as the T cell product) in patients with GD2-positive malignancy

5.2 Secondary Objectives

The secondary objectives of this study are:

1. To assess *in vivo* persistence of infused GD2-iCAR-PBT
2. To assess tumour infiltration by infused GD2-iCAR-PBT
3. To assess bystander anti-melanoma immune effects of BRAF inhibitor therapy and infused GD2-iCAR-PBT
4. To document persistence of anti-tumour effects after the infusion of GD2-iCAR-PBT as measured by partial or complete tumour response or stable disease

6. STUDY DESIGN

This study is an open-label, single ascending dose-escalation study of GD2-iCAR-PBT infusion in patients with GD2-positive melanoma, which is either BRAF mutation negative or BRAF mutation positive (containing a dabrafenib-eligible *BRAF* gene mutation at the V600 codon i.e. V600E/K/R/D), or other GD2-positive malignancies.

Patients who have a Hb ≥ 105g/L and unresectable stage III or IV melanoma, which is either BRAF gene mutation negative or positive (BRAF V600E/K/R/D mutations), or other GD2-positive malignancies, will be asked to consent to the study. Patients with BRAF-mutant melanoma must be eligible to receive dabrafenib and trametinib. As part of the study consent, we will ask patients for permission to test their (i) blood samples for evidence of active infection with human immunodeficiency virus 1 or 2 (HIV1/2), hepatitis B virus (HBV), and hepatitis C virus (HCV), and (ii) archived or fresh tumour tissue samples for GD2 expression. These tests have a turn around time of up to 2 days.

If patients do not have evidence of active infection with HIV1/2, HBV or HCV and have tumour expression of GD2 then patients will be asked to provide a 360mL blood sample for (i) generation of the T-cell product (180mL), (ii) isolation and storage of peripheral blood mononuclear cells (PBMC) for later immunological testing (80mL), (iii) baseline studies and routine haematology and biochemistry (MBA20) tests (100mL).

Patients will then receive dabrafenib at the initial dose of 150 mg bd and trametinib at the initial dose of 2 mg od while their T-cell product is being prepared. As part of disease evaluation on the combination therapy, patients will have CT scans every 9 to 12 weeks.

It is anticipated that it will take 3 to 4 weeks to prepare a qualified autologous T cell product. Patients will receive a single intravenous injection of the previously prepared T-cell product, which is a dose

of GD2-iCAR gene-modified peripheral blood T cells (PBT). The subsequent treatment evaluation period is 6 weeks.

The study will employ a Bayesian dose-finding method called the modified continual reassessment method (mCRM) (140) (141) to determine the safety of one intravenous injection of autologous PBT directed to GD2 through their chimeric antigen receptor (CAR) in patients with GD2-positive metastatic melanoma, which is either BRAF mutation negative or BRAF mutation positive (containing BRAF V600E/K/R/D mutations). The mCRM minimises the number of patients who may be exposed to an ineffective dose. Given the lack of adverse events in the first-in-human study using the related first-generation GD2-CARζ vector (99), and the absence of significant toxicity in four advanced neuroblastoma patients who have received autologous peripheral blood T cells modified using the same GD2-iCAR vector in the completed GRAIN protocol (Clinicaltrials.gov: NCT01822652) (142), and our own favourable safety data concerning the first six melanoma patients enrolled via the CARPETS protocol, the proposed dosing schedule of the GD2-iCAR-T PBT product made under a new set of ex vivo cell manufacturing conditions comprises the following dose levels:

Dose Level 2: 2×10^7 cells/m^2

Dose Level 3: 1×10^8 cells/m^2

Each patient will be followed for 6 weeks after the PBT infusion for evaluation of dose limiting toxicity (DLT). The toxicity will be evaluated by the NCI Common Terminology Criteria for Adverse Events version 4.03. A DLT is an event considered to be primarily related to the PBT infusion if it occurs at any time up to 6 weeks from the PBT infusion and is defined as ≥ CTCAE version 4.0 grade 3 toxicity (not recovered within 5 days); non-haematological toxicities of any duration, which are severe enough and deemed DLT after discussion with the medical monitor; grade 3 hypersensitivity reaction, which did not respond to H1 and H2 blockade, recurred after prophylactic H1 and H2 blockade or any Grade 4 reaction; grade 3 fever (> 40.0˚C ≤ 24 hours), which did not respond to interruption of dabrafenib or of dabrafenib and trametinib or to dose reduction or oral prednisolone (up to 25 mg mane) or any Grade 4 fever (> 40.0˚C > 24 hours) associated with hypotension (SBP < 100 mm Hg); grade 3 chills or flu-like symptoms lasting more than 5 days, which did not respond to interruption of dabrafenib or of dabrafenib and trametinib or to dose reduction or oral prednisolone (up to 25 mg mane) or any Grade 4 reaction associated with hypotension (SBP < 100 mm Hg); and Grade 3 or 4 Cytokine Release Syndrome (CRS) (see Appendix V).

A DLT may trigger the administration of rimiducid, which itself will be defined as a DLT. For example, Grade 3 CRS not responding to standard clinical management with supportive measures, dexamethasone, and tocilizumab, or Grade 4 CRS will trigger the administration of rimiducid. Rimiducid is bioinert small-molecule drug that activates a molecular suicide program in GD2-iCAR-PBT, which results in their immediate destruction and thus may terminate any GD2-iCAR-PBT-related adverse events.

For this trial, the maximum tolerated dose (MTD) is defined to be the dose that causes a DLT in 20% of patients. All patients within a dose cohort should have completed the 6-week window after the PBT infusion for assessment of DLT prior to enrolment of patients into the next recommended dose level.

To facilitate the dose escalation process, there will be a review of the safety data by the Medical Monitor and Principal Investigator who will provide this information to the data safety monitoring board (DSMB) as described in detail in section 12.3.6. The DSMB will provide a written

recommendation on a decision to dose escalate based on protocol-specified criteria and advise the study site accordingly. Copies of the documentation will be retained in the study site file.

The mCRM will employ a cohort of size 2 based on the exponential dose-toxicity model. The fixed prior probabilities of DLT for the three dose levels indicated above were assumed to be 5%, 8% and 25%, respectively. To ensure patient safety, the mCRM starts from the lowest dose level and limits the dose escalation to one dose level at a time. A DLT event in the lower dose cohort will be used to update the dose-toxicity curve. The next patient cohort is assigned to the dose level with an associated probability of DLT closest to the target probability of 20%. This process continues until at least 10 patients have been accrued into the trial or 6 patients have been treated at the current MTD. Depending on patient availability and dose escalation, a maximum of 12 patients will be recruited into this Phase I trial. The final MTD will be the dose with probability closest to the target toxicity rate at these termination points. Since GD2-iCAR-PBT generation for this trial takes 3-4 weeks, we plan to generate T-cell products for up to 15 patients accounting for potential 'drop outs' and change in patients' eligibility status (80% completion rate).

During the study, patient toxicity outcomes will be monitored in real time in order to estimate the dose-toxicity curve and determine the dose level for the next patient cohort using one of the pre-specified dose levels**.** Patients in a cohort can be enrolled concurrently but both patients within a dose cohort should have completed the 6-week window after the PBT infusion for assessment of DLT prior to enrolment of patients into the next recommended dose level. To increase safety, patients enrolled in the same dose cohort will not be treated within the same week. The recommended phase II single agent dose will be based on the findings of the toxicity, immune effects, and activity profile of the patients in this phase I study.

6.1 Continued Treatment

If a patient not receiving dabrafenib or trametinib has a confirmed complete or partial response without evidence of dose-limiting toxicity (DLT) at their subsequent CT scan evaluations, then a written request will be made to the Research Ethics Committee to consider two additional infusions of GD2-iCAR-PBT, if available and if eligibility criteria for administration continue to be met (see **Section 7**). The gene-modified T cells for any additional infusions will have been made and qualified before the initial dose. An additional infusion would be at the previous cell dose and would be administered no less than 6 weeks after the first dose when the initial safety profile is completed. A similar 6-week safety evaluation period would follow the second or third infusion using the same Assessment Schedule (see **Section 10**).

Since virtually all of the patients who receive dabrafenib and trametinib will achieve stabilization or shrinkage of their disease, these patients will not receive further infusions of GD2-iCAR-PBT.

7. STUDY POPULATION

7.1 Inclusion Criteria

7.1.1 At least 18 years old;

7.1.2 Histological diagnosis of small cell lung cancer, triple negative breast cancer, osteosarcoma, Ewing sarcoma, and such soft tissue sarcomas as rhabdomyosarcoma, liposarcoma, fibrosarcoma, synovial sarcoma, pleomorphic undifferentiated sarcoma, and desmoplastic small round cell tumours (DSRCT), or metastatic melanoma

(surgically incurable and unresectable stage III or stage IV; AJCC Cancer Staging Manual, 7th edition, 2010) and ≥10% GD2 positive cells (by an independent pathologist); patients with other malignancies with ≥ 10% GD2 positive cells are also eligible;

7.1.3 Unresectable stage III disease must have confirmation from a surgical oncologist. Melanoma will have V600 *BRAF* gene mutation status determined; V600E, K, R, or D mutations may be eligible for treatment with dabrafenib and trametinib.

7.1.4 Must have failed standard therapy;

7.1.5 For metastatic melanoma patients, who will not receive dabrafenib and trametinib concurrently with the GD2-iCAR-PBT infusion, standard therapy includes prior use of BRAF and/or MEK inhibitor for PBS-eligible BRAF-mutant melanoma as well as combination ipilimumab/nivolumab immunotherapy or pembrolizumab or nivolumab monotherapy. Patients who have not previously tolerated dabrafenib and/or trametinib in the adjuvant or metastatic setting are also eligible. Patients in whom re-induction with ipilimumab or pembrolizumab or nivolumab is contra-indicated because of grade 3 or 4 non-endocrine immune-related adverse events are also eligible;

7.1.6 Measurable disease by RECIST 1.1;

7.1.7 Must be able and willing to provide written informed consent;

7.1.8 Eastern Cooperative Oncology Group Performance Status of 0 or 1;

7.1.9 Recovered to grade 1 from the acute toxic effects of all prior anti-cancer treatment at least a week before entering this study;

7.1.10 Life expectancy of ≥ 12 weeks;

7.1.11 Availability of T-cell product that has met batch release criteria including ≥ 20% expression of GD2-iCAR (by flow cytometry) on the autologous PBT;

7.1.12 Fertile male patients must use an effective method of contraception during treatment and for 4 months following discontinuation of trametinib in combination with dabrafenib, or for 4 weeks following discontinuation of dabrafenib;

7.1.13 Female patients will be eligible for inclusion and participation in this study if any of the following criteria apply:

- Hysterectomised;
- Bilateral oophorectomy (ovariectomy), or
- Bilateral tubal ligation, or
- Post-menopausal (demonstrated total cessation of menses for greater than or equal to 1 year).

For females of childbearing potential, the patient must:

- Have a negative serum pregnancy test at screening, and a negative urine pregnancy test, prior to dosing at each treatment course.

The female patient must also agree to the use of the following contraceptive methods:

An intrauterine device (IUD) with a documented failure rate of less than 1% per year;

Vasectomized partner who is sterile prior to the patient's entry and is the sole sexual partner for that woman;

Double barrier contraception defined as condom with spermicidal jelly, foam, suppository, or film; OR diaphragm with spermicide; OR male condom and diaphragm;

Complete abstinence from sexual intercourse where the lifestyle of the patient ensures compliance;

Continue these methods of contraception during treatment and for 4 months following discontinuation of trametinib in combination with dabrafenib, or for 4 weeks following discontinuation of dabrafenib.

7.2 Exclusion Criteria

A patient will not be eligible for inclusion in this study if any of the following criteria apply:

7.2.1 Evidence of symptomatic CNS lesions as determined by investigator, use of steroids or anti-seizure medications for treatment of brain metastases. Patients with asymptomatic lesions previously irradiated or surgically resected that are radiologically stable are eligible. Patients with incidentally found brain metastasis that are asymptomatic and for which no treatment is planned are also eligible;

7.2.2 Inadequate bone marrow reserve as demonstrated by an absolute neutrophil count ≤1.5 x 10^9/L or platelet count ≤ 100 x 10^9/L (can not be post-transfusion) or hemoglobin <90 g/L (can be post-transfusion);

7.2.3 Serum bilirubin > 1.5 times the upper limit of normal;

7.2.4 In absence of metastases, liver transaminase levels greater than 2.5 times the upper limit of normal;

7.2.5 If metastases are evident, liver transaminase levels greater than 5 times the upper limit of normal will be acceptable;

7.2.6 Creatinine clearance of ≤50mL/min calculated by Cockcroft-Gault;

7.2.7 Refractory nausea and vomiting, malabsorption, external biliary shunt, or significant bowel resection that would preclude adequate absorption. Patients must be able to swallow tablets;

7.2.8 Evidence of severe or uncontrolled systemic diseases (e.g., infection requiring treatment with intravenous (IV) antibiotics, unstable or uncompensated respiratory, cardiac [including life threatening arrhythmias], hepatic, or renal disease.

7.2.9 Unresolved toxicity ≥ CTC Grade 2 from previous anti-cancer therapy except alopecia (if applicable) unless agreed that the patient can be entered after discussion with the Medical Monitor;

7.2.10 Presence of at least grade 2 peripheral neuropathy;

7.2.11 Immune checkpoint inhibitor therapy or participation in a trial of an investigational agent within the 30 days prior to day 0;

7.2.12 Pregnant or breast-feeding females;

7.2.13 Patients with an active seizure disorder;

7.2.14 History of congenital long QT syndrome, history or presence of clinically significant ventricular or atrial dysrhythmias ≥ Grade 2 (NCI CTCAE Version 4.0);

7.2.15 Patients with a corrected QTc interval of greater than 450 ms (males) and 470 ms (females);

7.2.16 Evidence of active infection with HIV, hepatitis B, or hepatitis C;

7.2.17 Immunosuppressive therapy including corticosteroids within four weeks of screening;

7.2.18 Patients with a history of hypersensitivity reactions to murine protein-containing products, dabrafenib, trametinib, or another BRAF or MEK inhibitor;

7.2.19 Use of prophylactic low-dose aspirin, NSAIDs, anticoagulants (unless prescribed for venous or arterial thrombo-embolic disease or atrial fibrillation) will be prohibited in patients commencing dabrafenib and trametinib;

7.2.20 Patients with a tumour in a location where enlargement could cause airway obstruction;

7.2.21 Any concurrent condition which in the investigator's opinion makes it undesirable for the patient to participate in this trial or which would jeopardise compliance with the protocol.

7.3 Concomitant Medications and Treatment

Patients commencing dabrafenib and trametinib can continue with their concomitant medications unless they are precluded medications (Section 7.2 above and see below). Concomitant medications must be fully recorded on the CRF. In addition, any diagnostic, therapeutic, or surgical procedure performed during the study period should be recorded including the date, indication, description of the procedure(s) and any clinical findings.

The following medications and treatments are not allowed while the patient is on the study:

- other anti-cancer therapies;
- concomitant alternative therapies and herbal preparations;
- radiotherapy for the treatment of disease during the study except for palliative limited field radiotherapy for non-target lesions by RECIST assessments. For patients requiring radiotherapy while on study, dabrafenib and trametinib treatment must be interrupted at least 7 days before the procedure and recommenced 3 days after the procedure if oral intake is adequate.

7.3.1 Excluded Therapy and Potential Interactions with Concomitant Drugs

Dabrafenib is mainly metabolised by CYP2C8 and CYP3A4 to form hydroxy-dabrafenib, which is further oxidized *via* CYP3A4 to form carboxy-dabrafenib. Carboxy-dabrafenib can be

Gargett T, *et al. J Immunother Cancer* 2024; 12:e008659. doi: 10.1136/jitc-2023-008659

decarboxylated via a non-enzymatic process to form desmethyl-dabrafenib, which in turn is a substrate of CYP3A4. Carboxy-dabrafenib is excreted in bile and urine, and desmethyl-dabrafenib may also be formed in the gut and reabsorbed. Hydroxy-dabrafenib terminal half-life parallels that of parent with a half-life of 10 hours while the carboxy- and desmethyl-metabolites exhibited longer half-lives (21-22 hours). Both hydroxy- and desmethyl-dabrafenib are likely to contribute to the clinical activity of dabrafenib; while the activity of carboxy-dabrafenib is not likely to be significant.

Concomitant medications are not prohibited during this study except the above therapies. However, medications that are mainly metabolised by CYP450 3A4 and 2C9 enzymes should be used with caution when co-administered with dabrafenib. Drugs that are strong inhibitors or inducers of CYP2C8 or CYP3A4 are likely to increase or decrease, respectively, dabrafenib concentrations. Alternative agents should be considered during administration with dabrafenib when possible. Caution should be used if strong inhibitors (e.g. ketoconazole, nefazodone, clarithromycin, ritonavir, saquinavir, telithromycin, itraconazole, voriconazole, posaconazole, atazanavir) are co-administered with dabrafenib. Co-administration of dabrafenib with potent inducers of CYP2C8 or CYP3A4 (e.g. rifampin, phenytoin, carbamazepine, phenobarbital, St. John's wort (*Hypericum perforatum*) should be avoided.

Appendix II includes a non-exhaustive list of typical examples of CYP2C9 and CYP3A4 substrates and CYP3A4 and CYP2C8 inducers and inhibitors.

<u>Effects of dabrafenib on Concomitant Medications:</u> After 3 days of repeat dosing, dabrafenib induces CYP3A4- and CYP2C9-mediated metabolism and may induce other enzymes including CYP2B6, CYP2C8, CYP2C19, UDP glucuronosyl transferase (UGT) and transporters. The single dose AUC of midazolam (CYP3A4 substrate) and S-warfarin (CYP2C9 substrate) was decreased by 74 and 37%, respectively, with co-administration of dabrafenib.

Co-administration of dabrafenib and drugs, which are affected by the induction of these enzymes or transporters such as hormonal contraceptives, dexamethasone, antiretroviral agents, or immunosuppressants, may result in decreased concentrations and loss of efficacy. Concomitant use of dabrafenib with these drugs should generally be avoided if monitoring for efficacy and dose adjustment is not possible. If CYP2C9 or CYP3A4 substrates must be co-administered with dabrafenib, then the investigator should monitor for signs of reduced benefit of CYP2C9 or CYP3A4 drugs because of a potential decrease in their plasma concentrations. Doses of concomitant CYP1A2 and CYP3A4 drugs, but not the dose of dabrafenib, may be adjusted as necessary to alleviate the effects of drug-drug interactions. Warfarin has a narrow therapeutic index, and when it must be co-administered with dabrafenib, the investigator should assess the risk of bleeding associated with potential increase in warfarin exposure, and consider additional INR (International Normalized Ratio) monitoring. Upon discontinuation of dabrafenib, concentrations of sensitive CYP3A4 substrates may increase and patients should be monitored for toxicity and dosage of these drugs may need to be adjusted. Caution must be exercised and

<u>Effect of Other Medicines on dabrafenib:</u> Co-administration of ketoconazole (CYP3A4 inhibitor) and gemfibrozil (CYP2C8 inhibitor) increased the AUC of dabrafenib by 71 and 47%, respectively. Pharmacokinetic data showed increases in hydroxy- and desmethyl-dabrafenib AUC with ketokonazole (increases of 82 and 68% respectively while a decrease in AUC was noted for carboxy-dabrafenib (decrease of 16%). No clinically relevant changes were noted in the AUC of the metabolites during co-administration with gemfibrozil.

Combination of trametinib with dabrafenib: Co-administration of trametinib 2 mg once daily and dabrafenib 150 mg twice daily resulted in no clinically relevant pharmacokinetic drug interactions.

7.4 Dose Limiting Toxicities

An event will be considered a DLT if it occurs at any time up to 6 weeks from the PBT infusion, is considered to be primarily related to GD2-iCAR-PBT, and meets one of the below criteria despite maximum supportive care provided.

1. CTCAE version 4.0 Grade 3 or higher toxicity that is not recovered within 5 days;

   Some non-haematological toxicities of any duration are severe enough and will be deemed DLT after discussion with the medical monitor
2. Grade 3 hypersensitivity reaction which did not respond to H1 and H2 blockade, recurred after prophylactic H1 and H2 blockade or any Grade 4 reaction.
3. Grade 3 fever (> 40.0˚C ≤ 24 hours), which did not respond to interruption of dabrafenib or of dabrafenib and trametinib, or to dose reduction or oral prednisolone (up to 25 mg mane) or any Grade 4 fever (> 40.0˚C > 24 hours) associated with hypotension (SBP < 100 mm Hg).
4. Grade 3 chills or flu-like symptoms lasting more than 5 days, which did not respond to interruption of dabrafenib or of dabrafenib and trametinib, or to dose reduction or oral prednisolone (up to 25 mg mane) or any Grade 4 reaction associated with hypotension (SBP < 100 mm Hg).
5. Grade 3 or 4 Cytokine Release Syndrome (CRS) (see Appendix V).
6. A DLT may trigger the administration of rimiducid, which itself is defined as a DLT. For example, Grade 3 CRS not responding to standard clinical management with supportive measures, dexamethasone, and tocilizumab, or Grade 4 CRS will trigger the administration of rimiducid. The indication for rimiducid administration will be discussed with the medical monitor.

An evaluable patient will be one that has received the PBT infusion and has completed safety evaluations up to 6 weeks later and thus enables assessment of DLT.

7.5 Patient Withdrawal Criteria

Patients will be removed from study therapy if any of the following occur:

1. Unacceptable toxicity;
2. Any complication, related or not, to GD2-iCAR-PBT infusion requiring intensive care, ventilation or blood pressure support;
3. Clinical melanoma progression that warrants cessation of dabrafenib and trametinib;
4. Urgent need for alternative therapy (including but not limited to disease progression) incompatible with continuation of treatment;
5. Any medical condition that in the opinion of the investigator precludes the safe continuation of the study;

Gargett T, *et al. J Immunother Cancer* 2024; 12:e008659. doi: 10.1136/jitc-2023-008659

6. Patient request;
7. Failure to adhere to the protocol requirements.

Patients who withdraw from the study are not able to receive any further infusions of GD2-iCAR-PBT. Thereafter, they will be monitored for safety and scored for toxicity. Patients will be followed until resolution of the adverse event or until there has been stabilisation if resolution has not occurred.

Patients who withdraw from the study or fail to adhere to the protocol requirements (thereby rendering them un-evaluable for DLT assessment) at any time after the PBT infusion, will be replaced by another patient. This replacement patient will be treated at the same dose level as the withdrawn patient, as long as the patient who withdrew from the study or failed to adhere to the protocol requirements did not correspond to an adverse reaction.

Patients who receive an infusion of GD2-iCAR-PBT will be monitored for safety for the duration of the study and will be included in all safety and efficacy analyses (intent-to-treat principle).

8. TREATMENT OF PATIENTS

8.1 Patient Enrolment, Registration, and Assignment to a Treatment

Patients will be assigned in sequential order.

8.2 Dose Levels

The following dose levels will be evaluated:

Dose Level 2: 2 x 10^7 cells/m^2
Dose Level 2: 1 x 10^8 cells/m^2

8.3 Dosing Schedules

Three different dosing schedules will be evaluated as based on previous gene-modified T cell immunotherapy studies for lymphoma and neuroblastoma (143). A modified continual reassessment method will be employed with cohorts of size 2 per dose level (see **section 6.0**). Each patient will receive 1 injection of one dose of GD2-iCAR-PBT.

8.4 Site of Administration

The first infusion of GD2-iCAR-PBT will be given in the Pain Anaesthesia Research Clinic (PARC) overnight stay unit at RAH.

8.5 Premedication

Patients will be premedicated with promethazine 25 mg IV and paracetamol 1,000mg. Steroids should be avoided given their detrimental effect on the survival of the infused T cells.

8.6 Cell Administration

GD2-iCAR-PBT will be given by intravenous injection over 5-10 minutes into a peripheral vein and the IV flushed with saline. The infusion will be delivered by a volumetrically controlled delivery system.

- Between 2 and 30mL of cells will be infused

- The volume of infusion will depend upon the concentration of the cells when frozen, the dose level, and the size of the patient

8.7 Monitoring

Patients will remain as inpatients until 24 hours after the PBT infusion. Pulse rate and oximetry will be measured continuously while the patient is resident. Vital signs (resting blood pressure, temperature, respirations) will be monitored every 15 minutes after the injection for one hour, then every 30 minutes up to 4 hours post administration, then hourly until 8 hours after the administration and then every 4 hours until 24 hours post dose (except when sleeping).

If an allergic reaction occurs, drug administration will be stopped and appropriate treatments will be administered including oxygen and adrenaline, promethazine and steroids (see Appendix III for management of hypersensitivity reactions).

If the PBT infusion is to be repeated (see **Section 6.1**) and the patient has previously tolerated the infusion without any indication of hypersensitivity reaction, then inpatient observations will again be conducted over 24 hours.

8.7.1 Preparation of GD2-iCAR-PBT for Injection

Each dose of GD2-iCAR-PBT will be prepared from frozen vials by the SA Pathology Therapeutic Products Facility no longer than four hours before it is scheduled to be administered to the patient. Preparation will be conducted using a class II biosafety cabinet according to the Standard Operating Procedure. The final product will be supplied by the SA Pathology Therapeutic Products Facility to the investigator in a 30mL syringe and will be stored at 2-8°C in such case where it cannot be administered immediately to the patient. Total storage time should not exceed four hours from the time when preparation is completed (this will be clearly displayed on the product label).

The GD2-iCAR-PBT should be administered as an intravenous infusion over 5-10 mins using a DEHP-Free PVC, non-vented blood product infusion set with a 200 micron in-line filter, followed by a 30 - 100 mL normal saline flush.

8.8 Administration of Rimiducid (AP1903) Homodimeriser Drug

Patients who develop dose-limiting toxicity, which is assessed to be related to the infusion of GD2-iCAR T cells, will receive 0.4mg/kg of rimiducid as a 2 hour infusion – based on published PK data which show plasma concentrations of 10-1275ng/mL over the 0.01mg/kg to 1.0mg/kg dose range with plasma levels falling to 18% and 7% of maximum at 0.5 and 2hrs post dose (121).

8.8.1 Instructions for Preparation

Rimiducid for Injection is a concentrated solution of 2.33mL in a 3mL vial, at a concentration of 5mg/mL, (i.e. 10.66mg per vial). Before administration, the calculated dose will be diluted to 100mL in 0.9% normal saline for infusion.

8.8.2 Instructions for Infusion

The premedication for rimiducid administration is promethazine 25 mg IV and paracetamol 1,000mg PO 30 minutes before the rimiducid dose.

Rimiducid for Injection (0.4mg/kg) in a volume of 100mL will be administered *via* IV infusion over 2 hours, using a non-DEHP, non-ethylene oxide sterilized infusion set and an infusion pump.

Gargett T, *et al. J Immunother Cancer* 2024; 12:e008659. doi: 10.1136/jitc-2023-008659

8.9 Blinding/Unblinding

Not applicable. This is an open label study.

8.10 Concurrent Anti-cancer Chemotherapy

Patients with progressive disease may receive other therapy if needed at the discretion of their attending physician.

9. STUDY ASSESSMENTS

9.1 Safety Assessments

Safety assessments will be based on medical review of adverse event reports and the results of vital sign measurements, electrocardiograms and traces, physical examinations including regular dermatological assessments, and clinical laboratory tests. The incidence of adverse events will be tabulated and reviewed for potential significance and clinical importance. Adverse Events will be graded according to the NCI Common Terminology Criteria for Adverse Events (CTCAE v.4.0).

9.2 Efficacy Assessments

Tumour response will be determined for all patients with measurable lesions using the new international criteria proposed by the revised Response Evaluation Criteria in Solid Tumours (RECIST) guideline (version 1.1) (144). Changes in the largest diameter (unidimensional measurement) of the tumour lesions and the shortest diameter in the case of malignant lymph nodes are used in the RECIST 1.1 criteria.

The first assessment will be made at 6 weeks after the GD2-iCAR-PBT infusion. Scanning may also need to be performed at other times as per clinical need. In addition, a confirmatory scan should be obtained 4 weeks following initial documentation of objective response.

9.2.1 Malignant Disease Evaluation

To assess objective response, it is necessary to estimate the overall tumour burden at baseline to which subsequent measurements will be compared. Only those patients who have measurable disease present at baseline (defined by the presence of at least one measurable lesion), have received at least one cycle of therapy, and have had their disease re-evaluated will be considered evaluable for objective response. These patients will have their response classified according to the definitions stated below.

All measurements should be taken and recorded in metric notation using a ruler or calipers. All baseline evaluations should be performed as closely as possible to the beginning of treatment and will be performed within two weeks of treatment by a qualified member of the study team.

The same method of assessment and the same technique should be used to characterise each identified and reported lesion at baseline and during follow-up. Imaging-based evaluation is preferred to evaluation by clinical examination unless the lesion(s) being followed cannot be imaged but are assessable by clinical exam.

Clinical lesions will only be considered measurable when they are superficial (e.g., skin nodules and palpable lymph nodes) and ≥ 10 mm diameter as assessed using calipers (e.g., skin nodules). In the case of skin lesions, documentation by color photography, including a ruler to estimate the size of the lesion, is recommended.

Gargett T, *et al. J Immunother Cancer* 2024; 12:e008659. doi: 10.1136/jitc-2023-008659

The term evaluable in reference to measurability will not be used because it does not provide additional meaning or accuracy.

At baseline, tumour lesions will be characterised as either measurable or non-measurable.

9.2.1.1 Measurable

Measurable lesions are defined as those that can be accurately measured in at least one dimension (longest diameter to be recorded) as $\geq$ 20 mm by chest x-ray, as $\geq$ 10 mm with CT scan, or $\geq$ 10 mm with calipers by clinical exam. All tumour measurements must be recorded in millimetres (or decimal fractions of centimetres).

To be considered pathologically enlarged and measurable, a lymph node must be $\geq$ 15 mm in short axis when assessed by CT scan (CT scan slice thickness recommended to be no greater than 5 mm). At baseline and in follow-up, only the short axis will be measured and followed.

If the measurable disease is restricted to a solitary lesion, its neoplastic nature should be confirmed by cytology/histology.

9.2.1.2 Non-Measurable

All other lesions (or sites of disease), including small lesions (longest diameter < 10 mm or pathological lymph nodes with ≥ 10 to < 15 mm short axis), are considered non-measurable disease.

Bone lesions, leptomeningeal disease, ascites, pleural/pericardial effusions, lymphangitis cutis/pulmonitis, inflammatory breast disease, and abdominal masses (not followed by CT or MRI), are considered as non-measurable.

Cystic lesions that meet the criteria for radiographically defined simple cysts should not be considered as malignant lesions (neither measurable nor non-measurable) since they are, by definition, simple cysts. 'Cystic lesions' thought to represent cystic metastases can be considered as measurable lesions, if they meet the definition of measurability described above. However, if non-cystic lesions are present in the same patient, these are preferred for selection as target lesions.

Masses growing in a previously radiated field (e.g. prostatic fossa mass) are measurable if documented to have increased in size since radiotherapy.

9.2.2 Definitions of Response

9.2.2.1 Target Lesions

All measurable lesions up to a maximum of 2 lesions per organ and 5 lesions in total, representative of all involved organs, should be identified as **target lesions** and recorded and measured at baseline. Target lesions should be selected on the basis of their size (lesions with the longest diameter), be representative of all involved organs, but in addition should be those that lend themselves to reproducible repeated measurements. It may be the case that, on occasion, the largest lesion does not lend itself to reproducible measurement in which circumstance the next largest lesion which can be measured reproducibly should be selected. A sum of the diameters (longest for non-nodal lesions, short axis for nodal lesions) for all target lesions will be calculated and reported as the baseline sum diameters. If lymph nodes are to be included in the sum, then only the short axis is added into the sum. The baseline sum diameters will be used as reference to further characterize any objective tumour regression in the measurable dimension of the disease.

Gargett T, *et al. J Immunother Cancer* 2024; 12:e008659. doi: 10.1136/jitc-2023-008659

9.2.2.1.1 Complete Response (CR)

Disappearance of all target lesions. Any pathological lymph nodes (whether target or non-target) must have reduction in short axis to < 10 mm. To be assigned a status of complete response, changes in tumour measurements must be confirmed by repeat assessments performed **no less than four weeks** after the criteria for response are first met.

9.2.2.1.2 Partial Response (PR)

At least a 30% decrease in the sum of the diameters of target lesions, taking as reference the baseline sum diameters. To be assigned a status of partial response, changes in tumour measurements must be confirmed by repeat assessments performed **no less than four weeks** after the criteria for response are first met.

9.2.2.1.3 Progressive Disease (PD)

At least a 20% increase in the sum of the diameters of target lesions, taking as reference the smallest sum on study (this includes the baseline sum if that is the smallest on study). In addition to the relative increase of 20%, the sum must also demonstrate an absolute increase of at least 5 mm. The appearance of one or more new lesions is also considered progression.

9.2.2.1.4 Stable Disease (SD)

Neither sufficient shrinkage to qualify for PR nor sufficient increase to qualify for PD, taking as reference the smallest sum diameters while on study. To be assigned a status of stable disease for this protocol, measurements must have met the stable disease criteria at least once after study entry at a minimum interval of at least 6 weeks and confirmed at week 14.

9.2.2.2 Non-target Lesions

All other lesions or sites of disease. Measurements of these lesions are not required, but the presence or absence of each should be noted throughout follow-up.

9.2.2.2.1 Complete Response (CR)

The disappearance of all nontarget lesions and normalization of tumour marker levels, if applicable. To be assigned a status of complete response, changes in tumour measurements must be confirmed by repeat assessments performed **no less than four weeks** after the criteria for response are first met.

9.2.2.2.2 Incomplete Response/Stable Disease (SD)

The persistence of one or more nontarget lesion(s) and/**or** the maintenance of tumour marker levels above the normal limits. To be assigned a status of stable disease for this protocol, measurements must have met the stable disease criteria at least once after study entry at a minimum interval of at least 6 weeks and confirmed at week 14.

9.2.2.2.3 Progressive Disease (PD)

The appearance of one or more new lesion(s) and/or unequivocal progression of existing nontarget lesions.

Gargett T, *et al. J Immunother Cancer* 2024; 12:e008659. doi: 10.1136/jitc-2023-008659

9.2.3 Symptomatic Deterioration

Patients with a global deterioration of health status requiring discontinuation of treatment without objective evidence of disease progression at that time should be classified as having symptomatic deterioration.

9.3 Evaluation of Patient's Best Overall Response

The best overall response is the best response recorded from the start of the treatment until disease progression/recurrence, taking as reference for progressive disease the smallest measurements recorded since the treatment started. The table below provides overall responses for all possible combinations of tumour responses in target and non-target lesions, with or without new lesions.

To be assigned a status of complete or partial response, changes in tumour measurements must be confirmed by repeat assessments performed **no less than four weeks** after the criteria for response are first met. To be assigned a status of stable disease for this protocol, measurements must have met the stable disease criteria at least once after study entry at a minimum interval of at least 6 weeks and confirmed at week 14.

9.3.1 Overall Response for all Possible Combinations of Tumour Response

NOTE: For this protocol, time to radiographic progression is being evaluated as follows.

Target Lesions	Non-target Lesions	New Lesions	Overall Response
CR	CR	No	CR
CR	Incomplete response/SD	No	PR
PR	Non-PD	No	PR
SD	Non-PD	No	SD
PD	Any	Yes or No	PD
Any	PD	Yes or No	PD
Any	Any	Yes	PD

CR = complete response; PR = partial response; SD = stable disease; PD = progressive disease

9.3.2 First Documentation of Response

The time between initiation of therapy and first documentation of PR or CR.

9.3.3 Confirmation of Response

To be assigned a status of complete or partial response, changes in tumour measurements must be confirmed by repeat assessments performed **no less than four weeks** after the criteria for response are first met.

9.3.4 Duration of Response

The duration of overall response is measured from the time measurement criteria are met for CR or PR (whichever is first recorded) until the first date that recurrent or progressive disease is objectively documented (taking as reference for progressive disease the smallest measurements recorded since the treatment started).

9.3.4.1 Duration of Overall Complete Response

The duration of overall CR is measured from the time measurement criteria are first met for CR until the first date that progressive disease is objectively documented.

9.3.4.2 Duration of Stable Disease

Stable disease is measured from the start of the treatment until the criteria for progression are met, taking as reference the smallest measurements recorded since the treatment started, including the baseline measurements. To be assigned a status of stable disease for this protocol, measurements must have met the stable disease criteria at least once after study entry at a minimum interval or at least 6 weeks and confirmed at week 14.

9.3.5 Methods of Measurement

Imaging based evaluation is preferred to evaluation by clinical examination. The same imaging modality must be used throughout the study to measure disease.

In the case of loco-regionally recurrent disease, clinical measurement and clinical photography of the lesions with a metric tape measure to be included in the photograph can be used at each of the evaluation time points.

9.3.5.1 CT and MRI

This guideline has defined measurability of lesions on CT scan based on the assumption that CT slice thickness is 5 mm or less. If CT scans have slice thickness greater than 5 mm, the minimum size for a measurable lesion should be twice the slice thickness. MRI is also acceptable in certain situations (e.g. for body scans).

Use of MRI remains a complex issue. MRI has excellent contrast, spatial, and temporal resolution; however, there are many image acquisition variables involved in MRI, which greatly impact image quality, lesion conspicuity, and measurement. Furthermore, the availability of MRI is variable globally. As with CT, if an MRI is performed, the technical specifications of the scanning sequences used should be optimized for the evaluation of the type and site of disease. Furthermore, as with CT, the modality used at follow-up should be the same as was used at baseline and the lesions should be measured/assessed on the same pulse sequence. It is beyond the scope of the RECIST guidelines to prescribe specific MRI pulse sequence parameters for all scanners, body parts, and diseases. Ideally, the same type of scanner should be used and the image acquisition protocol should be followed as closely as possible to prior scans. Body scans should be performed with breath-hold scanning techniques, if possible.

9.3.5.2 FDG-PET/CT

At present, the low dose or attenuation correction CT portion of a combined PET-CT is not always of optimal diagnostic CT quality for use with RECIST measurements. However, if the site can document that the CT performed as part of a PET-CT is of identical diagnostic quality to a diagnostic CT (with IV and oral contrast), then the CT portion of the PET-CT can be used for RECIST

Gargett T, *et al. J Immunother Cancer* 2024; 12:e008659. doi: 10.1136/jitc-2023-008659

measurements and can be used interchangeably with conventional CT in accurately measuring cancer lesions over time. Note, however, that the PET portion of the CT introduces additional data which may bias an investigator if it is not routinely or serially performed.

9.3.5.3 Chest X-Ray

Lesions on chest x-ray are acceptable as measurable lesions when they are clearly defined and surrounded by aerated lung. However, CT is preferable.

9.3.5.4 Clinical Examination

Clinically detected lesions will only be considered measurable when they are superficial (e.g., skin nodules and palpable lymph nodes). For skin lesions, documentation by colour photography, including a ruler to estimate size of the lesion, is recommended. Photographs should be retained at the institution.

9.3.5.5 Cytology and Histology

Cytological and histological techniques can be used to differentiate between complete and partial response in rare cases (e.g., after treatment to differentiate residual benign lesions and residual malignant lesions in germ cell tumours). Cytological confirmation of the neoplastic nature of any effusion that appears or worsens during treatment is required when the measurable tumour has met response or stable disease criteria.

9.3.5.6 Endoscopy and Laparoscopy

The utilization of these techniques for objective tumour evaluation is not advised. However, such techniques may be useful to confirm complete pathological response when biopsies are obtained or to determine relapse in trials where recurrence following complete response (CR) or surgical resection is an endpoint.

9.3.5.7 Ultrasound

Ultrasound is not useful in assessment of lesion size and should not be used as a method of measurement. Ultrasound examinations cannot be reproduced in their entirety for independent review at a later date and, because they are operator dependent, it cannot be guaranteed that the same technique and measurements will be taken from one assessment to the next. If new lesions are identified by ultrasound in the course of the study, confirmation by CT or MRI is advised. If there is concern about radiation exposure at CT, MRI may be used instead of CT in selected instances.

9.4 Dermatology Assessments

Patients are required to have a full skin examination by a dermatologist to screen and monitor for squamous cell carcinoma (SCC), basal cell carcinoma (BCC), actinic keratosis, and keratoacanthoma (KA). Dermatology evaluation will be performed in the 28-day period before commencement of dabrafenib and trametinib, at 8 and 16 weeks on therapy, approximately every 12 weeks thereafter while the patient is receiving dabrafenib and trametinib. Patients should report to their doctor any new skin lesion or change, including rash and photosensitivity, while on study treatment and any suspicious lesions should be referred to a dermatologist for further evaluation as required.

The initial examination by the dermatologist should include a complete dermatological history of prior medications, and cutaneous SCC risk factors (i.e., radiation therapy, sun exposure, immunosuppression, previous SCC, use of tanning beds, precursor lesions and photochemotherapy

Gargett T, *et al. J Immunother Cancer* 2024; 12:e008659. doi: 10.1136/jitc-2023-008659

for psoriasis). Any lesion suspected of representing a new SCC, BCC, actinic keratosis, or keratoacanthoma identified by the dermatologist should be treated as per local standard of care. Skin biopsies of any suspicious lesions identified at baseline and during the study must be biopsied/excised and sent for pathological examination.

Patients who develop cutaneous SCC or any skin lesion during the trial may choose to continue or discontinue from the trial in consultation with the investigator. If the patient elects to continue in the trial, definitive treatment (i.e. surgical excision) of any SCC is required.

9.5 Laboratory Assessments

9.5.1 Routine Laboratory Investigations

The following routine laboratory investigations will be obtained as outlined in the Assessment Schedule (see **Section 10**):

- CBC and differential: pre-infusion on Day 0, on Days 7, 14, 28 and 42 post-infusion and then at 4, 8, and 12 months, and then yearly for a total of 15 years.
- Serum biochemistry (MBA_{20}) including electrolytes, creatinine, urea, bilirubin, albumin, AST, ALT: pre-infusion on Day 0, on Days 7, 14, 28 and 42 post-infusion and then at 4, 8, and 12 months, and then yearly for a total of 15 years.

9.5.2 Tests of GD2-iCAR-PBT persistence

Persistence of GD2-iCAR-PBT will be determined by quantitative real-time PCR for GD2-iCAR using the published method (99) and flow cytometry using the anti-idiotypic mAb, 1A7, to detect 14g2a epitopes in the GD2-iCAR. Blood samples will be collected pre-infusion (Day 0), post-infusion on Days 7, 14, 28 and 42 and then at 4, 8, and 12 months, and then yearly for a total of 15 years.

9.5.3 Tests for Human Ant-Mouse Antibodies (HAMA)

Serum will be prepared from blood drawn on Day 0 and on Day 42 (± 1) post-infusion and stored for measurement of HAMA in the event of a suspected immunologic reaction.

9.5.4 Safety Testing for Replication Competent Retrovirus (RCR)

Aliquots of cells and plasma will be collected pre-infusion, at 4, 8, 9, and 12 months, every 6 months for 4 years, and then yearly for a total of 15 years and archived for use in future studies for replication competent retrovirus (RCR) testing by PCR and the DERSE cell-based assay as required (145).

9.5.5 Immunological Tests

Immunological tests will include the assessment of cell mediated and humoral (antibody) responses. Tests will be conducted using blood samples taken first at the time that the patient consents to preparation of a T-cell product, which is before BRAF inhibitor therapy commences. Other blood samples are taken on the day of the PBT infusion (Day 0), and on Days 14, 28, and 42 (± 1 day) of the study. All tests will be analysed in batches later. Blood samples will be drawn at the study site and dispatched immediately for processing at the Immunopathology Laboratory (Women's and Children's Hospital [WCH], Adelaide, SA) for immunology testing as described in the WCH Manual. PBMC and plasma samples will be frozen and stored for later detection of cell-mediated and antibody responses to melanoma cells, respectively, as described in the WCH Manual. PBMC will also be used to extract RNA for RNA expression analysis studies to be performed at the Experimental

Therapeutics Laboratory of the Hanson Institute (RAH) and the SA Pathology ACRF Cancer Genomics Facility.

Blood samples for the preparation of serum will be processed on site and stored frozen on site until all time points for that patient have been collected. These samples will then be dispatched to the Immunopathology Laboratory (WCH), and will be used to test for the presence of both human anti-mouse antibodies (HAMA) by ELISA.

Testing for cell-mediated immune responses will be conducted at the Immunopathology Laboratory (WCH) as described below:

- For cell-mediated immune responses, the production of the cytokines IFNγ, TNF, IL-2, IL-4, IL-17, IL-10 and LT by cultured PBMC. The effect of melanoma-antigen specific stimulation on cellular proliferation measured by tritiated thymidine incorporation assay and on cytokine production will be assessed. Cytokine production will be measured using the Cytometric Bead Array assay in accordance with the protocol described in the WCH Manual.
- The effect of melanoma-antigen-specific stimulation on IFNγ production by cultured PBMC will be assessed by ELISPOT analysis in accordance with the protocol described in the WCH Manual.
- For patients determined to have HLA-A2.1^{+} PBMC, MHC tetramer analysis for HLA-A2.1-bound peptides derived from melanoma differentiation antigens will be conducted by flow cytometry.

Testing for humoral immune responses will be conducted at the Experimental Therapeutics Laboratory of the Hanson Institute (RAH) as described below:

- The binding of pre- and post-infusion plasma antibodies to MM200 cells, other melanoma cell lines for which different levels of GD2 expression [4405M (GD2^{-}), CLB (GD2^{lo}), SENMA (GD2^{int}), P1143 (GD2^{hi})] have been characterized (57), and the unrelated K562, MOLT-4 and Jurkat leukaemic cell lines will be studied using a method developed by the PI (146). If available, Ab reactivity will also be measured against the patient's autologous tumour cell line derived from short-term melanoma cultures of post-infusion biopsy samples using a method adapted from Ref. (147).

9.5.6 Tumour Biopsies

An optional tumour biopsy may be collected Days -10 to -1 and at any time between Days 4 to 14 post-infusion. If suitable, a short-term melanoma culture will be derived from the post-infusion biopsy sample using a method adapted from Ref. (147). An optional tumour biopsy may also be collected at the time of tumour progression in subjects who have had a response and remain on dabrafenib and trametinib. In each case, biopsies would be of readily accessible tumour lesions in sites such as skin and lymph nodes. Tumour biopsies will be collected by punch biopsy or by core needle. Samples will be fixed immediately in formalin before processing overnight, and if sufficient material is available, it will be embedded in OCT and frozen.

9.5.7 Electrocardiogram (ECG) Assessment

QTC and QRS intervals will be determined from a single ECG taken at baseline (prior to treatment), and at 2, 4, and 24 hours post-infusion.

9.5.8 Multigated acquisition (MUGA) Scan Assessment

MUGA scans will only be performed in patients receiving dabrafenib and trametinib. Scans will be done at baseline, day 14 (± 1) post-infusion (approximately one month after initiation of dabrafenib and trametinib) and at 4, 8, and 12 months post-infusion. Less than 50% of patients continue to receive dabrafenib and trametinib beyond this time but those who do will continue to have MUGA scans approximately every 4 months as part of routine clinical care.

CONFIDENTIAL

10. ASSESSMENT SCHEDULE

Study	Screening	Treatment				Follow-up	
	Days - 28 to -1	Day 0[‡]	Day 7 (± 1)	Days 14 and 28 (± 1)	Day 42 (± 1)	Months 4, 8, and 12	Per year x 14 years
Informed Consent	X						
T cell infusion		X					
Weight	X	X	X	X	X	X	X
Vital Signs	X	X^	X	X	X	X	X
History/PE	X	X	X	X	X	X	X
ECOG	X	X	X	X	X	X	X
Dermatology Evaluation	X[§]						
CBC	X	X	X	X	X	X	X
MBA$_{20}$	X	X[#]	X	X	X	X	X
HIV, Hepatitis B & C	X						
Urinalysis	X	X	X	X	X	X	X
Pregnancy Test*	X						
ECG	X	X***					
MUGA scan	X[†]			X[†]		X[†]	
Tumour biopsy	X	X[a]			X[b]		
Dendritic cell banking	X						
Immunological Assays**		X		X	X		
Stored serum for HAMA		X[c]			X	X	X
Stored PBMC for anti-iCasp9 studies		X[d]			X	X	X
Stored serum for CRP, cytokines	X	X	X	X	X		
Stored plasma for anti-tumour Ab and GD2		X[e]		X	X		

Gargett T, et al. J Immunother Cancer 2024; 12:e008659. doi: 10.1136/jitc-2023-008659

CONFIDENTIAL

**quantification studies**							
**Persistence studies**		**X[f]**	**X**	**X**	**X**	**X**	**X**
**Disease Evaluation per RECIST**	**X**				**X**		
**RCR testing**	**X[g]**					**X**	**X**
**AE Monitoring**	**X**	**X**	**X**	**X**	**X**	**X**	**X**
**Review concomitant medications**	**X**	**X**	**X**	**X**	**X**		

§ Dermatology evaluations will be performed by a dermatologist in the 28-day period before commencement of dabrafenib and <u>trametinib</u>, at 8 and 16 weeks after commencement of dabrafenib and <u>trametinib</u>, and then approximately every 12 weeks thereafter while the patient receives dabrafenib and <u>trametinib</u>.

^ **Vital signs will be collected at screening, pre-dose, and will then be monitored every 15 minutes post-administration for 1 hr, then every 30 minutes up to 4 hr post-administration, then hourly until 8 hr post-administration and then every 4 hr until 24 hr post-administration (except when sleeping).**

‡ Day 0 represents the day GD2-iCAR-PBT are infused

* For women of childbearing potential, serum pregnancy testing will be required at screening

** Blood for immunological assays are collected in green top (Lithium heparin) tubes. Immune function studies include: (i) MHC tetramer analysis, (ii) Tritiated thymidine incorporation assay, (iii) Cytometric Bead Array for measure of cytokine production by cultured PBMC, (iv) ELISPOT analysis (cultured PBMC)

*** baseline (pre dose), 2, 4, and 24 hours after T cell infusion

# pre-dose and at 24 hours post dose

† MUGA scans will only be performed in patients receiving dabrafenib and trametinib. Scans will be done at baseline, day 14 (± 1) post-infusion (approximately one month after initiation of dabrafenib and trametinib) and at 4, 8, and 12 months post-infusion. Less than 50% of patients continue to receive dabrafenib and trametinib beyond this time but those who do will continue to have MUGA scans approximately every 4 months as part of routine clinical care.

a An optional tumour biopsy may be collected between Days -10 to -1 and at any time between Days 4 to 14 post-infusion

b An optional tumour biopsy may be collected at the time of tumour progression in subjects who have had a response and who remain on dabrafenib and trametinib

c Stored serum samples: serum will be prepared from 3mL of blood (white top) at pre-infusion (Day 0), at the end of the treatment evaluation period (Day 42 ± 1), and at 4, 8, 12 months, and yearly for a total of 15 years. Samples will be stored at -20°C. Serum samples will be used at a later time by the investigators for the detection of human anti-mouse antibodies (HAMA) if needed

d Stored PBMC samples: PBMC will be prepared from 3 x 9mL of blood (Li heparin tubes) at pre-infusion (Day 0), at the end of the treatment evaluation period (Day 42 ± 1), and at 4, 8, 12 months, and yearly for a total of 15 years. Samples will be stored at -80°C. PBMC samples will be used at a later time by the investigators for the detection of anti-iCasp9 cellular immune responses if needed

e Stored plasma samples: plasma will be prepared from Li heparin tubes and later used in batched FACS analyses of anti-tumour antibodies and GD2 quantification studies

Gargett T, *et al. J Immunother Cancer* 2024; 12:e008659. doi: 10.1136/jitc-2023-008659

[f] PCR for persistence studies of the CAR transgene are collected in purple top (EDTA) tubes (or the pellet from a white top tube); PBMC will be prepared and stored for later flow cytometry analysis using 1A7 mAb; samples will be collected at baseline and 6 hours post-infusion of GD2-iCAR-PBT

[g] Aliquots of cells will be prepared from Li heparin tubes pre-infusion, at 4, 8, 12 months, and yearly for a total of 15 years and archived for future studies for replication competent retrovirus (RCR) testing by PCR and DERSE cell-based assay as required

Gargett T, *et al. J Immunother Cancer* 2024; 12:e008659. doi: 10.1136/jitc-2023-008659

10.1 Screening Procedures and Assessments

See schedule of activities above for details.

10.2 Post Study Follow-up Procedures and Assessments

At clinic visits, a routine history will be taken and a physical examination performed. Blood will be obtained for routine CBC, biochemistry (MBA_{20}) and immunological testing; a urinalysis will also be performed (See schedule of activities for details). If a patient is unable to attend the clinic in the follow-up period then investigator, research nurse or clinical trials coordinator will then contact the patient by phone or email.

Any serious adverse events occurring within these time periods should be recorded in the patient's case report form (CRF) and the appropriate serious adverse event recording tool. Any patient who is discontinued from therapy should also be followed according to the above schedule for safety considerations.

11. ADVERSE EVENTS

11.1 Safety Parameters

Safety parameters will include AEs, vital signs, clinical laboratory tests, physical examinations, ECGs and multigated acquisition (MUGA) scans.

11.2 Adverse Events

An AE is any untoward medical occurrence in a patient or clinical investigation patient administered a pharmaceutical product and which does not necessarily have a causal relationship with the product. An AE can therefore be any unfavourable and unintended sign (including a new, clinically important abnormal laboratory finding), symptom, or disease temporally associated with the product, whether or not related to the product.

Pre-existing diseases or conditions will not be considered AEs unless there is an increase in the frequency or intensity, or a change in the quality of disease or condition.

An AE does not include:

- medical or surgical procedures (e.g. surgery, endoscopy, tooth extraction, transfusion); the condition that leads to the procedure is an AE
- pre-existing diseases or conditions present or detected prior to start of study product administration that do not worsen
- situations where an untoward medical occurrence has not occurred (e.g. hospitalisation for elective surgery, social and/or convenience admissions)
- overdose of either study product or concomitant medication without any signs or symptoms unless the patient is hospitalised for observation

11.3 Adverse Event Reporting

At each required study visit, all adverse events (AE) that have occurred since the previous visit must be recorded in the adverse event record of the patient's case report form (CRF). The

information recorded should be based on the signs or symptoms detected during the physical examination and/or clinical evaluation of the patient. AEs should be monitored or evaluated using non-leading questions.

The following AE information must be included (when applicable): the specific condition or event (AE name or term) and direction of change; whether the condition was preexisting (i.e., an acute condition present at the start of the study or history of a chronic condition) and, if so, whether it has worsened (e.g., in severity and/or frequency); the dates and times of occurrence (start dates and stop dates); or an indication of 'ongoing'; intensity; seriousness/severity; investigator's opinion regarding the causal relationship to study drug; action taken; and outcome.

The causal relation between an AE and the study drug will be determined by the investigator based on his or her clinical judgment.

When assessing the relationship between administration of a study drug and an AE, the following should be considered:

Temporal relationship between administration of the study drug and the AE

Biological plausibility of relationship

Patient's underlying clinical state or concomitant agents and/or therapies

The categories for classifying the Investigator's opinion regarding the relationship of an AE to investigational product(s) are listed below.

Definite:	This causal relationship is assigned when the AE:<br>i. Follows a reasonable temporal sequence from the administration of the investigational product.<br>ii. Abates upon discontinuation of the investigational product<br>iii. Is confirmed by the re-appearance of the AE on repeat exposure
Probable:	This causal relationship is assigned when the AE:<br>i. Follows a reasonable temporal sequence from the administration of the investigational product<br>ii. Abates upon discontinuation of the investigational product; and<br>iii. Cannot be reasonably explained by known characteristics of the patient's clinical state.
Possible	This causal relationship is assigned when the AE:<br>i. Follows reasonable temporal sequence from the administration of the investigational product, but

	ii. Could have been produced by the patient's clinical status or other modes of therapy administered to the patient.
Unlikely:	The temporal association is such that the investigational product is not likely to have had an association with the AE.
Not related:	The AE is definitely not associated with the investigational product administered.

CTCAE version 4.0 will be used to grade adverse events. The following definitions should be used when determining the severity of an AE, especially if the AE is not clearly defined in the CTCAE. In some cases, removal of study drug may be appropriate.

Mild (grade 1): The AE is noticeable to the patient but does not interfere with routine activity. The AE does not require discontinuing administration or reducing the dose of the study drug.

Moderate (grade 2): The AE interferes with routine activity but responds to symptomatic therapy or rest. The AE may require reducing the dose but not discontinuing administration of the study drug.

Severe (grade 3): The AE significantly limits the patient's ability to perform routine activities despite symptomatic therapy. In addition, the AE leads to discontinuing administration or reducing the dose of the study drug.

Life Threatening (grade 4): The AE requires discontinuing administration of the study drug. The patient is at immediate risk of death.

Death (grade 5): The AE resulted in death.

11.3.1 Action(s) Taken

Action(s) taken may consist of:

None:	No actions taken.
Discontinued Study Drug:	Study drug was permanently discontinued because of the AE.
Treatment/Hospitalisation:	Specified medication (to be listed on the concomitant medication chart) has been used as a countermeasure and/or patient has required to be hospitalised or hospitalisation has been prolonged (SAE)
Dose modifications for non-investigational product:	Dose modifications for dabrafenib and trametinib will made according to the latest version of the Product Information for each drug

Others:	Other actions, such as an operative procedure were required because of the AE

11.3.2 Definition of Expectedness

An expected AE is one for which the nature or severity is consistent with the known AE profile of the product. For an investigational product, the known information is contained in the Investigator's Brochure. For a marketed product, the known information is contained in the current package insert for the product.

An unexpected AE is one for which the specificity or severity is not consistent with the current Investigator's Brochure (IB). For example, hepatic necrosis would be unexpected (greater severity) if the IB only listed elevated hepatic enzymes or hepatitis. Likewise, cerebral thromboembolism and cerebral vasculitis would be unexpected (greater specificity) if the IB only listed cerebral vascular accidents.

Furthermore reports which add significant information on specificity or severity of a known, already documented adverse reaction constitute unexpected events. Examples would be (a) acute renal failure as an expected adverse reaction with a subsequent new occurrence of interstitial nephritis and (b) hepatitis with a first occurrence of fulminant hepatitis.

11.3.3 Definition of Outcome at the Time of Last Observation

The outcome at the time of last observation will be classified as:

- Recovered,
- Recovered with Sequelae,
- Continuing,
- Death*,
- Other

*Only select as an outcome when the AE resulted in death. If more than one AE is possibly related to the patient's death, the outcome of death should be indicated for each such AE.

11.3.4 Follow-up of Patients with an Adverse Event

Any AE will be followed to a satisfactory resolution, until it becomes stable, or until it can be explained by another known cause(s) (i.e., concurrent condition or medication) and clinical judgment indicates that further evaluation is not warranted or until 28 days after the patients final study visit. All findings relevant to the final outcome of an AE must be reported in the patient's medical record.

11.4 Serious Adverse Events

A serious adverse event (SAE) is any untoward medical occurrence that at any dose:

- Results in death

- Is life-threatening
- Requires in-patient hospitalisation or prolongation of existing hospitalisation
- results in permanent (persistent) disability/incapacity
- is a congenital anomaly
- is an important medical event.

Medical and scientific judgment should be exercised in deciding whether it is appropriate to consider other situations serious, such as important medical events that may not be immediately life-threatening or result in death or hospitalisation but may jeopardize the patient or may require intervention to prevent one of the other outcomes listed in the definition above.

Examples of such events are intensive treatment in an emergency room or at home for allergic bronchospasm, blood dyscrasias or convulsions that do not result in hospitalisation, or development of drug dependency or drug abuse.

An elective hospital admission to treat a condition present before exposure to the test drug, or a hospital admission for a diagnostic evaluation of an AE, does not qualify the condition or event as an SAE.

A newly diagnosed pregnancy in a patient that has received a test product is not considered as an SAE unless it is suspected that the investigational product(s) interacted with a patient's postmenopausal status and led to the pregnancy. A congenital anomaly in an infant born to a mother who was exposed to the test product during pregnancy is an SAE.

11.4.1 Serious Adverse Event Reporting

Serious adverse events require expeditious handling and MUST be reported within 24 hours by FAX to the Sponsor's Safety Monitor. The relevant contact details are provided below:

RAH Cancer Centre Cancer Clinical Trials Unit
Fax : + 61 8 7074 6211
Tel : + 61 8 7074 2342
Mobile : + 61 (0) 0466 014 272
Email: anne.milton@sa.gov.au

Any serious adverse event (SAE), regardless of causal relationship, must be reported to the Sponsor's Safety Monitor immediately (no later than 24 hours after the investigator becomes aware of the SAE) by faxing a completed SAE form to the number listed above. Compliance with this time requirement is essential so that the Sponsor may comply with its regulatory obligations.

Follow-up information relating to an SAE must be reported to the Sponsor's Safety Monitor within 24 hours of receipt by the Investigator by faxing a completed SAE form to the number listed above. The patient should be observed and monitored carefully until the condition resolves or stabilizes or its cause is identified.

Any emergency must be reported to the Sponsor Medical Monitor immediately (within 24 hours) by contacting +61 405 670 420.

All SAE's will be reported to the CALHN Human Research Ethics Committee within 72 hours.

12. STATISTICAL METHODS

12.1 Sample Size Determination

This is an exploratory Phase 1 study and the sample size is appropriate for a study of this type.

12.2 Definition of Analysis Populations

The primary analysis population is defined according to the intent-to-treat (ITT) principle. The ITT population comprises all patients who enrol in the Protocol and is based on the cohort and dose level the patient was assigned to, regardless of the actual cohort or dose level. The ITT population will be used for all summaries of baseline and demographic data as well as all summaries of efficacy data. In addition, all listings will be produced for the ITT population.

The Safety population comprises all enrolled patients who receive at least one dose (full or partial) of GD2-iCAR-PBT and is based on the actual cohort and dose level, if this differs from that to which the patient was assigned to. This population will be used for the summaries of all safety data.

No group (except patients with cognitive impairment) will be excluded from participation. Patients of both genders, from all racial and ethnic groups are eligible for this trial. There is no information suggesting differences in the distribution, tissue uptake or disease response of gene-modified PBT among racial or ethnic groups or between the genders. Efforts will be made to extend the accrual to a representative population, but, in a trial accruing small numbers of patients, the diversity may not be optimal.

For patients who are withdrawn from the study prior to study completion, all data compiled up to the point of discontinuation will be used for analysis. All withdrawals will be included in all analyses up to the time of withdrawal. Patients who are withdrawn prematurely from study treatment will be included in all analyses regardless of the duration of treatment.

12.3 Statistical Analyses

This is a phase I study. Descriptive statistics will be used.

Quantitative variables will be described in terms of median, minimum and maximum for each cohort (dose level) and in terms of mean, median, quartiles, minimum and maximum for the overall population. Qualitative variables will be presented in terms of frequency for each cohort and overall.

The primary objectives of this study will be to determine:

(i) The feasibility of preparing T cell products for administration. This will entail determining the frequency of successfully administered products. Acceptable feasibility is defined as the administration to patients of ≥ 80% of prepared T cell products.

(ii) The safety profile and dose limiting toxicities of a single intravenous injection of autologous GD2-iCAR-PBT (the T cell product). This will entail determining the frequency of DLT. The MTD is the highest dose level administered on this trial with acceptable toxicity, and is defined as the dose causing a DLT in 20% of patients.

To determine the safety of one intravenous injection of autologous PBT directed to GD2 through their CAR in patients with metastatic GD2-positive malignancy, we will implement a Bayesian dose-finding phase-I trial design, called the modified continual reassessment method (mCRM) (140) (141). Patients will receive one injection of GD2-iCAR-PBT according to the following dosing schedule:

Dose Level 1: 1 x10^7 cells/m^2

Dose Level 2: 2 x10^7 cells/m^2

Dose Level 3: 1 x 10^8 cells/m^2

Each patient will be followed for 6 weeks after the PBT infusion for evaluation of dose limiting toxicity (DLT). The toxicity will be evaluated by NCI Common Terminology Criteria for Adverse Events version 4.0. Any toxicity that is irreversible or life threatening or Grade 3 or 4 considered to be primarily related to the PBT injection will be classified as dose limiting toxicity (DLT). All patients within a dose cohort should have completed the 6-week window after the PBT infusion for assessment of dose limiting toxicity as defined below prior to enrolment of patients into the next recommended dose level.

The mCRM will be implemented with a cohort of size 2 based on the exponential dose-toxicity model. The fixed prior probabilities of DLT for the three dose levels indicated above were assumed to be 5%, 8% and 25%, respectively. To ensure patient safety, the mCRM starts from the lowest dose level and limits the dose escalation to one dose level at a time. DLT event in the lower dose cohort will be used to update the dose-toxicity curve. The next patient cohort is assigned to the dose level with an associated probability of DLT closest to the target probability of 20%. This process continues until at least 10 patients have been accrued into the trial or 6 patients have been treated at the current MTD. Depending on patient availability and dose escalation, a maximum of 12 patients will be recruited into this Phase I trial. The final MTD will be the dose with probability closest to the target toxicity rate at these termination points. Since GD2-iCAR-PBT generation for this trial takes 3-4 weeks we plan to generate PBT products for up to 25 patients accounting for potential ‘drop outs’ and change in patients’ eligibility status (80% completion rate).

Statisticians working with our U.S. colleagues at Cell and Gene Therapy Center, Baylor College of Medicine, Houston, Texas, performed simulations with 10,000 replications in order to compare the operating characteristics of mCRM with a standard 3+3 dose-escalation design. Compared to the 3+3 design, the proposed mCRM design allows a better estimate of the MTD based on a higher probability of declaring the third dose level as the MTD, allocated a smaller number of patients at lower and likely ineffective dose levels, afforded a slightly lower average total number of patients needed to complete the trial (11 versus 12), and yielded similar toxicities on average compared to the standard 3+3 design (1.5 for both designs). We expect a shallow dose-toxicity curve and feel comfortable with slightly more accelerated dose-escalations without a substantial compromise in patient safety.

During the study, patient toxicity outcomes will be monitored in real time in order to estimate the dose-toxicity curve and determine the dose level for the next patient cohort using one of the pre-specified dose levels**.** Patients in a cohort can be enrolled concurrently but both patients within a dose cohort should have completed the 6-week window after the PBT infusion for assessment of DLT prior to enrolment of patients into the next recommended dose level. To

increase safety, patients enrolled in the same dose cohort will not be treated within the same week. The recommended phase II single-agent dose will be based on the findings of the toxicity, immunoreactivity, and activity profile of the patients in this phase I study. Safety and toxicity outcomes will be summarised by dose levels.

To determine feasibility of the protocol, the following calculation will be done:

$$\% \quad \frac{\text{frequency of T cell products administered}}{\text{frequency of T cell products prepared}}$$

and acceptable feasibility will be defined as ≥ 80%.

12.3.1 Baseline Characteristics

These will be listed.

12.3.2 Treatment Compliance

Patients will be treated at the investigational centre to document and ensure treatment compliance.

12.3.3 Safety Analyses

Incidence of adverse events and of marked abnormalities of clinical laboratory tests will be summarised by dose/cohort.

The corresponding number of AE's from baseline will be measured. The results will be tabulated by dose/cohort and study day.

12.3.4 Gene Transfer and Immunology Data

These will be summarised using descriptive statistics.

During the course of the study, the persistence and immune effects of GD2-iCAR-PBT lines will be studied. Immunological parameters including cellular response frequencies and cytokine levels will be summarised using descriptive statistics at each time point of follow-up. Positivity for 1A7 by flow cytometry and quantitative real-time PCR for GD2 CAR over the follow-up period will also be summarised using descriptive statistics. Growth curves representing measurements over time within a patient will be generated to visualise general patterns of immune response. Pair-wise comparisons of changes in these endpoints over time compared to pre-infusion will be implemented using paired *t*-tests or Wilcoxon signed-ranks tests. Repeatedly measured immunological parameters will be analysed using random coefficient regression models. This will allow us to model patterns of immune response per patient while allowing for varying intercepts and slopes for a patient.

We will also include dose level as an independent variable in the model to account for the different dose levels received by the patients. A similar strategy will be employed to analyse the kinetics of T cell survival. We will assess the validity of the normality assumption for the analysis of these parameters and perform transformations (e.g. log, square root), if necessary, to achieve normality. These modelling strategies will be considered exploratory based on the limited patient numbers in this initial trial.

Gargett T, *et al. J Immunother Cancer* 2024; 12:e008659. doi: 10.1136/jitc-2023-008659

Pre- and post-treatment biopsies will be analysed by immunohistochemistry for GD2 expression and T cell infiltrates will be identified by staining for CD3, CD4, or CD8. Tumour biopsies that have been collected at progression will also be stained for T cell infiltrates and GD2 expression. If T cell infiltrates are found then staining with 1A7 (for GD2 CAR) will be developed in FFPE or frozen material. In addition, these biopsies may be tested for cancer-testis antigens such as MAGE-A3 or melanocyte differentiation antigens such as melan-A/MART-1, gp100, and tyrosinase (HMB45). The results will be compared with the same stains done on any available tumour biopsy material to determine if antigen loss has occurred in response to immune selection pressure. Pre- and post-treatment biopsy findings will be summarised.

12.3.5 Efficacy Analyses

Individual tumour responses (as defined by the RECIST 1.1 criteria) will be listed by dose/cohort.

Frequencies and proportions of responders will be summarised overall and by dose groups if there are enough patients per dose levels. The results of this study will not be definitive but only suggestive. A phase II trial will be undertaken to study the efficacy of the treatment after determining the safe MTD.

Any patient with progressive disease who receives other therapy will come off study with respect for evaluation of short-term toxicity but will remain on long-term gene transfer follow up as outlined in the Assessment Schedule (see **Section 10**).

12.3.6 Data Safety Monitoring

The Principal Investigator and Sponsor will be responsible for overseeing Safety Monitoring. At each cohort, the data safety monitoring board (DSMB) comprising the Sponsor Medical Monitor, Sponsor Safety Monitor, Statistician, a qualified physician independent of the Study, and the Principal Investigator will convene, either *via* teleconference and/or in person, to discuss the results before proceeding to the next patient or cohort, respectively. The safety data will be primarily examined, efficacy will also be assessed and the decision to escalate or expand cohorts will be made by the board. This information will be provided in writing to the RAH HREC prior to any subsequent dosing. Laboratory and clinical data will be reviewed on each patient and any new significant observation(s) found during the course of the study, which may affect a patient's willingness to continue participation in the study will be explained. Copies of correspondence will be kept in the study binder.

Confidentiality of information concerning patient will be maintained including in all publications and presentations resulting from this study. Names of patients or material identifying patients will not be released without permission, except as such release is required by law. Records in each institution are maintained according to current legal requirements, and are made available for review, for the purposes of monitoring and source data verification.

Gargett T, *et al. J Immunother Cancer* 2024; 12:e008659. doi: 10.1136/jitc-2023-008659

13. ETHICAL CONSIDERATIONS AND ADMINISTRATIVE SECTION

13.1 Local regulations / Declaration of Helsinki

The Investigator will ensure that this study is conducted in full conformance with the protocol, the latest version of the "Declaration of Helsinki (and its amendments) and with the requirements of national drug and data protection laws of the countries in which the research is conducted.

The Sponsor and the Investigators will ensure strict adherence to the provisions of the "Good Clinical Practice Guidelines" and national regulations.

13.2 Compliance With Protocol and Protocol Revisions

The study must be conducted as described in this approved protocol. The Investigator should not implement any deviation or change to the protocol without prior review and documented approval/favorable opinion from the RAH Human Research Ethics Committee, except where necessary to eliminate an immediate hazard(s) to study patients.

13.3 Informed Consent

The Informed Consent Form (ICF) will be reviewed with the prospective study patients or their legal representatives, and the Investigator will be available to answer questions regarding procedures, risks, and alternatives. The Principal Investigator or his/her entitled designee (as defined on the Delegation List) will obtain written informed consent from each patient or from the patient's legal representative or designee as defined by local law. Consent will be obtained before any protocol-specific procedures are performed.

Investigators must ensure that patients are clearly and fully informed about the purpose, potential risks, and other critical issues regarding clinical trials in which they volunteer to participate.

This study will have two ICFs: (1) Pre-Screening (2) Main Study. When required, verbal consent may be taken from patients for the Pre-Screening ICF as detailed below:

(1) The Pre-Screening ICF will allow for the testing of the presence of GD2 in patients tumour tissues. Patient's consent can either be verbal or written. Investigators taking verbal consent must confirm that they are speaking to the correct individual by having the patient confirm their name, address and date of birth (DOB). The investigator must provide an oral explanation of the purpose of GD2 testing, and the consequences of the reported results, which may permit participation in the main CARPETS study. It will be the investigator's responsibility to confirm that the patient has understood the explanation, and that the patient is willing to verbally consent to participate in the Pre-Screening component of the study.

Preparation of the ICF is the responsibility of the Investigator and must include all elements required by the International Conference on Harmonization (ICH), Good Clinical Practice (GCP), and to the ethical principles that have their origin in the Declaration of Helsinki.

Gargett T, *et al. J Immunother Cancer* 2024; 12:e008659. doi: 10.1136/jitc-2023-008659

The Investigator must provide the patient or a legal representative with a copy of the ICF and written information about the study in the language in which the patient is most proficient. The language must be non-technical and easily understood. The Investigator should allow time necessary for patient or patient's legal representative to inquire about the details of the study after which the ICF must be signed and personally dated by the patient or the patient's legally acceptable representative and by the person who conducted the informed consent discussion. The patient or a legal representative should receive a copy of the signed ICF and any other written information provided to study patients prior to patient's participation in the trial.

13.4 Records and Reports

All records will be kept confidential and the patient's name will not be released at any time. Patient records will not be released to anyone other than the responsible government agencies. In all cases, caution will be exercised to assure the patient's confidentiality. Data sets for each patient will be identified by a unique number.

The Investigator is required to prepare and maintain adequate and accurate case histories designed to record all observations and other data pertinent to the investigation on each individual treated with the investigational product.

13.5 Ethics Committee

An ICF will be prepared according to the institutional requirements for informed consent and the applicable regulations. The appropriate Ethics Committee must approve the protocol and ICF documents, agree to monitor the conduct of the study, and agree to review study progress periodically, at intervals not to exceed 1 year. The investigator will provide the office of the Royal Adelaide Hospital General Manager or its designee with documentation that the Ethics Committee has approved the study before the study may begin.

In addition, the investigator will provide the office of the Royal Adelaide Hospital General Manager or its designee with documentation of all approvals:

1. The Ethics Committee's periodic re-approval of the protocol, per current Title 21 CFR 312.66 regulations and current ICH guidelines.
2. The Ethics Committee's approval of revisions to the ICF documents or any amendments to the protocol. Any revisions to the protocol that may increase patient risk exposure must be reviewed by the Ethics Committee and approved prior to implementation. Administrative changes (such as a change in address or phone number) must be sent to Ethics Committees but do not require their approval.

13.6 Study Records

Copies of all CRFs and study-related documentation must be retained at the site. The Investigator shall maintain the records of drug disposition, final CRFs, worksheets and all other study-specific documentation (e.g., Study File notebooks, source documentation) for 15 years.

13.6.1 Case Report Form Completion

The Principal Investigator, or designee, is responsible for recording all data from the study on the CRFs. The data on each CRF must be legibly handwritten with a black ballpoint pen or

Gargett T, *et al. J Immunother Cancer* 2024; 12:e008659. doi: 10.1136/jitc-2023-008659

typed. Data reported on the CRF, which are derived from source documents, must be consistent with the source documents or the discrepancies must be explained.

Patients are to be identified by initials, birth date, and patient number, if applicable. All requested information must be entered on the CRF in the spaces provided. If an item is not available or is not applicable, it must be documented as such; do not leave a space blank. A correction must be made by striking through the incorrect entry with a single line and entering the correct information adjacent to the incorrect entry. Each correction must be dated, initialed, and explained in the comment section on the patient's CRF (if necessary) by the person making the correction and must not obscure the original entry.

Each page of the CRF should be reviewed by the Principal Investigator. The Principal Investigator is required to sign the CRF on the appropriate page(s) to verify that he/she has reviewed the recorded data. This review and sign-off may be delegated to a qualified physician appointed as a Sub-Investigator by the Principal Investigator. The transfer of duties must be recorded on the Delegation List (kept on file at the site). The Investigator must ensure that all Sub-Investigators are familiar with the protocol and all study-specific procedures and have appropriate knowledge of the study agent(s).

By signing the protocol, the Investigator acknowledges that, within legal and regulatory restrictions and institutional and ethical considerations, Royal Adelaide Hospital, its designee, or responsible government agencies (as required by law) may, at any time, review or copy source documents (e.g., laboratory reports, electrocardiograms, x-rays, workbooks and patients' medical records) in order to verify CRF data.

13.7 Warnings, Precautions, and Contraindications

For specific information concerning warnings, precautions, and contraindications, the Investigator is asked to refer to the appropriate section of the GD2-iCAR Investigator's Brochure.

13.8 Modification of Protocol

The study shall be conducted as described in this approved protocol. The Investigator should not implement any deviation or change to the protocol without prior review and documented approval from the Ethics Committee of an amendment, except where necessary to eliminate an immediate hazard(s) to study patients. Any significant deviation must be documented in the CRF.

If a deviation or change to a protocol is implemented to eliminate an immediate hazard(s) prior to obtaining Ethics Committee approval/favorable opinion, as soon as possible the deviation or change will be submitted to:

Ethics Committee for review and approval/favourable opinion.

Regulatory authority(ies), if required by local regulations

13.9 Criteria for Termination of the Study

Both the Sponsor and the Investigator reserve the right to terminate the study at any time. Should this be necessary, the procedures will be arranged on an individual study basis after review and consultation by both parties. In terminating the study, the Sponsor and the

Gargett T, *et al. J Immunother Cancer* 2024; 12:e008659. doi: 10.1136/jitc-2023-008659

Investigator will ensure that adequate consideration is given to the protection of the patient's interests.

This study will be complete when the maximum tolerated dose has been determined as per Protocol Design or 12 patients have been entered into the study whichever is sooner (see Section 6).

The study may be terminated prematurely if the Principal Investigator, DSMB and Sponsor feel that the number and/or severity of adverse events justify discontinuation of the study.

14. PUBLICATION POLICY

The results of this study will be disseminated *via* the RAH Cancer Centre website, the Australian and New Zealand Melanoma Trials Group (ANZMTG) website, *via* presentations at national and international conferences, and *via* publication in the peer-reviewed international scientific literature.

Gargett T, *et al. J Immunother Cancer* 2024; 12:e008659. doi: 10.1136/jitc-2023-008659

15. REFERENCES

(1) AIHW (Australian Institute of Health and Welfare) & AACR (Australasian Association of CancerRegistries). Cancer in Australia: an overview, 2008. 2008. Canberra, AIHW. Cancer series no. 46. Cat. no. CAN 42.

(2) Australian Institute of Health and Welfare. Australia's health 2008. [Cat. no. AUS 99.]. 2008. Canberra, AIHW.

(3) Hodi FS, O'Day SJ, McDermott DF, Weber RW, Sosman JA, Haanen JB, et al. Improved survival with ipilimumab in patients with metastatic melanoma. N Engl J Med 2010 Aug 19;363(8):711-23.

(4) Curtin JA, Busam K, Pinkel D, Bastian BC. Somatic activation of KIT in distinct subtypes of melanoma. Journal of clinical oncology : official journal of the American Society of Clinical Oncology 2006 Sep 10;24(26):4340-6.

(5) Davies H, Bignell GR, Cox C, Stephens P, Edkins S, Clegg S, et al. Mutations of the BRAF gene in human cancer. Nature 2002 Jun 27;417(6892):949-54.

(6) Goel VK, Lazar AJ, Warneke CL, Redston MS, Haluska FG. Examination of mutations in BRAF, NRAS, and PTEN in primary cutaneous melanoma. J Invest Dermatol 2006 Jan;126(1):154-60.

(7) Weinstein IB, Joe A. Oncogene addiction. Cancer Res 2008 May 1;68(9):3077-80.

(8) Riesco-Eizaguirre G, Rodriguez I, De l, V, Costamagna E, Carrasco N, Nistal M, et al. The BRAFV600E oncogene induces transforming growth factor beta secretion leading to sodium iodide symporter repression and increased malignancy in thyroid cancer. Cancer Res 2009 Nov 1;69(21):8317-25.

(9) Flockhart RJ, Armstrong JL, Reynolds NJ, Lovat PE. NFAT signalling is a novel target of oncogenic BRAF in metastatic melanoma. Br J Cancer 2009 Oct 20;101(8):1448-55.

(10) Sumimoto H, Imabayashi F, Iwata T, Kawakami Y. The BRAF-MAPK signaling pathway is essential for cancer-immune evasion in human melanoma cells. J Exp Med 2006 Jul 10;203(7):1651-6.

(11) Cavallo F, De GC, Nanni P, Forni G, Lollini PL. 2011: the immune hallmarks of cancer. Cancer Immunol Immunother 2011 Mar;60(3):319-26.

(12) Flaherty KT, Puzanov I, Kim KB, Ribas A, McArthur GA, Sosman JA, et al. Inhibition of mutated, activated BRAF in metastatic melanoma. N Engl J Med 2010 Aug 26;363(9):809-19.

(13) Chapman PB, Hauschild A, Robert C, Haanen JB, Ascierto P, Larkin J, et al. Improved survival with vemurafenib in melanoma with BRAF V600E mutation. N Engl J Med 2011 Jun 30;364(26):2507-16.

(14) Vriesendorp FJ, Quadri SM, Flynn RE, Malone MR, Cromeens DM, Stephens LC, et al. Preclinical analysis of radiolabeled anti-GD2 immunoglobulin G. Cancer 1997 Dec 15;80(12 Suppl):2642-9.

(15) Klein O, Clements A, Menzies AM, O'Toole S, Kefford RF, Long GV. BRAF inhibitor activity in V600R metastatic melanoma - Response. Eur J Cancer 2013 Mar 9;49(5):1073-9.

(16) Hauschild A, Grob JJ, Demidov LV, Jouary T, Gutzmer R, Millward M, et al. Dabrafenib in BRAF-mutated metastatic melanoma: a multicentre, open-label, phase 3 randomised controlled trial. Lancet 2012 Jul 28;380(9839):358-65.

(17) Roth M, Linkowski M, Tarim J, Piperdi S, Sowers R, Geller D, et al. Ganglioside GD2 as a therapeutic target for antibody-mediated therapy in patients with osteosarcoma. Cancer 2014 Feb 15;120(4):548-54.

(18) Chan MM, Haydu LE, Menzies AM, Azer MW, Klein O, Lyle M, et al. The nature and management of metastatic melanoma after progression on BRAF inhibitors: Effects of extended BRAF inhibition. Cancer 2014 Jul 1.

(19) Shi H, Hugo W, Kong X, Hong A, Koya RC, Moriceau G, et al. Acquired resistance and clonal evolution in melanoma during BRAF inhibitor therapy. Cancer Discov 2014 Jan;4(1):80-93.

(20) Van Allen EM, Wagle N, Sucker A, Treacy DJ, Johannessen CM, Goetz EM, et al. The genetic landscape of clinical resistance to RAF inhibition in metastatic melanoma. Cancer Discov 2014 Jan;4(1):94-109.

(21) Flaherty KT. BRAF inhibitors and melanoma. Cancer J 2011 Nov;17(6):505-11.

(22) Villanueva J, Vultur A, Herlyn M. Resistance to BRAF inhibitors: unraveling mechanisms and future treatment options. Cancer Res 2011 Dec 1;71(23):7137-40.

(23) Poulikakos PI, Rosen N. Mutant BRAF melanomas--dependence and resistance. Cancer Cell 2011 Jan 18;19(1):11-5.

(24) Solit D, Sawyers CL. Drug discovery: How melanomas bypass new therapy. Nature 2010 Dec 16;468(7326):902-3.

(25) Straussman R, Morikawa T, Shee K, Barzily-Rokni M, Qian ZR, Du J, et al. Tumour micro-environment elicits innate resistance to RAF inhibitors through HGF secretion. Nature 2012 Jul 4.

(26) Long GV, Stroyakovskiy D, Gogas H, Levchenko E, de BF, Larkin J, et al. Combined BRAF and MEK inhibition versus BRAF inhibition alone in melanoma. N Engl J Med 2014 Nov 13;371(20):1877-88.

(27) Wagle N, Van Allen EM, Treacy DJ, Frederick DT, Cooper ZA, Taylor-Weiner A, et al. MAP kinase pathway alterations in BRAF-mutant melanoma patients with acquired resistance to combined RAF/MEK inhibition. Cancer Discov 2014 Jan;4(1):61-8.

(28) Long GV, Fung C, Menzies AM, Pupo GM, Carlino MS, Hyman J, et al. Increased MAPK reactivation in early resistance to dabrafenib/trametinib combination therapy of BRAF-mutant metastatic melanoma. Nat Commun 2014;5:5694.

(29) Kelderman S, Heemskerk B, van TH, van den Brom RR, Hospers GA, van den Eertwegh AJ, et al. Lactate dehydrogenase as a selection criterion for ipilimumab treatment in metastatic melanoma. Cancer Immunol Immunother 2014 May;63(5):449-58.

(30) Robert C, Thomas L, Bondarenko I, O'Day S, JW MD, Garbe C, et al. Ipilimumab plus dacarbazine for previously untreated metastatic melanoma. N Engl J Med 2011 Jun 30;364(26):2517-26.

(31) Robert C, Schachter J, Long GV, Arance A, Grob JJ, Mortier L, et al. Pembrolizumab versus Ipilimumab in Advanced Melanoma. N Engl J Med 2015 Jun 25;372(26):2521-32.

(32) Larkin J, Chiarion-Sileni V, Gonzalez R, Grob JJ, Cowey CL, Lao CD, et al. Combined Nivolumab and Ipilimumab or Monotherapy in Untreated Melanoma. N Engl J Med 2015;373(1).

(33) Long GV, Trefzer U, Davies MA, Kefford RF, Ascierto PA, Chapman PB, et al. Dabrafenib in patients with Val600Glu or Val600Lys BRAF-mutant melanoma metastatic to the brain (BREAK-MB): a multicentre, open-label, phase 2 trial. Lancet Oncol 2012 Nov;13(11):1087-95.

(34) Margolin K, Ernstoff MS, Hamid O, Lawrence D, McDermott D, Puzanov I, et al. Ipilimumab in patients with melanoma and brain metastases: an open-label, phase 2 trial. Lancet Oncol 2012 May;13(5):459-65.

(35) Falchook GS, Long GV, Kurzrock R, Kim KB, Arkenau TH, Brown MP, et al. Dabrafenib in patients with melanoma, untreated brain metastases, and other solid tumours: a phase 1 dose-escalation trial. Lancet 2012 May 19;379(9829):1893-901.

(36) Larkin J, Del VM, Ascierto PA, Krajsova I, Schachter J, Neyns B, et al. Vemurafenib in patients with BRAF(V600) mutated metastatic melanoma: an open-label, multicentre, safety study. Lancet Oncol 2014 Apr;15(4):436-44.

(37) Sosman JA, Kim KB, Schuchter L, Gonzalez R, Pavlick AC, Weber JS, et al. Survival in BRAF V600-mutant advanced melanoma treated with vemurafenib. N Engl J Med 2012 Feb 23;366(8):707-14.

Gargett T, *et al. J Immunother Cancer* 2024; 12:e008659. doi: 10.1136/jitc-2023-008659

(38) Chapman PB, Hauschild A, Robert C, Haanen JB, Ascierto P, Larkin J, et al. Improved survival with vemurafenib in melanoma with BRAF V600E mutation. N Engl J Med 2011 Jun 30;364(26):2507-16.

(39) Ascierto PA, Simeone E, Grimaldi AM, Curvietto M, Esposito A, Palmieri G, et al. Do BRAF inhibitors select for populations with different disease progression kinetics? J Transl Med 2013;11:61.

(40) Ackerman A, Klein O, McDermott DF, Wang W, Ibrahim N, Lawrence DP, et al. Outcomes of patients with metastatic melanoma treated with immunotherapy prior to or after BRAF inhibitors. Cancer 2014 Jun 1;120(11):1695-701.

(41) Kirkwood JM, Tarhini AA, Panelli MC, Moschos SJ, Zarour HM, Butterfield LH, et al. Next generation of immunotherapy for melanoma. Journal of clinical oncology : official journal of the American Society of Clinical Oncology 2008 Jul 10;26(20):3445-55.

(42) Clemente CG, Mihm MC, Jr., Bufalino R, Zurrida S, Collini P, Cascinelli N. Prognostic value of tumor infiltrating lymphocytes in the vertical growth phase of primary cutaneous melanoma. Cancer 1996 Apr 1;77(7):1303-10.

(43) Mihm MC, Jr., Clemente CG, Cascinelli N. Tumor infiltrating lymphocytes in lymph node melanoma metastases: a histopathologic prognostic indicator and an expression of local immune response. Lab Invest 1996 Jan;74(1):43-7.

(44) Lee PP, Yee C, Savage PA, Fong L, Brockstedt D, Weber JS, et al. Characterization of circulating T cells specific for tumor-associated antigens in melanoma patients. Nat Med 1999 Jun;5(6):677-85.

(45) Yee C. Adoptive T-cell therapy of cancer. Hematol Oncol Clin North Am 2006 Jun;20(3):711-33.

(46) Rosenberg SA. The immunotherapy of solid cancers based on cloning the genes encoding tumor-rejection antigens. Annu Rev Med 1996;47:481-91.

(47) Houghton AN, Vijayasaradhi S, Bouchard B, Naftzger C, Hara I, Chapman PB. Recognition of autoantigens by patients with melanoma. Ann N Y Acad Sci 1993 Aug 12;690:59-68.

(48) Rosenberg SA, Dudley ME. Adoptive cell therapy for the treatment of patients with metastatic melanoma. Curr Opin Immunol 2009 Apr;21(2):233-40.

(49) Suzuki M, Cheung NK. Disialoganglioside GD2 as a therapeutic target for human diseases. Expert Opin Ther Targets 2015 Mar;19(3):349-62.

(50) Cheresh DA, Harper JR, Schulz G, Reisfeld RA. Localization of the gangliosides GD2 and GD3 in adhesion plaques and on the surface of human melanoma cells. Proc Natl Acad Sci U S A 1984 Sep;81(18):5767-71.

Gargett T, *et al. J Immunother Cancer* 2024; 12:e008659. doi: 10.1136/jitc-2023-008659

(51) Hersey P, Jamal O, Henderson C, Zardawi I, D'Alessandro G. Expression of the gangliosides GM3, GD3 and GD2 in tissue sections of normal skin, naevi, primary and metastatic melanoma. Int J Cancer 1988 Mar 15;41(3):336-43.

(52) Albino AP, Sozzi G, Nanus DM, Jhanwar SC, Houghton AN. Malignant transformation of human melanocytes: induction of a complete melanoma phenotype and genotype. Oncogene 1992 Nov;7(11):2315-21.

(53) Ravindranath MH, Muthugounder S, Presser N. Ganglioside signatures of primary and nodal metastatic melanoma cell lines from the same patient. Melanoma Res 2008 Feb;18(1):47-55.

(54) Livingston PO, Wong GY, Adluri S, Tao Y, Padavan M, Parente R, et al. Improved survival in stage III melanoma patients with GM2 antibodies: a randomized trial of adjuvant vaccination with GM2 ganglioside. Journal of clinical oncology : official journal of the American Society of Clinical Oncology 1994 May;12(5):1036-44.

(55) Cheung NK, Kushner BH, Cheung IY, Kramer K, Canete A, Gerald W, et al. Anti-G(D2) antibody treatment of minimal residual stage 4 neuroblastoma diagnosed at more than 1 year of age. J Clin Oncol 1998 Sep;16(9):3053-60.

(56) Handgretinger R, Anderson K, Lang P, Dopfer R, Klingebiel T, Schrappe M, et al. A phase I study of human/mouse chimeric antiganglioside GD2 antibody ch14.18 in patients with neuroblastoma. Eur J Cancer 1995;31A(2):261-7.

(57) Yvon E, Del VM, Savoldo B, Hoyos V, Dutour A, Anichini A, et al. Immunotherapy of metastatic melanoma using genetically engineered GD2-specific T cells. Clin Cancer Res 2009 Sep 15;15(18):5852-60.

(58) Dobrenkov K, Ostrovnaya I, Gu J, Cheung IY, Cheung NK. Oncotargets GD2 and GD3 are highly expressed in sarcomas of children, adolescents, and young adults. Pediatr Blood Cancer 2016 Oct;63(10):1780-5.

(59) Chang HR, Cordon-Cardo C, Houghton AN, Cheung NK, Brennan MF. Expression of disialogangliosides GD2 and GD3 on human soft tissue sarcomas. Cancer 1992 Aug 1;70(3):633-8.

(60) Heiner JP, Miraldi F, Kallick S, Makley J, Neely J, Smith-Mensah WH, et al. Localization of GD2-specific monoclonal antibody 3F8 in human osteosarcoma. Cancer Res 1987 Oct 15;47(20):5377-81.

(61) Modak S, Gerald W, Cheung NK. Disialoganglioside GD2 and a novel tumor antigen: potential targets for immunotherapy of desmoplastic small round cell tumor. Med Pediatr Oncol 2002 Dec;39(6):547-51.

(62) Grant SC, Kostakoglu L, Kris MG, Yeh SD, Larson SM, Finn RD, et al. Targeting of small-cell lung cancer using the anti-GD2 ganglioside monoclonal antibody 3F8: a pilot trial. Eur J Nucl Med 1996 Feb;23(2):145-9.

(63) Kailayangiri S, Altvater B, Meltzer J, Pscherer S, Luecke A, Dierkes C, et al. The ganglioside antigen G(D2) is surface-expressed in Ewing sarcoma and allows for MHC-independent immune targeting. Br J Cancer 2012 Mar 13;106(6):1123-33.

(64) Ziebarth AJ, Felder MA, Harter J, Connor JP. Uterine leiomyosarcoma diffusely express disialoganglioside GD2 and bind the therapeutic immunocytokine 14.18-IL2: implications for immunotherapy. Cancer Immunol Immunother 2012 Jul;61(7):1149-53.

(65) Orsi G, Barbolini M, Ficarra G, Tazzioli G, Manni P, Petrachi T, et al. GD2 expression in breast cancer. Oncotarget 2017 May 9;8(19):31592-600.

(66) Cheresh DA, Rosenberg J, Mujoo K, Hirschowitz L, Reisfeld RA. Biosynthesis and expression of the disialoganglioside GD2, a relevant target antigen on small cell lung carcinoma for monoclonal antibody-mediated cytolysis. Cancer Res 1986 Oct;46(10):5112-8.

(67) Yu AL, Gilman AL, Ozkaynak MF, London WB, Kreissman SG, Chen HX, et al. Anti-GD2 antibody with GM-CSF, interleukin-2, and isotretinoin for neuroblastoma. N Engl J Med 2010 Sep 30;363(14):1324-34.

(68) Dhillon S. Dinutuximab: first global approval. Drugs 2015 May;75(8):923-7.

(69) Hwu P, Shafer GE, Treisman J, Schindler DG, Gross G, Cowherd R, et al. Lysis of ovarian cancer cells by human lymphocytes redirected with a chimeric gene composed of an antibody variable region and the Fc receptor gamma chain. J Exp Med 1993;178:361-6.

(70) Romeo C, Seed B. Cellular immunity to HIV activated by CD4 fused to T cell or Fc receptor polypeptides. Cell 1991 Mar 8;64(5):1037-46.

(71) Irving BA, Weiss A. The cytoplasmic domain of the T cell receptor zeta chain is sufficient to couple to receptor-associated signal transduction pathways. Cell 1991 Mar 8;64(5):891-901.

(72) Letourneur F, Klausner RD. T-cell and basophil activation through the cytoplasmic tail of T-cell- receptor zeta family proteins. Proc Natl Acad Sci U S A 1991 Oct 15;88(20):8905-9.

(73) Weijtens ME, Willemsen RA, Valerio D, Stam K, Bolhuis RL. Single chain Ig/gamma gene-redirected human T lymphocytes produce cytokines, specifically lyse tumor cells, and recycle lytic capacity. J Immunol 1996 Jul 15;157(2):836-43.

(74) Moritz D, Wels W, Mattern J, Groner B. Cytotoxic T lymphocytes with a grafted recognition specificity for ERBB2-expressing tumor cells. Proc Natl Acad Sci U S A 1994 May 10;91(10):4318-22.

(75) Hwu P, Shafer GE, Treisman J, Schindler DG, Gross G, Cowherd R, et al. Lysis of ovarian cancer cells by human lymphocytes redirected with a chimeric gene composed of an antibody variable region and the Fc receptor gamma chain. J Exp Med 1993 Jul 1;178(1):361-6.

(76) McGuinness RP, Ge Y, Patel SD, Kashmiri SV, Lee HS, Hand PH, et al. Anti-tumor activity of human T cells expressing the CC49-zeta chimeric immune receptor [see comments]. Hum Gene Ther 1999 Jan 20;10(2):165-73.

(77) Krause A, Guo HF, Latouche JB, Tan C, Cheung NK, Sadelain M. Antigen-dependent CD28 signaling selectively enhances survival and proliferation in genetically modified activated human primary T lymphocytes. J Exp Med 1998 Aug 17;188(4):619-26.

(78) Brocker T. Chimeric Fv-zeta or Fv-epsilon receptors are not sufficient to induce activation or cytokine production in peripheral T cells. Blood 2000 Sep 1;96(5):1999-2001.

(79) Pule MA, Straathof KC, Dotti G, Heslop HE, Rooney CM, Brenner MK. A chimeric T cell antigen receptor that augments cytokine release and supports clonal expansion of primary human T cells. Molecular Therapy: the Journal of the American Society of Gene Therapy 2005 Nov;12(5):933-41.

(80) Vera J, Savoldo B, Vigouroux S, Biagi E, Pule M, Rossig C, et al. T lymphocytes redirected against the kappa light chain of human immunoglobulin efficiently kill mature B lymphocyte-derived malignant cells. Blood 2006 Dec 1;108(12):3890-7.

(81) Foon KA, Sen G, Hutchins L, Kashala OL, Baral R, Banerjee M, et al. Antibody responses in melanoma patients immunized with an anti-idiotype antibody mimicking disialoganglioside GD2. Clin Cancer Res 1998 May;4(5):1117-24.

(82) Wilmott JS, Long GV, Howle JR, Haydu LE, Sharma RN, Thompson JF, et al. Selective BRAF inhibitors induce marked T-cell infiltration into human metastatic melanoma. Clin Cancer Res 2012 Mar 1;18(5):1386-94.

(83) Boni A, Cogdill AP, Dang P, Udayakumar D, Njauw CN, Sloss CM, et al. Selective BRAFV600E inhibition enhances T-cell recognition of melanoma without affecting lymphocyte function. Cancer Res 2010 Jul 1;70(13):5213-9.

(84) Frederick DT, Piris A, Cogdill AP, Cooper ZA, Lezcano C, Ferrone CR, et al. BRAF inhibition is associated with enhanced melanoma antigen expression and a more favorable tumor microenvironment in patients with metastatic melanoma. Clin Cancer Res 2013 Mar 1;19(5):1225-31.

(85) Schilling B, Sucker A, Griewank K, Zhao F, Weide B, Gorgens A, et al. Vemurafenib reverses immunosuppression by myeloid derived suppressor cells. Int J Cancer 2013 Oct 1;133(7):1653-63.

(86) Comin-Anduix B, Chodon T, Sazegar H, Matsunaga D, Mock S, Jalil J, et al. The oncogenic BRAF kinase inhibitor PLX4032/RG7204 does not affect the viability or function of human lymphocytes across a wide range of concentrations. Clin Cancer Res 2010 Dec 15;16(24):6040-8.

(87) Hong DS, Vence L, Falchook G, Radvanyi LG, Liu C, Goodman V, et al. BRAF(V600) inhibitor GSK2118436 targeted inhibition of mutant BRAF in cancer patients does not impair overall immune competency. Clin Cancer Res 2012 Apr 15;18(8):2326-35.

(88) Wilmott JS, Haydu LE, Menzies AM, Lum T, Hyman J, Thompson JF, et al. Dynamics of Chemokine, Cytokine, and Growth Factor Serum Levels in BRAF-Mutant Melanoma Patients during BRAF Inhibitor Treatment. J Immunol 2014 Jan 31.

(89) Schilling B, Sondermann W, Zhao F, Griewank KG, Livingstone E, Sucker A, et al. Differential influence of vemurafenib and dabrafenib on patients' lymphocytes despite similar clinical efficacy in melanoma. Ann Oncol 2014 Mar;25(3):747-53.

(90) Ribas A, Hodi FS, Callahan M, Konto C, Wolchok J. Hepatotoxicity with combination of vemurafenib and ipilimumab. N Engl J Med 2013 Apr 4;368(14):1365-6.

(91) Puzanov I, Callahan MK, Linette GP, Patel SP, Luke JJ, Sosman JA, et al. Phase 1 study of the BRAF inhibitor dabrafenib (D) with or without the MEK inhibitor trametinib (T) in combination with ipilimumab (Ipi) for V600E/K mutation-positive unresectable or metastatic melanoma (MM). J Clin Oncol (Meeting Abstracts) 2014 Jun 11;32(15_suppl):2511.

(92) Hu-Lieskovan S, Mok S, Robert Faja L, Goedert L, Comin-Anduix B, Koya RC, et al. Combinatorial effect of dabrafenib, trametinib, and adoptive cell transfer (ACT) in an immune-competent murine model of BRAFV600E mutant melanoma. J Clin Oncol (Meeting Abstracts) 2014 Jun 11;32(15_suppl):2512.

(93) Schumacher-Kuckelkorn R, Hero B, Ernestus K, Berthold F. Lacking immunocytological GD2 expression in neuroblastoma: report of 3 cases. Pediatr Blood Cancer 2005 Aug;45(2):195-201.

(94) Gargett T, Fraser CK, Dotti G, Yvon ES, Brown MP. BRAF and MEK inhibition variably affect GD2-specific chimeric antigen receptor (CAR) T-cell function in vitro. J Immunother 2015 Jan;38(1):12-23.

(95) Hu-Lieskovan S, Robert L, Homet MB, Ribas A. Combining Targeted Therapy With Immunotherapy in BRAF-Mutant Melanoma: Promise and Challenges. J Clin Oncol 2014 Jun 23.

(96) Liu L, Mayes PA, Eastman S, Shi H, Yadavilli S, Zhang T, et al. The BRAF and MEK Inhibitors Dabrafenib and Trametinib: Effects on Immune Function and in Combination with Immunomodulatory Antibodies Targeting PD1, PD-L1 and CTLA-4. Clin Cancer Res 2015 Jan 14.

(97) Di Stasi A., Tey SK, Dotti G, Fujita Y, Kennedy-Nasser A, Martinez C, et al. Inducible apoptosis as a safety switch for adoptive cell therapy. N Engl J Med 2011 Nov 3;365(18):1673-83.

(98) Myers GD, Pule M, Russell H, Liu E, Weiss H, Dotti G, et al. Virus-specific T cells engineered to co-express tumor-specific receptors; effects in patients with neuroblastoma. Biol Blood Marrow Transplant Suppl 2, 153. 2007.

(99) Pule MA, Savoldo B, Myers GD, Rossig C, Russell HV, Dotti G, et al. Virus-specific T cells engineered to coexpress tumor-specific receptors: persistence and antitumor activity in individuals with neuroblastoma. Nat Med 2008 Nov;14(11):1264-70.

(100) Laport GG, Levine BL, Stadtmauer EA, Schuster SJ, Luger SM, Grupp S, et al. Adoptive transfer of costimulated T cells induces lymphocytosis in patients with relapsed/refractory non-Hodgkin's lymphoma following CD34- selected hematopoietic cell transplantation. Blood 2003 May 22;102(6):2004-13.

(101) Rapoport AP, Stadtmauer EA, Aqui N, Badros A, Cotte J, Chrisley L, et al. Restoration of immunity in lymphopenic individuals with cancer by vaccination and adoptive T-cell transfer. Nat Med 2005 Nov;11(11):1230-7.

(102) Rooney CM, Smith CA, Ng CYC, Loftin SK, Sixbey JW, Gan Y-J, et al. Infusion of cytotoxic T cells for the prevention and treatment of Epstein-Barr virus-induced lymphoma in allogeneic transplant recipients. Blood 1998;92(5):1549-55.

(103) Heslop HE, Ng CYC, Li C, Smith CA, Loftin SK, Krance RA, et al. Long-term restoration of immunity against Epstein-Barr virus infection by adoptive transfer of gene-modified virus-specific T lymphocytes. Nature Medicine 1996;2:551-5.

(104) Rooney CM, Smith CA, Ng C, Loftin SK, Li C, Krance RA, et al. Use of gene-modified virus-specific T lymphocytes to control Epstein-Barr virus-related lymphoproliferation. Lancet 1995;345:9-13.

(105) Bollard CM, Aguilar L, Straathof KC, Gahn B, Huls MH, Rousseau A, et al. Cytotoxic T Lymphocyte Therapy for Epstein-Barr Virus+ Hodgkin's Disease. J Exp Med 2004 Dec 20;200(12):1623-33.

(106) Bollard CM, Gottschalk S, Leen AM, Weiss H, Straathof KC, Carrum G, et al. Complete responses of relapsed lymphoma following genetic modification of tumor-antigen presenting cells and T-lymphocyte transfer. Blood 2007 Oct 15;110(8):2838-45.

(107) Straathof KC, Bollard CM, Popat U, Huls MH, Lopez T, Morriss MC, et al. Treatment of Nasopharyngeal Carcinoma with Epstein-Barr Virus-specific T Lymphocytes. Blood 2005 Mar 1;105:1898-904.

Gargett T, *et al. J Immunother Cancer* 2024; 12:e008659. doi: 10.1136/jitc-2023-008659

(108) Louis CU, Straathof K, Bollard CM, Gerken C, Huls MH, Gresik MV, et al. Enhancing the in vivo expansion of adoptively transferred EBV-specific CTL with lymphodepleting CD45 monoclonal antibodies in NPC patients. Blood 2009 Mar 12;113(11):2442-50.

(109) Louis CU, Savoldo B, Dotti G, Pule M, Yvon E, Myers GD, et al. Anti-tumor activity and long-term fate of chimeric antigen receptor positive T-cells in patients with neuroblastoma. Blood 2011 Oct 7.

(110) Kershaw MH, Westwood JA, Parker LL, Wang G, Eshhar Z, Mavroukakis SA, et al. A phase I study on adoptive immunotherapy using gene-modified T cells for ovarian cancer. Clin Cancer Res 2006 Oct 15;12(20 Pt 1):6106-15.

(111) Park JR, Digiusto DL, Slovak M, Wright C, Naranjo A, Wagner J, et al. Adoptive transfer of chimeric antigen receptor re-directed cytolytic T lymphocyte clones in patients with neuroblastoma. Mol Ther 2007 Apr;15(4):825-33.

(112) Till BG, Jensen MC, Wang J, Chen EY, Wood BL, Greisman HA, et al. Adoptive immunotherapy for indolent non-Hodgkin lymphoma and mantle cell lymphoma using genetically modified autologous CD20-specific T cells. Blood 2008 May 28.

(113) Porter DL, Levine BL, Kalos M, Bagg A, June CH. Chimeric antigen receptor-modified T cells in chronic lymphoid leukemia. N Engl J Med 2011 Aug 25;365(8):725-33.

(114) Kalos M, Levine BL, Porter DL, Katz S, Grupp SA, Bagg A, et al. T cells with chimeric antigen receptors have potent antitumor effects and can establish memory in patients with advanced Leukemia. Sci Transl Med 2011 Aug 10;3(95):95ra73.

(115) Morgan RA, Yang JC, Kitano M, Dudley ME, Laurencot CM, Rosenberg SA. Case report of a serious adverse event following the administration of T cells transduced with a chimeric antigen receptor recognizing ERBB2. Mol Ther 2010 Apr;18(4):843-51.

(116) Brentjens R, Yeh R, Bernal Y, Riviere I, Sadelain M. Treatment of chronic lymphocytic leukemia with genetically targeted autologous T cells: case report of an unforeseen adverse event in a phase I clinical trial. Mol Ther 2010 Apr;18(4):666-8.

(117) Straathof KC, Pule MA, Yotnda P, Dotti G, Vanin EF, Brenner MK, et al. An inducible caspase 9 safety switch for T-cell therapy. Blood 2005 Jun 1;105(11):4247-54.

(118) Clackson T, Yang W, Rozamus LW, Hatada M, Amara JF, Rollins CT, et al. Redesigning an FKBP-ligand interface to generate chemical dimerizers with novel specificity. Proc Natl Acad Sci U S A 1998 Sep 1;95(18):10437-42.

(119) Donnelly ML, Hughes LE, Luke G, Mendoza H, ten DE, Gani D, et al. The 'cleavage' activities of foot-and-mouth disease virus 2A site-directed mutants and naturally occurring '2A-like' sequences. J Gen Virol 2001 May;82(Pt 5):1027-41.

Gargett T, *et al. J Immunother Cancer* 2024; 12:e008659. doi: 10.1136/jitc-2023-008659

(120) Donnelly ML, Luke G, Mehrotra A, Li X, Hughes LE, Gani D, et al. Analysis of the aphthovirus 2A/2B polyprotein 'cleavage' mechanism indicates not a proteolytic reaction, but a novel translational effect: a putative ribosomal 'skip'. J Gen Virol 2001 May;82(Pt 5):1013-25.

(121) Iuliucci JD, Oliver SD, Morley S, Ward C, Ward J, Dalgarno D, et al. Intravenous safety and pharmacokinetics of a novel dimerizer drug, AP1903, in healthy volunteers. J Clin Pharmacol 2001 Aug;41(8):870-9.

(122) Tey SK, Dotti G, Rooney CM, Heslop HE, Brenner MK. Inducible caspase 9 suicide gene to improve the safety of allodepleted T cells after haploidentical stem cell transplantation. Biol Blood Marrow Transplant 2007 Aug;13(8):913-24.

(123) Li Z, Dullmann J, Schiedlmeier B, Schmidt M, von Kalle C, Meyer J, et al. Murine leukemia induced by retroviral gene marking. Science 2002 Apr 19;296(5567):497.

(124) Vanin EF, Kaloss M, Broscius C, Nienhuis AW. Characterization of replication-competent retroviruses from nonhuman primates with virus-induced T-cell lymphomas and observations regarding the mechanism of oncogenesis. J Virol 1994 Jul;68(7):4241-50.

(125) Hacein-Bey-Abina S, Garrigue A, Wang GP, Soulier J, Lim A, Morillon E, et al. Insertional oncogenesis in 4 patients after retrovirus-mediated gene therapy of SCID-X1. J Clin Invest 2008 Sep;118(9):3132-42.

(126) Dave UP, Akagi K, Tripathi R, Cleveland SM, Thompson MA, Yi M, et al. Murine leukemias with retroviral insertions at Lmo2 are predictive of the leukemias induced in SCID-X1 patients following retroviral gene therapy. PLoS Genet 2009 May;5(5):e1000491.

(127) Uribe L, Weinberg KI. X-linked SCID and other defects of cytokine pathways. Semin Hematol 1998 Oct;35(4):299-309.

(128) Puck JM, Krauss CM, Puck SM, Buckley RH, Conley ME. Prenatal test for X-linked severe combined immunodeficiency by analysis of maternal X-chromosome inactivation and linkage analysis. N Engl J Med 1990 Apr 12;322(15):1063-6.

(129) Havenga M, Hoogerbrugge P, Valerio D, van Es HH. Retroviral stem cell gene therapy. Stem Cells 1997;15(3):162-79.

(130) Brenner MK. Gene transfer and the treatment of haematological malignancy. J Intern Med 2001 Apr;249(4):345-58.

(131) Brenner MK, Rill DR, Moen RC, Krance RA, Mirro J, Jr., Anderson WF, et al. Gene-marking to trace origin of relapse after autologous bone marrow transplantation. Lancet 1993;341:85-6.

(132) Irie RF, Morton DL. Regression of cutaneous metastatic melanoma by intralesional injection with human monoclonal antibody to ganglioside GD2. Proc Natl Acad Sci U S A 1986 Nov;83(22):8694-8.

(133) Cheung NV, Lazarus H, Miraldi FD, Berger NA, Abramowsky CR, Saarinen UM, et al. Reassessment of patient response to monoclonal antibody 3F8. J Clin Oncol 1992 Apr;10(4):671-2.

(134) Saleh MN, Khazaeli MB, Wheeler RH, Allen L, Tilden AB, Grizzle W, et al. Phase I trial of the chimeric anti-GD2 monoclonal antibody ch14.18 in patients with malignant melanoma. Hum Antibodies Hybridomas 1992 Jan;3(1):19-24.

(135) Murray JL, Kleinerman ES, Jia SF, Rosenblum MG, Eton O, Buzaid A, et al. Phase Ia/Ib trial of anti-GD2 chimeric monoclonal antibody 14.18 (ch14.18) and recombinant human granulocyte-macrophage colony-stimulating factor (rhGM-CSF) in metastatic melanoma. J Immunother Emphasis Tumor Immunol 1996 May;19(3):206-17.

(136) Niethammer D, Handgretinger R. Clinical strategies for the treatment of neuroblastoma. Eur J Cancer 1995;31A(4):568-71.

(137) Frost JD, Hank JA, Reaman GH, Frierdich S, Seeger RC, Gan J, et al. A phase I/IB trial of murine monoclonal anti-GD2 antibody 14.G2a plus interleukin-2 in children with refractory neuroblastoma: a report of the Children's Cancer Group. Cancer 1997 Jul 15;80(2):317-33.

(138) Yu AL, Uttenreuther-Fischer MM, Huang CS, Tsui CC, Gillies SD, Reisfeld RA, et al. Phase I trial of a human-mouse chimeric anti-disialoganglioside monoclonal antibody ch14.18 in patients with refractory neuroblastoma and osteosarcoma. J Clin Oncol 1998 Jun;16(6):2169-80.

(139) Cheung NK, Kushner BH, LaQuaglia M, Kramer K, Gollamudi S, Heller G, et al. N7: a novel multi-modality therapy of high risk neuroblastoma (NB) in children diagnosed over 1 year of age. Med Pediatr Oncol 2001 Jan;36(1):227-30.

(140) O'Quigley J, Pepe M, Fisher L. Continual reassessment method: a practical design for phase 1 clinical trials in cancer. Biometrics 1990 Mar;46(1):33-48.

(141) O'Quigley J, Shen LZ. Continual reassessment method: a likelihood approach. Biometrics 1996 Jun;52(2):673-84.

(142) Heczey A, Louis CU, Savoldo B, Dakhova O, Durett A, Grilley B, et al. CAR T Cells Administered in Combination with Lymphodepletion and PD-1 Inhibition to Patients with Neuroblastoma. Mol Ther 2017 Sep 6;25(9):2214-24.

(143) Rooney CM, Smith CA, Ng CY, Loftin S, Li C, Krance RA, et al. Use of gene-modified virus-specific T lymphocytes to control Epstein-Barr-virus-related lymphoproliferation. Lancet 1995 Jan 7;345(8941):9-13.

(144) Eisenhauer EA, Therasse P, Bogaerts J, Schwartz LH, Sargent D, Ford R, et al. New response evaluation criteria in solid tumours: revised RECIST guideline (version 1.1). Eur J Cancer 2009 Jan;45(2):228-47.

(145) Aloia AL, Duffy L, Pak V, Lee KE, Sanchez-Martinez S, Derse D, et al. A reporter system for replication-competent gammaretroviruses: the inGluc-MLV-DERSE assay. Gene Ther 2013 Feb;20(2):169-76.

(146) Bowman LC, Grossmann M, Rill D, Brown M, Zhong WY, Alexander B, et al. Interleukin-2 gene-modified allogeneic tumor cells for treatment of relapsed neuroblastoma. Hum Gene Ther 1998 Jun 10;9(9):1303-11.

(147) Hersey P, Honeyman M, Edwards A, Adams E, McCarthy WH. Antigens on melanoma cells detected by leukocyte dependent antibody assays of human melanoma antisera. Int J Cancer 1976 Nov 15;18(5):564-73.

CONFIDENTIAL

16. APPENDIX I – CTC V.4.03

http://evs.nci.nih.gov/ftp1/CTCAE/CTCAE_4.03_2010-06-14_QuickReference_5x7.pdf

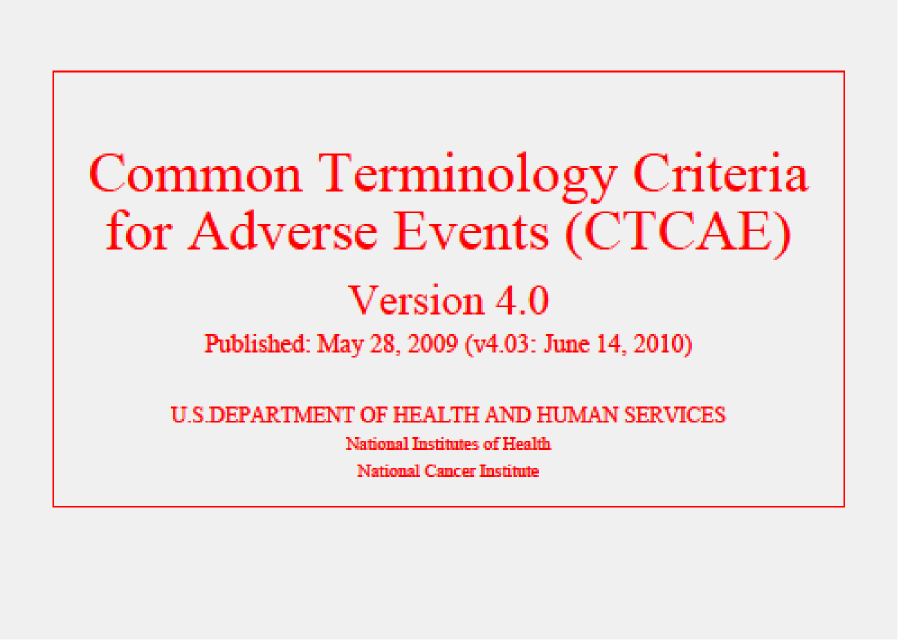

Common Terminology Criteria for Adverse Events (CTCAE)

Version 4.0

Published: May 28, 2009 (v4.03: June 14, 2010)

U.S.DEPARTMENT OF HEALTH AND HUMAN SERVICES
National Institutes of Health
National Cancer Institute

Gargett T, *et al. J Immunother Cancer* 2024; 12:e008659. doi: 10.1136/jitc-2023-008659

17. APPENDIX II – Effects of Dabrafenib on Concomitant Medications

Substrates	
**CYP 2C9**[1]	**CYP3A4**[1]
**NSAIDs:**<br>diclofenac<br>ibuprofen<br>lornoxicam<br>meloxicam<br>S-naproxen<br>piroxicam<br>suprofen<br>**Oral Hypoglycemic:**<br>tolbutamide<br>glipizide<br>Angiotensin II Blockers:<br>losartan<br>irbesartan<br>Sulfonylureas:<br>glyburide<br>glibenclamide<br>glipizide<br>glimepiride<br>tolbutamide<br>amitriptyline<br>celecoxib<br>fluoxetine<br>fluvastatin<br>glyburide<br>nateglinide<br>phenytoin-4-OH2<br>rosiglitazone<br>tamoxifen<br>torsemide<br>S-warfarin	**Macrolide antibiotics:**<br>clarithromycin<br>erythromycin<br>telithromycin<br>Anti-arrhythmics:<br>quinidine<br>**Benzodiazepines:**<br>alprazolam<br>diazepam<br>midazolam<br>triazolam<br>**Immune Modulators:**<br>cyclosporine<br>tacrolimus (FK506)<br>**HIV Antivirals:**<br>indinavir<br>nelfinavir<br>ritonavir<br>saquinavir<br>**Prokinetic:**<br>cisapride<br>**Antihistamines:**<br>astemizole<br>chlorpheniramine<br>terfenadine<br>**Calcium Channel Blockers:**<br>amlodipine<br>diltiazem<br>felodipine<br>lercanidipine<br>nifedipine2<br>nisoldipine<br>nitrendipine<br>verapamil<br>**HMG CoA Reductase Inhibitors:**<br>atorvastatin<br>cerivastatin<br>lovastatin<br>simvastatin<br>**Steroid 6beta-OH:**<br>estradiol

	hydrocortisone<br>progesterone<br>testosterone<br>**Miscellaneous:**<br>alfentanyl<br>aprepitant<br>aripiprazole<br>buspirone<br>cafergot<br>caffeine<br>cilostazol<br>cocaine<br>codeine-Ndemethylation<br>dapsone<br>dexamethasone<br>dextromethorphan<br>docetaxel<br>domperidone<br>eplerenone<br>fentanyl<br>finasteride<br>gleevec<br>haloperidol<br>irinotecan<br>lidocaine<br>methadone<br>nateglinide<br>ondansetron<br>pimozide<br>propranolol<br>quetiapine<br>quinine<br>risperidone<br>salmeterol<br>sildenafil<br>sirolimus<br>tamoxifen<br>taxol<br>terfenadine<br>trazodone<br>vincristine<br>zaleplon<br>ziprasidone<br>zolpidem

[1]Exposure of these drugs may be decreased following dabrafenib treatment
=

CONFIDENTIAL

Effect of concomitant medications on Dabrafenib[3]			
**CYP3A4 Inhibitors[1]**	**CYP3A4 Inducers[2]**	**CYP2C8 Inhibitors[1]**	**CYP2C8 Inducers[2]**
**HIV Antivirals:**<br>indinavir<br>nelfinavir<br>ritonavir<br><br>clarithromycin<br>itraconazole<br>ketoconazole<br>nefazodone<br>saquinavir<br>telithromycin<br>aprepitant<br>erythromycin<br>fluconazole<br>grapefruit juice<br>verapamil<br>diltiazem<br>cimetidine<br>amiodarone<br>chloramphenicol<br>ciprofloxacin<br>delaviridine<br>diethyldithiocarbamate<br>fluvoxamine<br>gestodene<br>imatinib<br>mibefradil<br>mifepristone<br>norfloxacin<br>norfluoxetine<br>star fruit<br>voriconazole	**HIV Antivirals:**<br>efavirenz<br>nevirapine<br><br>barbiturates<br>carbamazepine<br>efavirenz<br>glucocorticoids<br>modafinil<br>nevirapine<br>oxcarbazepine<br>phenobarbital<br>phenytoin<br>pioglitazone<br>rifabutin<br>rifampin<br>St. John's wort<br>troglitazone	gemfibrozil<br>trimethoprim<br>glitazones<br>montelukast<br>quercetin	rifampin

[1]Concomitant administration of these drugs may increase dabrafenib exposure
[2]Concomitant administration of these drugs may reduce dabrafenib exposure
[3]Dabrafenib has exhibited very low metabolism and the effect of CYP3A4 inhibitors or inducers may be minimal

Reference: http://medicine.iupui.edu/clinpharm/ddis/

CONFIDENTIAL

18. APPENDIX III – Hypersensitivity Reactions

MANAGEMENT OF ACUTE HYPERSENSITIVITY

**Severity of Symptoms**	**Treatment Guidelines**
**Mild** symptoms: localized cutaneous reactions such as mild pruritus, flushing, rash	· consider decreasing the rate of injection until recovery from symptoms, stay at bedside and monitor patient then, complete injection at the initial planned rate
**Moderate** symptoms: any symptom that is not listed above (mild symptoms) or below (severe symptoms) such as generalized pruritus, flushing, rash, dyspnea, hypotension with systolic BP > 80 mm Hg	· interrupt GD2-iCAR-PBT injection and give promethazine 25 mg IV; monitor patient until resolution of symptoms<br>· resume administration as a very slow iv push (e.g. over 5 minutes) after recovery of symptoms; depending on the physician's assessment of the patient,<br>· depending on the intensity of the reaction observed, additional oral or IV premedication with an antihistamine should also be given for the **next cycle** of treatment if planned.
**Severe** symptoms: any reaction such as bronchospasm, generalized urticaria, systolic BP ≤ 80mm Hg, angioedema	· immediately discontinue injection<br>· give promethazine 25 mg and/or adrenaline as needed; monitor patient until resolution of symptoms, the same treatment guidelines outlined under moderate symptoms (i.e. the third and fourth bullets) should be followed.
**Anaphylaxis** (NCI grade 4 reaction)	· treat as above<br>· NO FURTHER STUDY DRUG THERAPY

Gargett T, *et al. J Immunother Cancer* 2024; 12:e008659. doi: 10.1136/jitc-2023-008659

19. APPENDIX IV – HIV/Hepatitis Test Discussion Checklist

PRE-TEST CHECKLIST

At least the following (but not limited to) are to be discussed in the HIV / hepatitis discussion with patients:

1. Risk assessment ☐
2. Reason for testing ☐
3. Information about confidentiality and privacy ☐
4. HIV testing – how results will be provided and the window period ☐
   Hepatitis testing – what a positive, negative and indeterminate result means
5. What happens to test results – legislative requirement to report ☐
6. Explanation of informed consent ☐
7. What a positive result means in regards to:
   - Medical aspects including advantages of early interventions ☐
   - Referral to specialised treatment centres ☐
   - Psychological aspects ☐
   - Notification requirements ☐
   - Social aspects and the need for support to be in place ☐
8. What a negative result means ☐
9. Assess the patient's preparedness for testing ☐
10. Assessment of support mechanisms while waiting for test results ☐
11. Preventative aspects whatever the test result
    - Safe sex practices ☐
    - Safe needle and syringe use ☐

Gargett T, *et al. J Immunother Cancer* 2024; 12:e008659. doi: 10.1136/jitc-2023-008659

20. APPENDIX V – Revised Cytokine Release Syndrome (CRS) Grading System

Grade	Toxicity
Grade 1	Symptoms are not life threatening and require symptomatic treatment only, eg, fever, nausea, fatigue, headache, myalgias, malaise
Grade 2	Symptoms require and respond to moderate intervention<br>Oxygen requirement $<$40% or<br>Hypotension responsive to fluids or low dose[2] of one vasopressor or<br>Grade 2 organ toxicity
Grade 3	Symptoms require and respond to aggressive intervention<br>Oxygen requirement $\geq$40% or<br>Hypotension requiring high dose* or multiple vasopressors or<br>Grade 3 organ toxicity or grade 4 transaminitis
Grade 4	Life-threatening symptoms<br>Requirement for ventilator support or<br>Grade 4 organ toxicity (excluding transaminitis)
Grade 5	Death

Grades 2-4 refer to CTCAE v4.0 grading.

From DW Lee et al. Current concepts in the diagnosis and management of cytokine release syndrome. *Blood* 2014;124(2):188-195.

Gargett T, *et al. J Immunother Cancer* 2024; 12:e008659. doi: 10.1136/jitc-2023-008659
